# Embedding shared decision-making in the care of patients with severe and enduring mental health problems: The EQUIP pragmatic cluster randomised trial

**DOI:** 10.1371/journal.pone.0201533

**Published:** 2018-08-22

**Authors:** Karina Lovell, Penny Bee, Helen Brooks, Patrick Cahoon, Patrick Callaghan, Lesley-Anne Carter, Lindsey Cree, Linda Davies, Richard Drake, Claire Fraser, Chris Gibbons, Andrew Grundy, Kathryn Hinsliff-Smith, Oonagh Meade, Chris Roberts, Anne Rogers, Kelly Rushton, Caroline Sanders, Gemma Shields, Lauren Walker, Peter Bower

**Affiliations:** 1 Division of Nursing, Midwifery & Social Work, School of Health Sciences, University of Manchester, Manchester, United Kingdom; 2 Department of Psychological Sciences, Institute of Psychology, Health and Society, University of Liverpool, Liverpool, United Kingdom; 3 Greater Manchester Mental Health NHS Foundation Trust, Manchester, United Kingdom; 4 School of Applied Sciences, London South Bank University, London, United Kingdom; 5 Division of Population Health, Health Services Research & Primary Care, School of Health Sciences, University of Manchester, Manchester, United Kingdom; 6 Division of Psychology & Mental Health, School of Health Sciences, University of Manchester, Manchester, United Kingdom; 7 Healthcare Improvement Studies Institute, University of Cambridge, Cambridge, United Kingdom; 8 School of Health Sciences, University of Nottingham, Nottingham, United Kingdom; 9 School of Psychology, National University of Ireland Galway, Galway, Ireland; 10 Faculty of Health Sciences, University of Southampton, Southampton, United Kingdom; 11 Greater Manchester Mental Health NHS Foundation Trust, Manchester, United Kingdom; TNO, NETHERLANDS

## Abstract

**Background:**

Severe mental illness is a major driver of worldwide disease burden. Shared decision-making is critical for high quality care, and can enhance patient satisfaction and outcomes. However, it has not been translated into routine practice. This reflects a lack of evidence on the best way to implement shared decision-making, and the challenges of implementation in routine settings with limited resources. Our aim was to test whether we could deliver a practical and feasible intervention in routine community mental health services to embed shared decision-making for patients with severe mental illness, by improving patient and carer involvement in care planning.

**Methods:**

We cluster randomised community mental health teams to the training intervention or usual care, to avoid contamination. Training was co-delivered to a total of 350 staff in 18 teams by clinical academics, working alongside patients and carers. The primary outcome was the Health Care Climate Questionnaire, a self-report measure of ‘autonomy support’. Primary and secondary outcomes were collected by self-report, six months after allocation.

**Findings:**

In total, 604 patients and 90 carers were recruited to main trial cohort. Retention at six months was 82% (n = 497). In the main analysis, results showed no statistically significant difference in the primary outcome between the intervention and usual care at 6 months (adjusted mean difference -0.064, 95% CI -0.343 to 0.215, p = 0.654). We found significant effects on only 1 secondary outcome.

**Conclusions:**

An intervention to embed shared decision-making in routine practice by improving involvement in care planning was well attended and acceptable to staff, but had no significant effects on patient outcomes. Enhancing shared decision-making may require considerably greater investment of resources and effects may only be apparent over the longer term.

## Introduction

Mental health conditions impact substantially on quality of life and productivity, and are a major driver of worldwide disease burden. The global cost of mental illness was estimated at 2.5 trillion US dollars (£1.9 trillion / 2.1 trillion Euros) in 2010, set to rise to 6 trillion US dollars (£4.6 trillion/5 trillion Euros) in two decades. Improving mental health care is an international priority [1].

Shared decision-making is an integral component of high quality care for both physical and mental health. Shared decision-making has been defined as ‘an approach where clinicians and patients share the best available evidence when faced with the task of making decisions, and where patients are supported to consider options, to achieve informed preferences’ [2]. Shared decision-making ensures that patients are active in decisions, that services focus on patient needs, and that professionals are better able to provide patient-centred care. Interventions in shared decision-making can increase the quality and safety of healthcare and enhance patient satisfaction, treatment adherence and outcomes [3].

Shared decision-making is recognised as a guiding principle of mental health policy and practice [4]. In the UK, mental health professionals providing care for patients with serious mental illnesses (such as psychosis) act as care co-ordinators under the Care Programme Approach which is mandated for patients with severe and enduring mental illness [5,6]. The Care Programme Approach involves an assessment of patient needs, choices about care and support, family and financial issues [7], followed by production of a care plan developed between professionals, the patient and (where relevant) their carer [5,6,8].

There is broad consensus among stakeholders regarding the importance of shared decision-making in mental health, although debates continue [4,9]. Yet, despite a sustained policy emphasis, this has not been translated into practice [10]. A national survey of 7500 patients by the Care Quality Commission in the UK found that only 34% agreed that they were ‘definitely’ involved as much as they wanted to be in decisions [11,12]. The Government Five Year Forward View for Mental Health highlighted co-produced care planning as a key goal and a recommended Care Quality Commission quality standard by 2020 [13].

Although there is a large literature on patient decision aids, the evidence on how to encourage shared decision-making in mental health is far more limited [14]. A recent review of studies to enhance shared decision-making in psychosis found 11 trials of a variety of interventions, with some evidence of effects on measures of ‘subjective empowerment’. However, studies were generally small and of only modest quality. Furthermore, most delivered interventions to patients (an approach which may be more difficult to implement at scale), and only one study tested training for clinicians [15]. At present, there is no proven method of implementing shared decision-making across routine mental health settings.

The recent MAGIC study explored how shared decision-making could be embedded within the UK NHS, and identified many challenges. These include misplaced confidence amongst staff about their current practice, a perceived lack of tools to support shared decision-making and inaccurate assumptions about patients’ preferences [16,17]. MAGIC examined a number of clinical contexts, but excluded mental health, where shared decision-making faces particular challenges, reflecting a unique history founded on concepts of containment and coercion [18]. Patients often present with long term and complex diagnoses, and experience significant stigma [19,20]. Our recent review of shared decision-making in mental health identified similar challenges to those found in MAGIC, including the readiness, skills and confidence of mental health professionals to engage [21]. Our review also highlighted different frames of reference for mental health professionals and patients, with patients’ judgments of success heavily influenced by the consistency and quality of care planning relationships, whereas staff highlighted the quality of the end product of shared decision-making (the written care plan).

MAGIC concluded that ‘implementing shared decision making is challenging but possible’[17]. Although some shared decision-making interventions have demonstrated effectiveness [15], these often involve selected settings with local enthusiasts around specific clinical decisions. We do not know how best to deliver change in a comprehensive way to embed shared decision-making across routine clinical services facing resource and time constraints. Our aim was to test whether we could deliver a practical and feasible intervention in routine community mental health services to embed shared decision-making by improving patient and carer involvement in the care planning process.

## Methods

### Design

**Fig 1:**
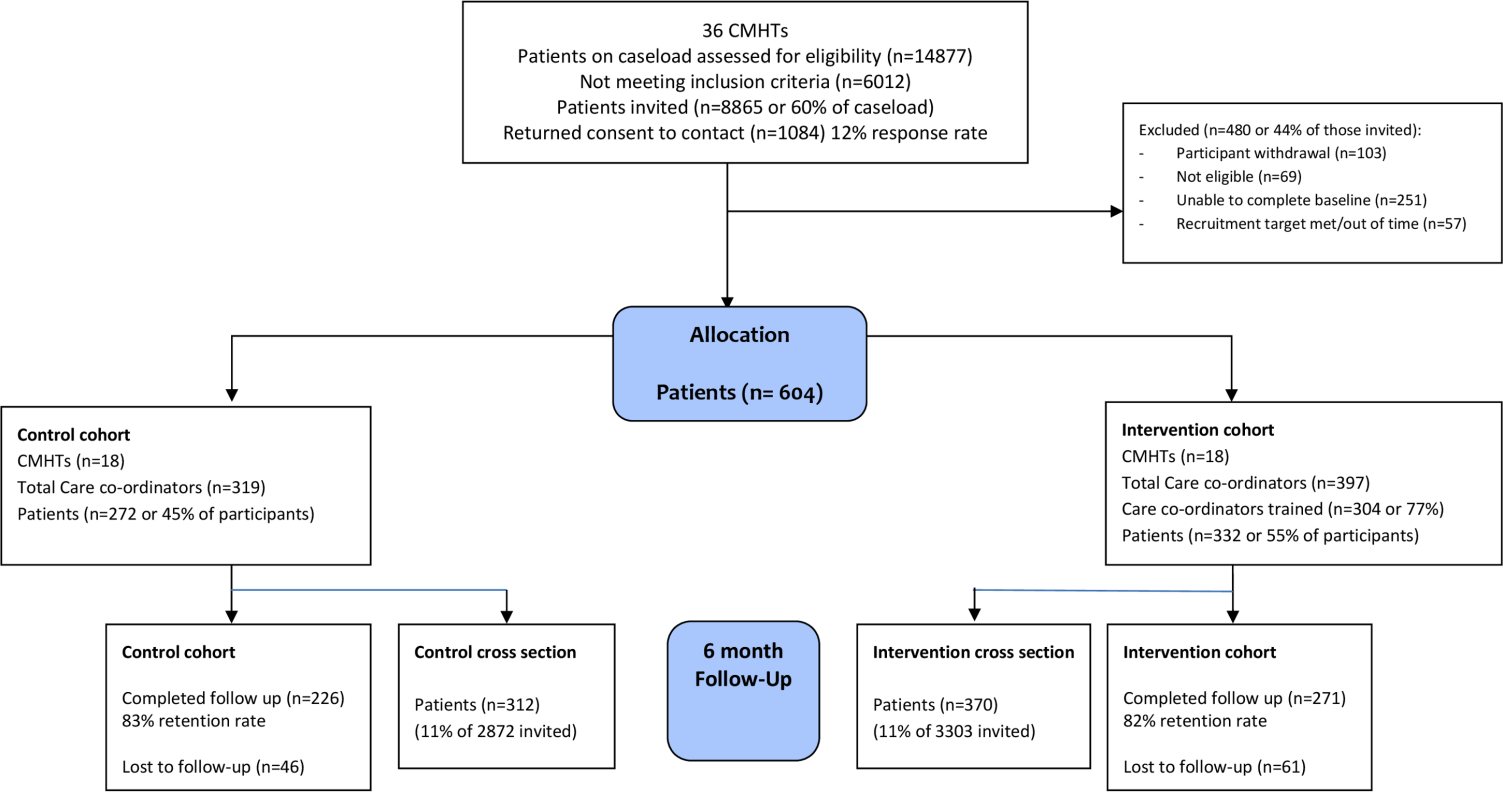
Consolidated standards of reporting trials (CONSORT) for cluster and cross sectional cohorts.

The primary design was a pragmatic ‘cluster cohort’ randomised trial. The intervention was targeted at staff and we randomised teams to avoid contamination. Following cluster randomisation, we recruited patients cared for by teams of community mental health professionals in both arms (see Fig 1). We assessed those patients at 6 months, comparing outcomes in the teams trained to embed shared decision-making in their routine care planning, with control teams delivering routine care planning alone. Our trial protocol has been published [22, S1 Protocol] and the trial was registered (ISRCTN16488358). The full trial protocol is supplied as a supporting information file (S2 Protocol).

In terms of the PRECIS-2 criteria [23], we enhanced the pragmatic nature of our trial by adopting very broad patient inclusion criteria, delivering the intervention in routine care settings, limiting the resources required to deliver the intervention, allowing flexibility in delivery and adherence, using an outcome of direct relevance to patients, and adopting intention to treat analysis.

### Participants and recruitment

We recruited community mental health teams and patients between July 2014 and December 2015 from 10 NHS Trusts across England, UK. All teams within the participating NHS Trusts were eligible to take part, and eligible patients were aged 18+ with a severe and enduring mental illness (including psychosis, bipolar disorder, schizophrenia, personality disorder). We excluded patients without capacity to provide fully informed consent, and those too unwell at recruitment, as judged by the teams. Patients were followed up six months after baseline measures were completed between January 2015 and July 2016.

During recruitment, we became aware that the number of patients per cluster was smaller than estimated in the sample size calculation. We increased the number of clusters from 12 to 18 per arm to ensure sufficient power. In total, 604 patients and 90 carers were recruited to the cluster cohort as detailed in the CONSORT (Fig 1). The mean number of patients recruited per team was 16.8 (SD 8.7, range 4–43). Retention at six months was 82% (n = 497).

**Fig 2:**
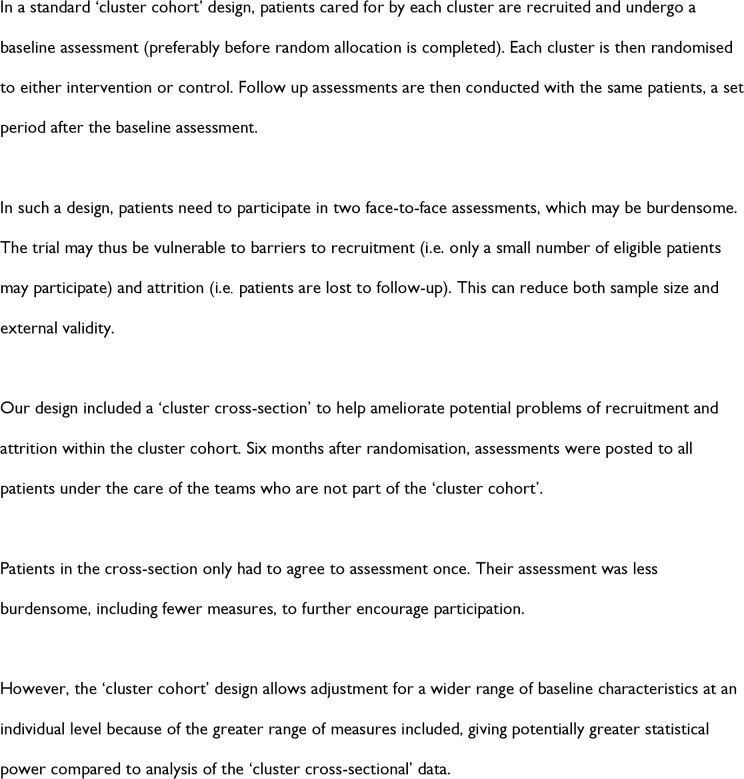
Cluster design.

We additionally included a ‘cluster cross-section’ sample to reduce the risk associated with loss to follow up in the patient sample in the cluster cohort design (see Fig 2). This involved recruiting patients under the care of teams in both arms at follow up only.

Patient flow in the cluster cross section is also shown in the CONSORT (Fig 1). In total, 682 patients were recruited with a mean number of patients per team of 18.9 (SD 14.1, range 2–62).

### Intervention

Shared decision-making is a complex concept [24], and its interpretation in terms of the nature and degree of participation differs across and within lay and professional groups [25]. Much of the literature concerns ‘patient decision aids’, tools which focus on providing support for ‘specific and deliberated choices among healthcare options’ [3], often relating to specific conditions and management choices. Some aids are designed to be used by patients outside clinical consultations to support later discussions with professionals. Others (so-called *conversation aids*) are ‘designed to encourage and directly support the conversations that patients and clinicians have when making decisions together’ [26]. The intervention described here was a type of conversation aid, to enhance the ability of staff to involve patients in decisions [27], and to embed shared decision-making into the existing care planning process in mental health services.

**Fig 3:**
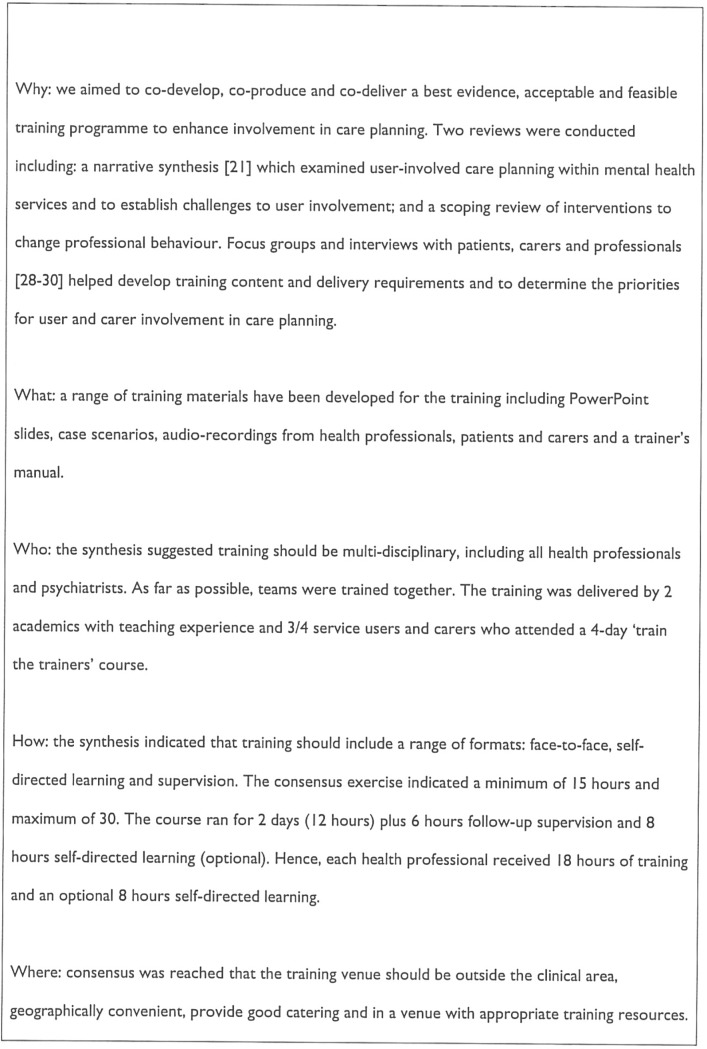
Intervention components according to the TIDieR guidelines.

We designed an intervention to enhance shared decision-making in routine community mental health services, where there is limited time and resource for training and quality improvement. This required a compromise between effectiveness, acceptability to patients and professionals, and feasibility. Fig 3 describes our intervention according to the TIDIER guidelines.

To inform training content and format, we synthesised evidence from a systematic review of service user involvement in care-planning [21] and a scoping review of interventions to improve professional practice and change patient care. Focus groups and individual interviews with patients (n = 42), carers (n = 37) and health professionals (n = 51) were used to sensitise the training to the context of routine mental health services [28–31].

Training content was designed to counter negative attitudes to patient and carer involvement, enhance shared decision-making, and introduce these skills into time-limited interactions.

Training was co-delivered to teams by two clinical academics and patients and carers. We considered two days training feasible given current service pressures [31]. We involved patients in the design and delivery of the intervention, sought organisational ‘buy-in’ through engagement with Trust chief executives, senior managers and front line staff, and used role play to challenge entrenched attitudes and foster skills. Training materials included a trainer’s manual, PowerPoint slides, case scenarios, and audio-recordings from health professionals and patients. Patient and carers delivering the training attended a four-day ‘train the trainers’ course [32]. We used the Training Acceptability Rating Scale [33,34] immediately after training to assess staff experience of the intervention [35].

### Procedure

Teams were introduced to the trial by letters or meetings with senior managers. Meetings were held with team managers to facilitate study engagement.

To recruit patients into the cluster cohort, research officers from local clinical research networks sent patients an introductory letter, information sheet and ‘consent to contact’ form. Patients opted in by returning the ‘consent to contact’ form to the study team. Non-responders were contacted by telephone once to improve response rates. Where possible, consenting patients were asked to nominate a carer, who was provided with appropriate information, consent materials and carer measures.

To recruit to the cluster cross-section, we conducted a postal survey of all patients under the care of each team six months after randomisation (excluding those already in the cluster cohort).

Once patients and carers had been sent invitations, clusters were allocated randomly to either intervention or control. Allocation was determined by an external telephone randomisation service at the Clinical Trials Unit of the Manchester Academic Health Science Centre. Clusters from the same geographical area were submitted to the randomisation service in pairs. No further matching of these pairs on other characteristics prior to allocation was possible in practice [22].

Sites were recruited and trained in a rolling programme over 17 months. Although it is preferable to complete patient recruitment entirely before allocation, clusters needed advanced notice of training dates. We ensured that invitations were sent to patients prior to site knowledge of their allocation, reducing the risk of professionals influencing selective recruitment.

Researchers blind to allocation assisted participants in completing measures at baseline and six-months. Demographic data, primary, secondary and economic outcome measures were collected face-to-face for the cluster cohort and by post for the cluster cross-section and carer samples. Telephone and postal completion was also available (on request) for the cluster cohort during follow up data collection which took place between January 2015 and July 2016, with telephone completion involving reading out the scales to patients.

All teams allocated to the intervention received the shared decision-making intervention consisting of two days face-to-face training (12 hours total), an eight-hour optional self-directed learning package and six hours supervision per team in the six months after training. We asked that at least 80% of staff designated as ‘care co-ordinators’ (i.e., those with a caseload) attended training. As far as possible, multi-disciplinary teams were trained together. Training was delivered within 6 weeks of patient recruitment. Usual care teams did not have access to the training.

### Outcomes

All outcomes were collected by self-report, six months after allocation.

Our aim was to embed shared decision-making by improving patient and carer involvement in the care planning process. The primary outcome was the Health Care Climate Questionnaire (HCCQ-10), a self-report scale based on self-determination theory [36]. The HCCQ-10 measures ‘autonomy support’, defined as patient perceptions of the degree to which they experience their health professionals as supporting choice, and ensuring their behaviour (and behaviour change) is congruent with their values. According to self-determination theory, autonomy support is more likely to lead to behaviour change and improved health outcomes [37]. The scale has ten items, examples of which include: ‘I feel that my mental health care provider team has provided me with choices and options’; and ‘My mental health care provider team has worked with me to develop a mental health care plan’. Items are scored on a 7 point scale from ‘strongly disagree’ to ‘strongly agree’. An overall score is calculated as the mean of the items, with a higher score indicating greater ‘autonomy support’.

Secondary outcomes were chosen by experts and a consensus exercise with our patient advisory group. Patient perceptions of involvement in care planning decisions were assessed via a newly developed and validated 61 item self-report measure (EQUIP PROM). The measure provides a unidimensional measure of service user and carer involvement in mental health care planning and has since been further revised to produce a short-form 14-item scale [38]. We also measured patient satisfaction with mental health services (Verona Service Satisfaction Scale—VSSS-EU-54) [39,40]; patient-reported side-effects of antipsychotic medication (Glasgow Antipsychotic Side-effect Scale—GASS) [41]; mental well-being (Warwick-Edinburgh Mental Well-being Scale -WEMWBS) [42]; recovery and hope (Developing Recovery Enhancing Environments Measure—DREEM) [43]; anxiety and depression (Hospital Anxiety and Depression Scale—HADS) [44]; alliance and engagement (California Psychotherapy Alliance Scale -CALPAS) [45]; a single item measure of global quality of life from a scale (World Health Organisation Quality of Life—WHOQOL-BREF) [46]. Carer satisfaction was measured via the Carers and Users’ Expectations of Services—carer version (CUES-C) [47]: carers also completed the short form of the EQUIP PROM and the WHOQOL-BREF. The EQ-5D-5L [48] was used to estimate Quality-Adjusted Life Years (QALYs), alongside a questionnaire on frequency of use of health and social care services. Economic outcomes will be reported separately.

The UK National Research Ethics Service (NRES Committee North West Lancaster) approved this study (REC Reference 14/NW/0297). All participants gave informed consent before taking part.

### Sample size and statistical methods

The primary outcome was the HCCQ-10, prioritised by our patient advisory group. Data on the HCCQ-10 in this patient population was limited, so we used a standardised effect to calculate sample size. A trial with 12 clusters per arm, a mean of 20 patients per cluster (total n = 480) would have power greater than 80% to detect a standardised effect size of 0.4, assuming an intra-cluster correlation coefficient of 0.05 and 80% follow up (n = 384 patients with complete data). We felt that this effect size was plausible when assessing an outcome such as patient perceptions of autonomy support, which would be more amenable to change than clinical outcomes. Power is increased by the inclusion of baseline covariates. For the cross-sectional component, we aimed to recruit at least the same number of patients per cluster, to provide the same power.

Analysis was completed using Stata 13 [49] and followed a statistical analysis plan prepared prior to analysis and approved by the independent programme steering group. The plan identified the cluster cohort as the primary analysis, with the cluster cross section and combined analyses to be presented as secondary analyses. For the cluster cohort, intervention effects were estimated using a linear mixed model with a random intercept for teams. Analysis of outcomes followed intention-to-treat principles with outcome data included for all patients irrespective of receipt of the intervention or completion of care planning during the trial. We adopted a 5% level for statistical significance.

## Results

As there were no substantial differences in results between cluster cohort and cluster cross section samples, we present detailed results only for the former in the text. Full data on the cluster cross section and carers are found in the S1 Table.

### Baseline characteristics

Table 1 shows baseline characteristics of the teams and patients included in the cluster cohort, and Table 2 shows baseline scores on study measures. Characteristics were similar between intervention and usual care. Characteristics of patients in the cluster cross section and carers are presented in the S1 Table. The sample included a range of self-reported mental health disorders, including, depression (47%), anxiety (32%), bipolar (25%), schizophrenia (23%), personality disorder (17%), panic disorders (9%), eating disorder (6%) and phobia (5%).

**Table 1:** Baseline characteristics of patients in the cluster cohort.

		Control (n = 272)		Intervention (n = 332)	
		n	%	n	%
**Age**	18–24	17	6.25	21	6.33
	25–44	99	36.40	114	34.34
	45–64	134	49.26	177	53.31
	65+	16	5.88	11	3.31
	Missing	6	2.21	9	2.71
**Gender**	Female	157	57.72	198	59.64
	Male	106	38.97	128	38.55
	Other	1	0.37		
	Missing	8	2.94	6	1.81
**Ethnic Group**	White	233	85.66	294	88.55
	Non-White	33	12.13	32	9.64
	Missing	6	2.21	6	1.81
**Education**	Secondary school	108	39.71	129	38.86
	Higher education	154	56.62	181	54.52
	Missing	10	3.68	22	6.63
**Accommodation**	Owner occupier	85	31.25	97	29.22
	Other	177	65.07	225	67.77
	Missing	10	3.68	10	3.01
**Living Arrangements**	Alone or with a pet	175	64.34	207	62.35
	With someone else	92	33.82	119	35.84
	Missing	5	1.84	6	1.81
**Employment**	Employed	37	13.60	45	13.55
	Other	231	84.93	280	84.64
	Missing	4	1.47	7	2.11
		**Median**	**IQR (n)**	**Median**	**IQR (n)**
Time experiencing mental health problems (months)		228	84–360 (265)	204	120–313 (320)
Time using NHS services (months)		109.5	42–252 (262)	121.5	52–240 (318)

**Table 2:** Baseline measures in cluster cohort.

	Control					Intervention					ICC
	Mean	SD	Min	Max	n	Mean	SD	Min	Max	n	
HCCQ-10	5.06	1.65	1	7	272	5.27	1.49	1	7	329	0.001
EQUIP PROM	22.58	9.68	0	44	214	21.99	9.73	0	44	250	0.011
HADS Anxiety	11.37	5.63	0	21	243	12.32	5.52	0	21	288	0.035
HADS Depression	9.18	5.57	0	21	243	10.04	5.51	0	21	288	0.061
VSSS-54	3.56	0.70	1.45	4.85	203	3.54	0.68	1.54	4.89	259	0.029
CALAPS-12	4.96	1.43	1.33	7	209	5.06	1.36	1	7	252	0.000
GASS	17.78	11.70	0	54	191	18.09	10.71	0	49.42	226	0.000
WHOQOL	3.02	1.14	1	5	209	3.03	1.18	1	5	255	0.020
DREEM	41.30	12.34	24	89	150	42.11	12.15	24	77	170	0.008
WEMWBS	39.12	13.55	14	70	221	38.65	13.04	14	70	264	0.045

### Intervention delivery

We delivered the intervention to staff in 18 teams drawn from 10 NHS trust sites. The training cohort comprised 350 professionals of whom 304 were care coordinators (nurses, occupational therapists and social workers), along with pre-registration students, support workers and clinical managers (n = 46). Ten of 18 teams met our request to send 80% of care coordinators at the training, with an overall mean of 77% of care co-ordinators attending (range in teams from 48–100%). Using the Training Acceptability Rating Scale post-training, we found relatively high levels of satisfaction (median overall TARS scores = 56/63; median TARS acceptability scores = 34/36) and perceived effect (median TARS perceived impact score = 22/27).

In terms of opportunities to use the training in routine contacts with patients, data from patient self-report suggested that 79% of patients providing data saw their community mental health team during the six month follow up, with a mean of 12.3 contacts.

### Outcome data—Primary analysis

107 patients were lost to follow up (see CONSORT Fig 1), resulting in 497 patients available for analysis. The pattern of missing data was assessed in terms of baseline characteristics of service users to check for differential non-response. Predictors of non-response were included as covariates in each model to satisfy the ‘missing at random’ assumption of maximum likelihood used in estimating linear mixed models. Missing baseline data for the cohort sample were cluster mean imputed [50].

Baseline and follow up data on outcome measures for the cluster cohort for the primary outcome (HCCQ-10) using intention to treat analyses are reported in Table 3 (adjusted mean difference and 95% confidence interval). Results show no statistically significant difference in HCCQ scores between the intervention and usual care at 6 months. The intra cluster correlation coefficient describes the proporiton of variation in outcome due to differences between teams. The ICC for the primary analysis indicates that only 2% of the variation of HCCQ at 6 months was between teams. This ICC is smaller than the study was powered for (ICC = 0.05).

**Table 3:** Primary outcome measure (intention to treat analysis).

	Usual care			Intervention			Adjusted* mean difference	95% CI	P value	ICC	
Cluster cohort (primary analysis)*	Mean	SD	n	Mean	SD	n					
Baseline HCCQ10 (range 1–7)	5.06	1.66	272	5.27	1.48	332					
6 months HCCQ10	4.93	1.78	227	5.01	1.70	269	-0.064	(-0.343, 0.215)	0.654	0.02	
Cluster cross section (secondary analysis) ^#^											
Baseline HCCQ10	N/A	N/A	N/A	N/A	N/A	N/A					
6 months HCCQ10	5.08	1.72	287	5.09	1.71	341	-0.080	(-0.462, 0.303)	0.683	0.05	
Combined (secondary analysis) ^†^										Cohort	Cross section
Baseline HCCQ10	5.06	1.66	272	5.27	1.48	332					
6 months HCCQ10	5.02	1.75	514	5.06	1.71	610	-0.085	(-0.398, 0.228)	0.595	0.05	0.06

^*^ Controlling for baseline HCCQ10, age, ethnicity, gender, time using NHS services and time experiencing mental health problems

^#^ Controlling for age, ethnicity, gender and time using NHS services

^†^ Controlling for baseline HCCQ10 (using the missing indicator method), age, ethnicity, gender, time using NHS services, variables unbalanced between the cohort and cross-sectional samples: living arrangements and employment status.

Model fit was assessed using diagnostic plots of the model residuals. A histogram of the residuals suggested normality, while plots of the standardised residuals against covariates indicated no correlations.

### Outcome data—Secondary analysis

The results of the cluster cross section and combined analyses were similar to the primary analysis, with no statistically significant difference on the primary outcome between the intervention and usual care at six months (Table 3).

Analyses of secondary outcomes in the cluster cohort are presented in Table 4, which found a significant effect on a single outcome of service satisfaction. All other analyses are presented in the S1 Table.

**Table 4:** Secondary outcomes in the cluster cohort (intention to treat analysis).

		Control			Intervention			Adjusted mean difference (Int–Control)	95% CI	P value	ICC
		Mean	SD	n	Mean	SD	n				
EQUIP PROM*	Baseline	22.82	8.76	272	22.08	8.60	332				
(range 0–44)	6 months	21.62	11.15	153	21.34	9.64	192	0.416	(-1.817, 2.648)	0.715	0.05
HADS-A	Baseline	11.35	5.36	272	12.25	5.19	332				
(Anxiety)^+^ (range 0–21)	6 months	10.86	5.85	172	12.10	5.38	208	0.373	(-0.391, 1.136)	0.339	0.00
HADS-D	Baseline	9.18	5.30	272	10.05	5.19	332				
(Depression)^+^ (range 0–21)	6 months	8.90	5.81	172	9.82	5.50	208	-0.020	(-0.880, 0.839)	0.963	0.00
VSSS-54^+^ (range 1–5)	Baseline	3.58	0.62	272	3.53	0.61	332				
	6 months	3.53	0.80	156	3.52	0.72	191	0.121	(0.002, 0.240)	0.045	0.01
CALPAS^+^ (range 12–84)	Baseline	4.98	1.27	272	5.06	1.19	332				
	6 months	4.86	1.45	152	4.82	1.38	191	-0.008	(-0.245, 0.262)	0.949	0.01
GASS^#^ (range 0–66)	Baseline	17.75	10.36	272	18.30	8.94	332				
	6 months	17.81	11.52	115	19.81	10.32	143	1.297	(-1.071, 3.666)	0.283	0.05
WHOQOL* (range 1–5)	Baseline	3.03	1.01	272	3.05	1.05	332				
	6 months	3.20	1.18	158	3.16	1.11	200	0.027	(-0.167, 0.221)	0.784	0.00
DREEM* (range 24–120)	Baseline	41.71	9.59	272	42.11	9.27	332				
	6 months	42.63	12.45	118	43.54	13.26	142	-0.017	(-2.701, 2.666)	0.990	0.01
WEMWBS^+^ (range 14–70)	Baseline	39.22	12.30	272	38.80	11.89	332				
	6 months	41.09	13.74	162	38.80	13.31	203	-0.671	(-2.599, 1.256)	0.495	0.00

*Controlling for baseline outcome, ethnicity, gender, time using NHS services and accommodation type

^+^Controlling for baseline outcome, ethnicity, gender, time using NHS services, accommodation type and age

^#^Controlling for baseline outcome, ethnicity, gender and time using NHS services

## Discussion

### Statement of principal findings

An intervention to embed shared decision-making in routine practice by improving patient and carer involvement in care planning in community mental health services was well attended and acceptable to staff, but had no significant effects on patient perceptions of autonomy support, or other outcomes.

### Strengths and weaknesses of the study

Our study achieved the required sample size in both the cluster cohort and cluster cross section. Our adoption of cohort and cross-sectional samples provided protection from potential attrition bias, although in practice we achieved high follow up in both groups. Our comprehensive outcome assessment increases confidence that the lack of effect is robust.

Our pragmatic study was designed to maximise uptake among routine community mental health services. We intervened in 18 sites with over 300 care coordinators. However, our volunteer sites may not be fully representative of the wider population, and we lacked data by which to compare our participants with the eligible population. A known risk to cluster trial validity is professionals recruiting differently depending on allocation. To reduce risk, we selected patients via existing registers and invited patients before revealing allocation to teams. Practitioners could potentially exclude patients after invitation (e.g., if they considered the service user to be too unwell to participate), although this involved only a small proportion of patients (n = 6 or 1.25% of the 480 excluded after receiving consent to contact, 4 control and 2 intervention). Measures of service use and contact with professionals were based on self-report, and such measures may not always agree with other sources, such as service records. We offered patients the option of completing follow up assessments face-to-face or by telephone, as this potentially reduces bias if it leads to less attrition. It is possible that telephone adminstration might impact on patient responses, although this would be balanced over the arms of the trial.

### Comparison with other studies

Significantly, our results concur with a growing body of evidence that highlights the challenges associated with embedding shared decision-making in routine services [2,15, 51]. Although some individual interventions and decision aids have shown an impact, these are often in selected clinical situations,^3^ involving decisions about discrete treatments, and it is not clear whether their effects can be replicated outside the trial context. To make shared decision-making a routine part of mental health care, the challenge is to achieve effectiveness while simultaneously maximising reach.

Meaning of the study: possible mechanisms and implications for clinicians and policy makers Our failure to demonstrate benefit requires explanation. We evaluated an intervention derived from published evidence, specifically designed to overcome known barriers to shared decision-making. MAGIC identified a lack of suitable patient reported measures for shared decision-making. Our measure was validated, but the challenges of measurement should not be underestimated, especially among patients with limited previous experience of shared decision-making.

Understanding causal pathways in cluster trials requires an assessment of fidelity, which in the context of a training intervention means exploring processes involving the clusters (i.e., the delivery of training and supervision to the teams) and patients (which patients received the intervention, and what behaviour change occurred in those consultations?) [52]. At the cluster level, we achieved good staff attendance, but there were important limits. We stipulated an attendance rate of 80% of care co-coordinators per team, ten of which demonstrated 80% or greater attendance (range 48–100%). However, no psychiatrists attended, which may have limited the impact of our intervention.

We did not have data on actual delivery of the skills taught in the training (for example, from observations or recordings of care planning discussions after the training). Data from the nested process evaluation incorporating semi-structured interviews with 54 patients, carers and professionals (conducted at baseline, 6 and 12 month post intervention) demonstrated that professionals may not have had sufficient opportunity to use skills derived from the intervention. This data demonstrated that the current operationalisation and utilisation of care planning within mental health services was a significant barrier to involving patients and carers in the care planning process [53]. Such organisational constraints and a lack of consideration of the relational work required to undertake shared decision-making in mental health services meant that despite ideological buy-in from professionals they were not able to routinely embed practices into local service provision.

MAGIC emphasised the importance of senior management demonstrating visible support for shared decision-making. Taking part in our trial required some institutional *agreement* from our host health organisations, although we did not require significant institutional *commitment* (such as investment of resources) beyond staff time. Effective interventions may require more comprehensive commitment, including explicit validation of time spent with patients, endorsement of shared decision-making through clinical leadership and incentives, and fostering new ways to meet patient needs within the constraints of available resources. Such comprehensive change will necessarily increase costs and limit delivery at scale. Historically, the majority of patients and carers have felt marginalised in care planning decisions [21]. The potential for short-term interventions such as the one tested here to impact on entrenched attitudes may be limited.

### Unanswered questions and future research

There remains an urgent need to develop ways of improving shared decision-making at scale. More complex, comprehensive and enduring interventions may be required, such as the use of incentives, adoption of formal decision aids, linkage to routine outcome monitoring, and better integration into routine clinical systems [27]. However, such comprehensive models raise significant challenges for their implementation (and their assessment in a rigorous, controlled fashion).

## Conclusions

Our trial suggests that embedding shared decision-making into mental health care planning cannot be achieved within routine services, at least not to a level which generates demonstrable changes for patients. Enhancing shared decision-making may require considerably greater investment of resources and effects may only be apparent over the longer term.

### Data sharing statement

Copies of the training materials, statistical code, and anonymised data are available from the corresponding author on reasonable request.

## Supporting information

S1 CONSORT checklist (PDF)

S1 Table (DOCX)

S1 Protocol (PDF)

S2 Protocol (PDF)

## References

[1] World Health Organization. Comprehensive Mental Health Action Plan 2013–2020. 2013;Available at: http://www.who.int/mental_health/publications/action_plan/en/ [Accessed 22nd November 2017]

[2] Elwyn G, Coulter A, Laitner S, Walker E, Watson P, Thomson R. Implementing shared decision making in the NHS. *BMJ*. 2010;341:c5146 10.1136/bmj.c5146 20947577

[3] Stacey D, Légaré F, Lewis K, Barry MJ, Bennett CL, Eden KB, et al Decision aids for people facing health treatment or screening decisions. *Cochrane Database Syst Rev*. 2017;Issue 4:CD001431. 10.1002/14651858.CD001431.pub5 PMC6478132 28402085

[4] Slade M. Implementing shared decision making in routine mental health care. *World Psychiatry*. 2017;16:146–153 10.1002/wps.20412 28498575 PMC5428178

[5] Department of Health. Effective care co-ordination in mental health services: modernising the care programme approach—A policy booklet. London: 1999

[6] Department of Health. Refocusing the care programme approach. Policy and Positive Practice Guidance. London: 2008

[7] Freidl M, Pesola F, Konrad J, Puschner B, Kovacs AI, De Rosa C, et al Effects of clinical decision topic on patients’ involvement in and satisfaction with decisions and their subsequent implementation. *Psychiatr Serv*. 2016;67(6):658–663 10.1176/appi.ps.201500083 26876660

[8] HM Government. No health without mental health: a cross government mental health outcomes strategy for people of all ages. London: 2011

[9] Priebe S. Common sense alone is not enough. *World Psychiatry*. 2017;16:157–158 10.1002/wps.20416 28498579 PMC5428176

[10] Elwyn G, Legare F, van der Weijden T, Edwards A, May C. Arduous implementation: does the normalisation process model explain why it’s so difficult to embed decision support technologies for patients in routine practice? *Implement Sci*. 2008;31(3):57 10.1186/1748-5908-3-57 PMC2631595 19117509

[11] Care Quality Commission. Survey of mental health inpatient services. London: 2009

[12] Healthcare Commission. Community mental health service users’ survey. London: 2008

[13] Mental Health Taskforce. The five year forward view for mental health. 2016;Available at: https://www.england.nhs.uk/wp-content/uploads/2016/02/Mental-Health-Taskforce-FYFV-final.pdf [Accessed: 31 October 2017]

[14] Duncan E, Best C, Hagen S. Shared decision making interventions for people with mental health conditions. *Cochrane Database of Systematic Reviews*. 2010; Issue 1. Article No:CD007297 10.1002/14651858.CD007297.pub2 PMC7209977 20091628

[15] Stovell D, Morrison AP, Panayiotou M, Hutton P. Shared treatment decision-making and empowerment-related outcomes in psychosis: systematic review and meta-analysis. *The British Journal of Psychiatry*. 2016; 209 (1) 23–28 10.1192/bjp.bp.114.158931 27198483

[16] Michaelis S, Kriston L, Härter M, Watzke B, Schulz H, Melchior H. Predicting the preferences for involvement in medical decision making among patients with mental disorders. *PLoS ONE*. 2017;12(8):e0182203 10.1371/journal.pone.0182203 28837621 PMC5570317

[17] Joseph-Williams N, Lloyd A, Edwards A, Stobbart L, Tomson D, Macphail S, et al Implementing shared decision making in the NHS: lessons from the MAGIC programme. *BMJ*. 2017;357:j1744 10.1136/bmj.j1744 28420639 PMC6284240

[18] Rogers A, Day JC, Williams B, Randall F, Wood P, Healy D, et al The meaning and management of neuroleptic medication: a study of patients with a diagnosis of schizophrenia. *Soc Sci Med*. 1998;47(9):1313–23 9783874 10.1016/s0277-9536(98)00209-3

[19] Corrigan PW, Watson AC. Understanding the impact of stigma on people with mental illness. *World Psychiatry*. 2002;1(1):16–20 16946807 PMC1489832

[20] Henderson C, Evans-Lacko S, Thornicroft G. Mental Illness Stigma, Help Seeking, and Public Health Programs. *Am J Public Health*. 2013;103(5):777–780 10.2105/AJPH.2012.301056 23488489 PMC3698814

[21] Bee P, Price O, Baker J, Lovell K. Systematic synthesis of barriers and facilitators to service user-led care planning. *Br J Psychiatry*. 2015;207(2):104–114 10.1192/bjp.bp.114.152447 26243762 PMC4523927

[22] Bower P, Roberts C, O’Leary N, Callaghan P, Bee P, Fraser C, et al A cluster randomised controlled trial and process evaluation of a training programme for mental health professionals to enhance user involvement in care planning in service users with severe mental health issues (EQUIP): Study protocol for a randomised controlled trial. *Trials*. 2015;16:348 10.1186/s13063-015-0896-6 26268221 PMC4535374

[23] Loudon K, Treweek S, Sullivan F, Donnan P, Thorpe KE, Zwarenstein M, et al The PRECIS-2 tool: designing trials that are fit for purpose. *BMJ*. 2015;350:h2147 10.1136/bmj.h2147 25956159

[24] Elwyn G, Frosch D, Volandes AE, Edwards A, Montori VM. Investing in deliberation: a definition and classification of decision support interventions for people facing difficult health decisions. *Med Decis Making*. 2010;30(6):701–11 10.1177/0272989X10386231 21088131

[25] Protheroe J, Brooks H, Chew-Graham C, Gardner C, Rogers A. 'Permission to participate?' A qualitative study of participation in patients from differing socio-economic backgrounds. *J Health Psychol*. 2013;18(8):1046–55 10.1177/1359105312459876 23104997

[26] Montori VM, Kunneman M, Brito JP. Shared decision making and improving health care: the answer is not in. *J Am Med Assoc*. 2017;318(7):617–618 10.1001/jama.2017.10168 28810005

[27] Coulter A. Shared decision making: everyone wants it, so why isn't it happening?. *World Psychiatry*. 2017;16:117–118 10.1002/wps.20407 28498596 PMC5428189

[28] Grundy A, Bee P, Meade O, Callaghan P, Beatty S, Olleveant N, et al Bringing meaning to user involvement in mental health care planning: A qualitative exploration of service user perspectives. *J Psychiatr Ment Health Nurs*. 2016;23:12–21 10.1111/jpm.12275 26634415

[29] Cree L, Brooks H, Berzins K, Fraser C, Lovell K, Bee P. Carers’ experiences of involvement in care planning: A qualitative exploration of the facilitators and barriers to engagement with mental health services. *BMC Psychiatry*. 2015;15:208 10.1186/s12888-015-0590-y 26319602 PMC4553006

[30] Bee P, Brooks H, Fraser C, Lovell K. Professional perspectives on service user and carer involvement in mental health care planning: A qualitative study. *Int J Nurs Stud*. 2015;52:1834–1845 10.1016/j.ijnurstu.2015.07.008 26253574 PMC4642654

[31] Brooks H, Sanders C, Lovell K, Fraser C, Rogers A. Re-inventing care planning in mental health: Stakeholder accounts of the imagined implementation of a user/carer involved intervention. *BMC Health Serv Res*. 2015;15:490 10.1186/s12913-015-1154-z 26519298 PMC4628327

[32] Fraser C, Grundy A, Meade O, Callaghan P, Lovell K. EQUIP training the trainers: an evaluation of a training programme for service users and carers involved in training mental health professionals in user-involved care planning. *J Psychiatr Ment Health Nurs*. 2017;24:367–376 10.1111/jpm.12361 28105690

[33] Davis JR, Rawana EP, Capponi DR. Acceptability of behavioural staff management techniques. *Behavioral Residential Treatment*. 1989;4:23–44

[34] Milne D, Noone S. Teaching and Training for Non-Teachers. The British Psychological Society. Leicester: 1996

[35] Grundy A, Walker L, Meade O, Fraser C, Cree L, Bee P, et al Evaluation of a co-delivered training package for community mental health professionals on service user and carer involved care planning. *J Psychiatr Ment Health Nurs*. 2017;24:358–366 10.1111/jpm.12378 28218977 PMC5574013

[36] Ludman EJ, Simon GE, Rutter CM, Bauer MS, Unutzer J. A measure for assessing patient perception of provider support for self-management of bipolar disorder. *Bipolar Disord*. 2002;4:249–53 12190714 10.1034/j.1399-5618.2002.01200.x

[37] Williams GC, Lynch MF, McGregor HA, Ryan RM, Sharp D, Deci EL. Validation of the "Important Other" Climate Questionnaire: Assessing Autonomy Support for Health-Related *Change Fam Syst Health*. 2006;24(2):179–194

[38] Bee P, Gibbons C, Callaghan P, Fraser C, Lovell K. Evaluating and quantifying user and carer involvement in mental health care planning (EQUIP): Co-development of a new patient-reported outcome measure. *PLoS ONE*. 2016;11(3):e0149973 10.1371/journal.pone.0149973 26963252 PMC4786101

[39] Ruggeri M, Lasalvia A, Dall’agnola R, Wijngaarden B, Knudsen HC, Leese M, et al Development, internal consistency and reliability of the Verona Service Satisfaction Scale–European Version–EPSILON Study 7. *Br J Psychiatry* Suppl. 2000;177:S41–8 10.1192/bjp.177.39.s41 10945077

[40] Ruggeri M, Dall’agnola R. The development and use of the Verona expectations for care scale (VECS) and the Verona service satisfaction scale (VSSS) for measuring expectations and satisfaction with community-based psychiatric-services in service users, relatives and professionals. *Psychol Med*. 1993;23(2):511–23 8332665 10.1017/s0033291700028609

[41] Waddell L, Taylor M. A new self-rating scale for detecting atypical or second-generation antipsychotic side effects. *J Psychopharmacol*. 2008;22(3):238–43 10.1177/0269881107087976 18541624

[42] Tennant R, Hiller L, Fishwick R, Platt S, Joseph S, Weich S, et al The Warwick-Edinburgh mental well-being scale (WEMWBS): development and UK validation. *Health Qual Life Outcomes*. 2007;5:63 10.1186/1477-7525-5-63 18042300 PMC2222612

[43] Ridgway PA, Press A. Assessing the recovery commitment of your mental health services: a user’s guide for the Developing Recovery Enhancing Environments Measure (DREEM). Recovery Devon; 2004

[44] Zigmond AS, Snaith RP. The Hospital Anxiety and Depression Scale. *Acta Psychiatr Scand*. 1983;67(6):361–70 6880820 10.1111/j.1600-0447.1983.tb09716.x

[45] Gaston L. Reliability and criterion-related validity of the California Psychotherapy Alliance Scales (CALPAS)–patient version. *Psychol Assess*. 1991;3(1):68

[46] Skevington SM, Lotfy M, O'Connell KA. The World Health Organization's WHOQOL-BREF quality of life assessment: psychometric properties and results of the international field trial—A report from the WHOQOL group. *Qual Life Res*. 2004;13(2):299–310 10.1023/B:QURE.0000018486.91360.00 15085902

[47] Lelliott P, Beevor A, Hogman G, Hyslop J, Lathlean J, Ward M. Carers’ and users’ expectations of services–carer version (CUES-C): a new instrument to support the assessment of carers of people with a severe mental illness. *J Ment Health*. 2003;12(2):143–52

[48] Janssen MF, Pickard AS, Golicki D, Gudex C, Niewada M, Scalone L, et al Measurement properties of the EQ-5D-5L compared to the EQ-5D-3L across eight patient groups: a multi-country study. *Qual Life Res*. 2013;22(7):1717–27 10.1007/s11136-012-0322-4 23184421 PMC3764313

[49] StataCorp. 2013 *Stata Statistical Software*: *Release 13*. College Station, TX: StataCorp LP

[50] White IR, Thompson SG. Adjusting for partially missing baseline measurements in randomized trials. *Statistics in Medicine*. 2005;24(7): 993–1007 10.1002/sim.1981 15570623

[51] Kennedy A, Bower P, Reeves D, Blakeman T, Bowen R, Chew-Graham C, et al Implementation of self-management support for long term conditions in routine primary care settings: cluster randomised controlled trial. *BMJ*. 2013;346:f2882 10.1136/bmj.f2882 23670660 PMC3652644

[52] Grant A, Treweek S, Dreischulte T, Foy R, Guthrie B. Process evaluations for cluster-randomised trials of complex interventions: a proposed framework for design and reporting. *Trials*. 2013;14:15 10.1186/1745-6215-14-15 23311722 PMC3600672

[53] Brooks HL, Lovell K, Bee P, Sanders C, Rogers A. Is it time to abandon care planning in mental health services? A qualitative study exploring the views of professionals, service users and carers. *Health Expect*. 2017;00:1–9 10.1111/hex.12650 PMC5980609 29144591

