# Supplementary material for: Embedding shared decision-making in the care of patients with severe and enduring mental health problems: The EQUIP pragmatic cluster randomised trial
Source: PLoS One. 2018 Aug 22;13(8):e0201533. doi: 10.1371/journal.pone.0201533 (PMC6104914; doi:10.1371/journal.pone.0201533)
Supplement: S1 Table — (DOCX) [file pone.0201533.s002.docx]

Cross-sectional sample demographics

|  | | Cross-sectional | | | | | | | |
| --- | --- | --- | --- | --- | --- | --- | --- | --- | --- |
|  | | Control  (n=312) | | | | Intervention  (n=370) | | | |
|  | | n | | % | | n | | % | |
| Age | 18-24 | 20 | | 6.41 | | 24 | | 6.49 | |
|  | 25-44 | 131 | | 41.99 | | 142 | | 38.38 | |
|  | 45-64 | 134 | | 42.95 | | 166 | | 44.86 | |
|  | 65+ | 24 | | 7.69 | | 32 | | 8.65 | |
|  | Missing | 3 | | 0.96 | | 6 | | 1.62 | |
| Gender | Female | 172 | | 55.13 | | 225 | | 60.81 | |
|  | Male  Other | 133  1 | | 42.63  0.32 | | 137  1 | | 37.03  0.27 | |
|  | Missing | 6 | | 1.92 | | 7 | | 1.89 | |
| Ethnic group | White | 278 | | 89.10 | | 311 | | 84.05 | |
|  | Non-White | 28 | | 8.97 | | 45 | | 12.16 | |
|  | Missing | 6 | | 1.92 | | 14 | | 3.78 | |
| Education | Secondary school | 148 | | 47.44 | | 166 | | 44.86 | |
|  | Higher education | 114 | | 36.54 | | 149 | | 40.27 | |
|  | Missing | 50 | | 16.03 | | 55 | | 14.86 | |
| Accommodation | Owner occupier | 86 | | 27.56 | | 111 | | 30.00 | |
|  | Other | 218 | | 69.87 | | 245 | | 66.22 | |
|  | Missing | 8 | | 2.56 | | 14 | | 3.87 | |
| Living arrangements | Alone or with a pet | 165 | | 52.88 | | 196 | | 52.97 | |
|  | With someone else | 144 | | 46.15 | | 168 | | 45.41 | |
|  | Missing | 3 | | 0.96 | | 6 | | 1.62 | |
| Employment | Employed | 40 | | 12.82 | | 55 | | 14.86 | |
|  | Other | 268 | | 85.90 | | 303 | | 81.89 | |
|  | Missing | 4 | | 1.28 | | 12 | | 3.24 | |
|  | | Median | IQR | | n | Median | IQR | | n |
| Time experiencing mental health problems (months) | | 194.5 | 108 – 324 | | 288 | 185 | 97.5 – 324 | | 340 |
| Time using NHS services (months) | | 120 | 52 – 324 | | 284 | 120 | 40 – 240 | | 338 |

Cross-sectional sample results

| Outcome |  | Control | | | Intervention | | | Adjusted mean difference (Intervention –Control) | 95% Confidence Interval | P value | ICC |
| --- | --- | --- | --- | --- | --- | --- | --- | --- | --- | --- | --- |
|  |  | Mean | SD | n | Mean | SD | n |  |  |  |  |
|  |  |  |  |  |  |  |  |  |  |  |  |
| HCCQ-10^*^ | 6 months | 5.08 | 1.72 | 287 | 5.09 | 1.71 | 341 | -0.080 | (-0.462, 0.303) | 0.683 | 0.05 |
| EQUIP PROM^#^ | 6 months | 25.18 | 13.56 | 245 | 25.68 | 13.48 | 306 | -0.342 | (-3.689, 3.005) | 0.841 | 0.07 |

^*^ Controlling for gender, ethnicity, age and time using NHS services
^#^ Controlling for gender, ethnicity, time using NHS services and education.

Carer demographics

|  | | Cohort | | | |
| --- | --- | --- | --- | --- | --- |
|  | | Control  (n=44 ) | | Intervention  (n=46) | |
|  | | n | % | n | % |
| Gender | Female | 22 | 50.00 | 25 | 54.35 |
|  | Male | 22 | 50.00 | 20 | 43.48 |
|  | Missing | 0 | 0.00 | 1 | 2.17 |
| Ethnic group | White | 39 | 88.64 | 40 | 86.96 |
|  | Non-White | 5 | 11.36 | 6 | 13.04 |
|  | Missing | 0 | 0.00 | 0 | 0.00 |
| Education | Secondary school | 17 | 38.64 | 20 | 43.48 |
|  | Higher education | 22 | 50.00 | 23 | 50.00 |
|  | Missing | 5 | 11.36 | 3 | 6.52 |
| Accommodation | Owner occupier | 25 | 56.82 | 32 | 69.57 |
|  | Other | 19 | 43.18 | 14 | 30.43 |
|  | Missing | 0 | 0.00 | 0 | 0.00 |
| Living arrangements | Alone or with a pet | 13 | 29.55 | 14 | 30.43 |
|  | With someone else | 31 | 70.45 | 31 | 67.39 |
|  | Missing | 0 | 0.00 | 1 | 2.17 |
| Employment | Employed | 15 | 34.09 | 23 | 50.00 |
|  | Other | 29 | 65.91 | 22 | 47.83 |
|  | Missing | 31 | 70.45 | 1 | 2.17 |

Carer results – All information (intention to treat analysis)

|  |  | Control | | | Intervention | | | Adjusted* mean difference (Intervention – Control) | 95% Confidence Interval | P value | ICC |
| --- | --- | --- | --- | --- | --- | --- | --- | --- | --- | --- | --- |
|  |  | Mean | SD | n | Mean | SD | n |  |  |  |  |
| PROM-14^*^ | Baseline | 19.48 | 10.96 | 44 | 20.97 | 12.64 | 20 |  |  |  |  |
|  | 6 months | 16.45 | 10.86 | 46 | 20.10 | 8.00 | 22 | 0.392 | (-5.676, 6.460) | 0.899 | 0.00 |
| WHOQOL^#^ | Baseline | 3.45 | 0.90 | 44 | 3.69 | 0.81 | 46 |  |  |  |  |
|  | 6 months | 3.27 | 1.15 | 26 | 3.91 | 1.00 | 23 | 0.484 | (0.009, 0.959) | 0.046 | 0.00 |
| CUES-C^†^ | Baseline | 24.68 | 8.02 | 44 | 24.67 | 8.28 | 46 |  |  |  |  |
|  | 6 months | 24.12 | 9.97 | 26 | 22.71 | 9.08 | 24 | -0.972 | (-4.438, 2.440) | 0.577 | 0.00 |

^*^PROM-14 adjusted for baseline PROM, gender and ethnicity
^#^WHOQOL adjusted for baseline WHOQOL, gender and ethnicity
^†^CUES-C Adjusted for baseline CUES-C, gender, ethnicity and education

Carer results – model estimates

| Measure | Adjusted mean difference (I-C) | 95% CI | P value | ICC |
| --- | --- | --- | --- | --- |
| PROM-14^*^ | 0.392 | (-5.676, 6.460) | 0.899 | 0.00 |
| WHOQOL^#^ | 0.484 | (0.009, 0.959) | 0.046 | 0.00 |
| CUES-C^†^ | -0.972 | (-4.438, 2.440) | 0.577 | 0.00 |

^*^PROM-14 adjusted for baseline PROM, gender and ethnicity
^#^WHOQOL adjusted for baseline WHOQOL, gender and ethnicity
^†^CUES-C Adjusted for baseline CUES-C, gender, ethnicity and education
