# Supplementary material for: Embedding shared decision-making in the care of patients with severe and enduring mental health problems: The EQUIP pragmatic cluster randomised trial
Source: PLoS One. 2018 Aug 22;13(8):e0201533. doi: 10.1371/journal.pone.0201533 (PMC6104914; doi:10.1371/journal.pone.0201533)
Supplement: S1 Protocol — (PDF) [file pone.0201533.s003.pdf]

Bower et al. Trials (2015) 16:348
DOI 10.1186/s13063-015-0896-6

STUDY PROTOCOL Open Access

A cluster randomised controlled trial and process evaluation of a training programme for mental health professionals to enhance user involvement in care planning in service users with severe mental health issues (EQUIP): study protocol for a randomised controlled trial

Peter Bower[1*], Chris Roberts[3], Neil O'Leary[3], Patrick Callaghan[4], Penny Bee[2], Claire Fraser[2], Chris Gibbons[2], Nicola Olleveant[2], Anne Rogers[5], Linda Davies[6], Richard Drake[2,7], Caroline Sanders[1], Oonagh Meade[4], Andrew Grundy[4], Lauren Walker[2], Lindsey Cree[2], Kathryn Berzins[2], Helen Brooks[2], Susan Beatty[2], Patrick Cahoon[7], Anita Rolfe[7] and Karina Lovell[2]

Abstract

Background: Involving service users in planning their care is at the centre of policy initiatives to improve mental health care quality in England. Whilst users value care planning and want to be more involved in their own care, there is substantial empirical evidence that the majority of users are not fully involved in the care planning process. Our aim is to evaluate the effectiveness and cost-effectiveness of training for mental health professionals in improving user involvement with the care planning processes.

Methods/Design: This is a cluster randomised controlled trial of community mental health teams in NHS Trusts in England allocated either to a training intervention to improve user and carer involvement in care planning or control (no training and care planning as usual).

We will evaluate the effectiveness of the training intervention using a mixed design, including a 'cluster cohort' sample, a 'cluster cross-sectional' sample and process evaluation. Service users will be recruited from the caseloads of care co-ordinators.

The primary outcome will be change in self-reported involvement in care planning as measured by the validated Health Care Climate Questionnaire. Secondary outcomes include involvement in care planning, satisfaction with services, medication side-effects, recovery and hope, mental health symptoms, alliance/engagement, well-being and quality of life. Cost- effectiveness will also be measured. A process evaluation informed by implementation theory will be undertaken to assess the extent to which the training was implemented and to gauge sustainability beyond the time-frame of the trial.

(Continued on next page)

* Correspondence: peter.bower@manchester.ac.uk
[1]NIHR School for Primary Care Research, Manchester Academic Health Science Centre, University of Manchester, Williamson Building, Oxford Road, Manchester M13 9PL, England
Full list of author information is available at the end of the article

© 2015 Bower et al. **Open Access** This article is distributed under the terms of the Creative Commons Attribution 4.0 International License (http://creativecommons.org/licenses/by/4.0/), which permits unrestricted use, distribution, and reproduction in any medium, provided you give appropriate credit to the original author(s) and the source, provide a link to the Creative Commons license, and indicate if changes were made. The Creative Commons Public Domain Dedication waiver (http://creativecommons.org/publicdomain/zero/1.0/) applies to the data made available in this article, unless otherwise stated.

(Continued from previous page)

Discussion: It is hoped that the trial will generate data to inform mental health care policy and practice on care planning.

Trial Registration Number: ISRCTN16488358 (14 May 2014)

Keywords: Care planning, Severe mental illness, Service user involvement

Background

Involving service users in their own care and allowing choice is at the centre of policy initiatives aimed at improving quality of care and enhancing recovery. This principle is enshrined and prioritised in health care [1, 2], particularly mental health care policy and guidelines [3, 4]. Current guidance states that the outcome of any assessment will be a care plan developed with the service user, the professional/member of the care team and other appropriate parties, such as families and carers [5, 6]. Mental health nurses, psychiatrists and allied health and social care workers provide the majority of care for service users regardless of setting. They have a pivotal role in the development of care planning, particularly as care co-ordinators under the Care Programme Approach (CPA) [5, 6]. Despite the consensus among policy-makers, professionals, service users and carers about the importance of involvement there is substantial empirical evidence that the majority of users and carers are marginalised in the care planning process. This lack of involvement occurs in both in-patient and community settings [7, 8] and there is evidence that current models of involving users and carers in their care are less effective than first envisaged.

One of the key findings of the Care Quality Commission's national survey, which included 7500 users of mental health services, was that service users were often not involved in their care as much as they wanted to be, with only 34 % agreeing that they were 'definitely' involved as much as they wanted to be in decisions about their care and treatment [7, 8]. A significant number of studies of user and carer experience echo the need and desire to be involved in their care [9], but despite this evidence there has been little change in practice. There is also evidence that carers feel excluded, unsupported and distanced by mental health services and want to be more involved in the care planning process [10–13]. A systematic review of user and carer views and expectations of mental health nurses, including 132 papers over a 20-year period [14], found that users and carers expressed a need for openness and adequate information, more choice and increased involvement in the assessment and planning of their care. Perhaps most importantly it found that the literature remains strikingly consistent over time, indicating a sustained lack of impact on practice.

This research aims to develop a standardised training intervention (EQUIP training) to improve user and carer involvement with the care planning process, and to test the effectiveness and cost-effectiveness of this package. The study is a pragmatic effectiveness and cost-effectiveness trial and, thus, the optimal comparator is a no training control representing usual practice.

If proven effective and cost-effective, it will provide a model for wider implementation to improve the quality of mental health care across community mental health services.

Objectives

To conduct a cluster randomised controlled trial (RCT) of effectiveness and cost-effectiveness of the EQUIP training for improving involvement of service users and carers compared with controls in UK National Health Service (NHS) community mental health services.

Methods/Design

As the trial involves a health professional training intervention, a cluster randomised design is required to avoid contamination. A mixed design, including a 'cluster cohort' design, a 'cluster cross-sectional' design and process evaluation will be adopted (see Fig. 1 for an outline of the trial design, and Fig. 2 for the Consolidated Standards of Reporting Trials (CONSORT) flow diagram). Adoption of the combined design provides protection against problems in either of the individual approaches.

In the 'cluster cohort' design, service users cared for by each Community Mental Health Team (CMHT) will be recruited, and a detailed face-to-face assessment will be conducted at baseline. Carers will be recruited from consenting service users. Each CMHT will then be randomised to either intervention (training in care planning) or control (usual care planning). The CMHTs randomised to intervention will then receive the EQUIP training. Six-month follow-up face-to-face assessments will then be conducted with the same service users and carers (intervention and control groups) 6 months after the baseline assessment.

For the cluster cohort, service users need to participate in two face-to-face assessments, which may be burdensome to service users. This means the cluster cohort may be vulnerable to recruitment difficulties (i.e., only a small number of eligible service users take part) and attrition (i.e., service users do not attend for follow-up), reducing both sample size and external validity.

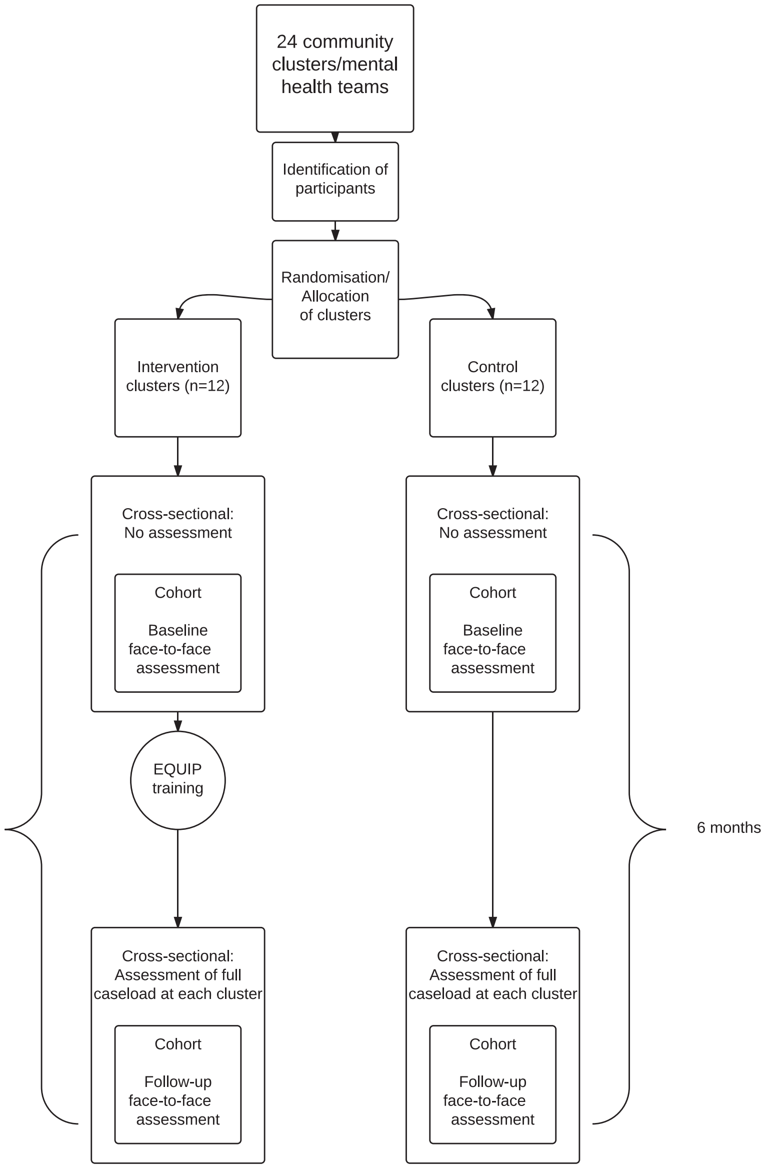

Fig. 1 EQUIP trial design

The design will, therefore, also include a 'cluster cross-sectional' element to help ameliorate problems of recruitment and attrition within the cluster cohort. Six months after randomisation, a smaller number of questionnaires (see Table 1) will be posted to all service users who are not part of the 'cluster cohort' but who are under the care of all participating teams.

Service users in the cross-sectional sample only have to agree to assessment once, and the assessment is designed to be less burdensome as it includes fewer measures. This means that a higher proportion of service users may agree to take part in the study, potentially giving a larger sample size.

However, the 'cluster cohort' design allows adjustment for a wider range of baseline characteristics at an individual level because of the greater range of measures included, giving potentially greater statistical power compared to analysis of the 'cluster cross-sectional' data [15]. Comparative analyses of data from the two designs will allow the possibility of pooling results from the two designs to be investigated.

A potential threat to the validity of a cluster randomised trial is recruitment bias, where professionals allocated to different trial arms recruit differently depending on their allocation, leading to selection bias and baseline incomparability [16]. Whilst it is preferable to recruit

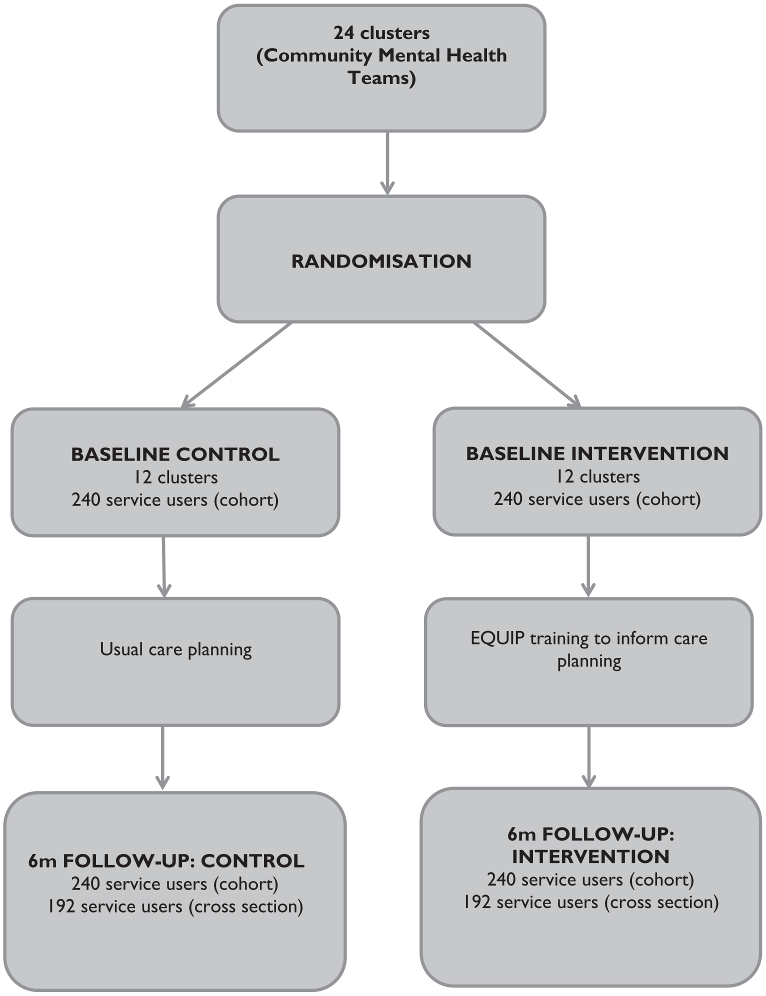

Fig. 2 Consolidated Standards of Reporting Trials (CONSORT) diagram

service users prior to allocation, the logistics of the trial means that clusters will need advanced notice of their training date, which will require us to inform them of their allocation. Therefore, initial patient selection in the EQUIP trial is not by those being trained in the trial, but will use existing registers of service users. This will be undertaken by NIHR Clinical Research Network Clinical Studies Officers (CSO) and Trust staff. This will limit the opportunity for biased recruitment. Carers will be recruited by providing service users with a recruitment pack to give to their carer (if a carer is identified and wishes to consider taking part).

A longitudinal process evaluation of the training programme is being undertaken which incorporates multiple interviews over time, observations and diary methods of data collection. This will run alongside the trial and is being conducted because successful implementation of user and carer-led care planning implicates a range of factors including the integrity of the intervention and the acceptability of the intervention to both clinicians and service users [17].

Study setting and inclusion criteria

Community Mental Health Teams (CMHTs)

The study is being conducted in NHS Trusts in the UK.

All CMHTs within the participating NHS Trusts will be eligible for inclusion. Service users aged 18 and over with a severe mental illness (e.g., psychosis, bipolar disorder, schizophrenia) under the care of participating CMHTs will be eligible for inclusion. We will seek consent from service users to access health records to collect data on diagnosis, service use and treatment history.

Intervention design – User/carer-led training package to inform care planning

All consenting CMHTs allocated to the intervention arm will receive the EQUIP training intervention developed in our earlier work. The EQUIP training intervention

Table 1 Outcome measures used with the 3 sample groups at baseline and 6-month follow-up

Sample	Baseline	Six months
Service user cohort (face-to-face collection)	Demographic data	
	Primary outcome:	Primary outcome:
	HCCQ-10	HCCQ-10
	Secondary outcomes:	Secondary outcomes:
	VSSS-EU-54	VSSS-EU-54
	GASS	GASS
	WEMWBS	WEMWBS
	DREEM	DREEM
	HADS	HADS
	CALPAS-12	CALPAS-12
	WHOQOL-BREF	WHOQOL-BREF
	PROM	PROM
	Economic outcome:	Economic outcome:
	EQ-5D-5L	EQ-5D-5L
	Service use questionnaire	Service use questionnaire
Carer cohort (postal collection)	Demographic data	
	Secondary outcomes:	Secondary outcomes:
	PROM	PROM
	CUES-C	CUES-C
	WHOQOL-BREF	WHOQOL-BREF
	Economic outcome:	Economic outcome:
	EQ-5D-5L	EQ-5D-5L
Cross-sectional sample: service users (postal collection)		Demographic data
		Primary outcome:
		HCCQ-10
		Secondary outcome:
		PROM (14-item SF version)
		Economic outcome:
		EQ-5D-5L
		Service use questionnaire

CALPAS-12 California Psychotherapy Alliance Scales, *CUES-C* Carers' and Users' Expectations of Services – carer version, *DREEM* Developing Recovery Enhancing Environments Measure, *EQ-5D-5L* EuroQol-5D-5L, *GASS* Glasgow Antipsychotic Side-effect Scale, *HADS* Hospital Anxiety and Depression Scale, *HCCQ-10* Health Care Climate Questionnaire, *PROM* Patient Reported Outcome Measure, *WHOQOL-BREF* World Health Organisation Quality of Life., *VSSS-EU-54* Verona Service Satisfaction Scale, *WEMWBS* World Health Organisation Quality of Life

consists of 2 days face-to-face training, an 8-hour optional self-directed learning package and 6 hours supervision per team in the 6 months following the training. In recruitment of teams, we ask that at least 80 % of staff designated as 'care co-ordinators' (i.e., those with a caseload) commit to attend the training. We will document attendance at training by all professionals.

Comparator – Usual care

This will consist of 'usual practice' in care planning, without access to the specialist training described above. We will have considerable detail about what 'usual practice' consists of and how it varies from unit to unit from the embedded process evaluation.

Intervention components

We describe the intervention according to the Template for Intervention Description and Replication (TIDieR) guidelines [18]:

a) Why: our aim was to co-develop, co-produce and co-deliver (with service users/carers) a best evidence, acceptable and feasible training programme for mental health professionals to enhance user and carer involvement in care planning. Two reviews were

conducted including: a narrative synthesis (Bee et al., in press) which examined how user-involved care planning is operationalised within mental health services and to establish where, how and why challenges to user involvement occur; and a scoping review of training reviews and interventions that change clinician behaviour. In addition, focus groups and individual interviews with service users, carers and health professionals were conducted to ascertain training content and delivery requirements and to determine the priorities and components of adequate user and carer involvement in care planning. The evidence from the reviews and qualitative data was synthesised to develop and design the training.

b) What: a range of training materials have been developed for the training including PowerPoint slides, case scenarios, audio-recordings from health professionals, service users and carers and a trainer's manual.

c) Who: the synthesis identified that the training should be multi-disciplinary, including all health professionals and psychiatrists. Team training was seen as optimal and, as far as possible, teams will be trained together. The training will be delivered by 2 of the co-applicants (both academics with teaching experience) and 3/4 service users and carers who have attended a 4-day 'train the trainers' course.

d) How: the synthesis indicated that training should include a range of formats: face-to-face, self-directed learning and follow-up supervision. The consensus exercise indicated a minimum of 15 hours and maximum of 30. The course will run for 2 days (12 hours) plus 6 hours follow-up supervision and 8 hours self-directed learning (optional). Hence, each health professional will receive 18 hours of facilitated training and an additional optional 8 hours self-directed learning.

e) Where: consensus was reached that the training venue should be outside the clinical area, geographically convenient, provide good catering and in a venue with appropriate training resources.

f) When and how: the training will be delivered to each cluster randomised to the training intervention over 2 days. In recruiting teams, we ask that 80 % of the care co-ordinators within each team attend the training. The training will be delivered within 6 weeks of service users being recruited into the trial.

g) Tailoring: the intervention has been tailored for health professionals.

h) Modifications: only minor modifications will be made in light of feedback during the trial. If the trial is successful and we implement the training across other NHS Trusts, modifications will be made in light of feedback collected from the process evaluation.

i) How well: fidelity of the training has been ensured by the careful development and synthesis work described earlier, the 'train the trainers' course, the development of a detailed manual and the delivery of training by the same groups of trainers.

Outcome assessment

Primary outcome

The Health Care Climate Questionnaire (HCCQ-10) [19] is the primary outcome measure for the service users in the trial.

The HCCQ-10 was developed to assess patient experience of health care and the degree to which their care offers support for autonomy. The scale has ten items, which are scored on a seven-point scale ranging from 'strongly disagree' to 'strongly agree'. An overall score is calculated as the mean of the items (expressed out of 100), where a higher score indicates greater support for autonomy.

Secondary outcomes

Secondary outcome measures were determined using experts and a consensus discussion exercise with the service user/carer advisory group. Key domains to measure were recommended by the service user/carer advisory group in our earlier Programme Development Grant (RP-DG-1209-10020). The domains identified were: quality of life; alliance/engagement; service satisfaction; well-being; mental health symptoms; hope and recovery; and medication side-effects. Six of these domains have one questionnaire selected for completion, whilst the domain 'satisfaction' has separate questionnaires for both service users and carers.

A new measure of user involvement in care planning, the Patient Reported Outcome Measure (PROM) was developed in consultation with our user and carer advisory group. The need for this measure was determined during the development of this grant as existing measures of user involvement were not deemed adequate by the advisory group as they failed to sufficiently incorporate the views of users. The newly developed PROM will be included as a secondary outcome to measure user and carer involvement in care planning. The new measure has been validated using the Rasch model and has displayed excellent psychometric and scaling properties [20]. The 61-item scale (and 14-item short form version) is suitable for both service users and carers. Items are scored on a five-point Likert scale from 'Completely disagree' to 'Completely agree'. Higher scores reflect greater service user and carer involvement with care planning. Data from this study will provide further evidence of the

acceptability, validity and sensitivity to change of this measure for this population.

Satisfaction (service users)

Verona Service Satisfaction Scale (VSSS-EU-54) [21, 22], is a validated, multi-dimensional, self-administered scale for measuring service users' satisfaction with mental health services. There are seven dimensions: overall satisfaction, professional skill and behaviour, access, efficacy, types of intervention and relatives' involvement. Participants are asked to express their overall feeling about their experience of the mental health service they have been attending in the last year. Satisfaction ratings are on a five-point Likert scale, with higher scores representing greater satisfaction. Global and subscale scores can be obtained. Reliability testing has shown that the VSSS-EU-54 has good internal consistency and stability.

Satisfaction (carers)

Carers and Users' Expectations of Services - carer version (CUES-C) [23] will be used to measure carers' views of services. There are three parts to the questionnaire; part A measures the impact of caring, part B measures the quality of support provided by carers and part C is a free text response for advice and help. The self-rating scales consist of 13 items each in parts A and B, totalling 26 questions. All questions are answered using a three-point scale. Scores for each part range from 0 to 26, with higher scores representing more dissatisfaction and the need for more support. The scale has been found to be suitable to use to assess carers' experiences.

Medication side-effects

The Glasgow Antipsychotic Side-effect Scale (GASS) [24] is a self-rating scale to detect the side-effects of antipsychotic medication. The scale consists of 22 questions and scores range from 0 to 66. Higher scores reflect more frequent experience of side-effects, with total scores providing three categories of severity (absent/mild side-effects, moderate side-effects and severe side-effects).

Well-being

The Warwick-Edinburgh Mental Well-being Scale (WEMWBS) [25] is a short, psychometrically robust scale, which is easy to complete. It has 14 items scored on a 5-point Likert scale ranging from 'none of the time' to 'all of the time' based on experience over the past 2 weeks. Scores range from 14–70 and a higher score indicates a higher level of mental well-being.

Recovery and hope

Developing Recovery Enhancing Environments Measure (DREEM) [26] is a self-report measure used to assess mental health recovery of people who receive mental health services. It is a 166-item questionnaire which is organised into 24 subscales (such as 'stage of recovery' and 'elements of recovery'), including a final section consisting of open-ended questions. Responses are scored on a five-point Likert scale ranging from 'strongly agree' to 'strongly disagree', with low scores representing more positive experience.

Symptoms

Hospital Anxiety and Depression Scale (HADS) [27] is a 14-item scale using a 4-point Likert scale. Items are added to give two scores, one for anxiety and one for depression, with higher scores representing more severe symptoms. Scores range from 0 to 21 for both anxiety and depression. This is a well-used and validated measure.

Alliance/engagement

California Psychotherapy Alliance Scale (CALPAS) [28] is a 12-item, self-report questionnaire, which provides a total score. It has four subscales: 'service user capacity to work purposefully in therapy', 'the affective bond with the therapist', 'therapist's empathic understanding' and 'involvement and the agreement between the patient and therapist on the goals and tasks of treatment'. Each item is rated on a 6-point Likert scale, with scores ranging from 12 to 84, with higher scores representing better alliance. It has good reliability and validity.

Quality of life

World Health Organisation Quality of Life (WHOQOL-BREF) [29] is a 26-item questionnaire consisting of 4 domains (physical, psychological, social relationships and environment). Each question uses a 5-point Likert scale, ranging from a score of 1 to 5, with higher scores representing more positive ratings. Total scores are computed within each domain. It has been shown to demonstrate good reliability and validity.

Economic outcome

Health Status

The EQ-5D-5L [30] will be used to assess health status and to estimate Quality-Adjusted Life Years (QALYs), using published EQ-5D utility weights for the UK. QALYs will be the primary measure of health benefit for the economic analysis. The EQ-5D-5L, has two parts; part 1, a 5-item questionnaire consisting of 5 dimensions (mobility, self-care, usual activities, pain/discomfort and anxiety/depression). Each dimension has five levels, ranging from no problems to severe problems. The five dimensions can be combined to describe the respondents' health state. Part 2 is a visual analogue scale (VAS) which records respondents' self-rated health on a vertical

VAS, where the end points are labelled, 'best imaginable health state' and 'worst imaginable health state'.

Service use questionnaire

A Service Use Questionnaire will be used at baseline and follow-up to identify the range of services used by each trial participant and how much they have used each service. The measure will be used to estimate the costs of health and social care service use.

Use of outcome measures and methods of data collection

Use of outcome measures with the three sample groups (service user cohort, carer cohort, cross-sectional cohort) is further detailed in Table 1.

Service user cohort sample

Demographic data, primary, secondary and economic outcome measures will be collected via a face-to-face assessment. As the 'cluster cohort' assessment is more burdensome, service users will receive a £10 voucher for their time after completion of the follow-up interview at 6 months.

Carer cohort sample

Demographic data, primary, secondary and economic outcome measures will be collected via a postal method. Carers will receive a £5 voucher following receipt of the follow-up questionnaires at the 6-month time point.

Cross-sectional sample (service users)

Demographic data, primary, secondary and economic outcome measures will be collected via a postal method at the 6-month time point. There will be no collection of carer information in the cross-sectional part of the study. Respondents can choose to receive a £5 voucher on receipt of the questionnaire by providing postal contact details.

Table 1 indicates the measures to be used, with which respondents, and at what time points.

Sample size

The primary outcome is the Health Care Climate Questionnaire (HCCQ-10), identified by our user consultation group as their preferred outcome measure. However, data on the use of this scale with service users with severe mental illness is limited and so we have used a standardised effect to calculate sample size and power. A trial with 12 clusters per arm and a mean of 20 service users per cluster (total sample size of 480) will provide power greater than 80 % to detect a standardised effect size of 0.4. This assumes an intra-cluster correlation coefficient of 0.05 and an 80 % follow-up rate, giving 384 participants with complete data in the analysis. Power will be increased by inclusion of baseline covariates (see Statistical analysis below).

For the cross-sectional component, the primary outcome remains the HCCQ-10. We aim to recruit at least the same number of participants in each cluster; given that the same clusters will be assessed as the cohort component. As with the cohort component a sample size of 384 in the cross-sectional component will ensure power greater than 80 % in the corresponding analysis. Any additional data gathered should increase power.

Recruitment

Trust managers have agreed that we can recruit CMHTs. To recruit professionals we will use our applicants to champion the study. Teams will be introduced to the trial via a letter of support from the chief executive, and/or via meetings with senior managers. Meetings will also be held with area team managers (and if requested, staff) across both sites to facilitate engagement with and understanding of the trial.

Recruitment and training will be undertaken in sequence, to maximise efficiency in delivery of training to community teams, but also to ensure there is sufficient time to permit the relevant baseline assessments to be undertaken with service users (see below). CMHTs will be randomly allocated to receive the EQUIP training or control.

Recruitment of service users and carers - Cluster cohort

Service users will be excluded if their participation is judged as inappropriate by the CMHTs: for example, if a patient is not deemed to have capacity to provide fully informed consent or is too unwell at the time of recruitment. We will seek to document all exclusions and report them as part of the trial CONSORT diagram.

Any carer (aged 18 years or older) of the service user will be eligible for inclusion in the 'cluster cohort' study if they agree to take part. Consent will be implied by response via completion and return of the baseline questionnaires (and return of the consent to contact form if they wish to be contacted at 6-month follow-up).

To recruit service users in the 'cluster cohort', the direct care team within the CMHTs (cluster) will produce a list of all service users who meet eligibility criteria to participate in the trial, including any reasons for exclusions. This list will be referred to as their 'caseload'. These patient lists will be used by the NIHR Clinical Research Network CSOs to send out an introductory letter, participant information sheet and consent to contact form to each patient, inviting them to take part in the study. Service users will be required to 'opt in' by returning the consent to contact form in a pre-paid envelope to the research team. CSOs will contact non-responders by telephone on one occasion to additionally allow

service users to opt in over the telephone. The research team will then follow-up the consent to contact forms, to answer any further questions, and when a participant is recruited, the researcher will continue with a face-to-face informed consent process. Following signed consent, service users will be invited to complete all baseline measures.

Service users will be asked at the informed consent meeting to nominate a carer to be included in the study and, if they choose to do so, will be provided with a questionnaire pack (including introductory letter, information sheet, questionnaire, pre-paid envelope and consent to contact at 6-month follow-up form).

Service users who consent to take part in the trial will be offered the opportunity to take part in the process evaluation based on a purposive sampling framework to ensure variation in gender, age and randomised group. Potential participants will be provided with an invitation letter, information sheet and consent to contact form during the baseline meeting.

Recruitment of service users – Cluster cross-sectional

To recruit service users in the 'cluster cross-sectional' study, we will conduct a postal survey of all service users under the care of each community mental health team 6 months after randomisation, excluding those already recruited to the 'cluster cohort'.

Sequence generation and allocation concealment

Once service users and carers have been sent a trial invitation pack (following caseload review), the clusters will be allocated randomly to either intervention or control. To eliminate selection bias, allocation will be determined through an external telephone randomisation service at the Clinical Trials Unit of the Manchester Academic Health Science Centre.

Clusters will be submitted to the randomisation service in pairs. Each pair will be from the same site and similarly matched in other characteristics where possible, to reduce imbalance between intervention and control arms. One member of the pair will be allocated to intervention by random selection, the other allocated to control.

Blinding

To reduce detection bias, we will seek to blind researchers undertaking assessments of the quality of care planning to the allocation. We will report the success or otherwise of our attempts at blinding.

Data management

All data will be stored securely in line with local data management arrangements. All questionnaires and other paper records will be stored in secure storage facilities at the University of Manchester and the University of Nottingham. Personal identifiable paper records will be stored separate from anonymised paper records. All electronic records will be pseudo-anonymised using a reference number for each participant and stored on a password protected server at the University of Manchester.

Statistical analysis

Analysis of outcomes will follow intention-to-treat principles: outcome data will be sought and included in the analysis for all service users irrespective of receipt of the intervention or completion of care planning during the time scale of the EQUIP trial.

Standard data checking procedures will be used as part of the data cleaning procedure prior to locking the database and linkage to group allocation. We will then model the pattern of missing data in terms of baseline characteristics of service users and treatment allocation to check for differential non-response. Depending on the patterns of missing data we may at this point choose to use multiple-imputation. Multiple-imputation may also be used in sensitivity analyses for non-ignorable missing data assumptions [31].

For the cluster cohort study the intervention effects for the primary outcome (HCCQ-10) and secondary outcome measures will be estimated using a linear mixed model with a random intercept for CMHTs. The baseline value of the outcome will be used as a covariate together with other covariates pre-specified in the statistical analysis plan. The same statistical modelling procedure will be used for estimation of the intervention effect in the cluster cross-sectional study using a restricted set of covariates. Full detail of covariates for each model will be confirmed in the statistical analysis plan. We will then test for heterogeneity of the treatment effect using the combined cohort and cross-sectional data. If there is no evidence of heterogeneity, we will estimate a pooled treatment effect.

A draft statistical analysis plan for primary and secondary outcomes, including sub-group analyses will be presented to and agreed with the Programme Steering Group prior to the commencement of the data analysis.

Economic analysis

A cost-effectiveness acceptability analysis will be conducted from the perspectives of health and social care providers and service users, the key stakeholders in treatment decisions. The time horizon for the primary economic analysis will be at scheduled follow-up (6 months). Data on service use and health status (EQ-5D-5L) for the economic analysis will be collected for all participants at baseline and follow-up and via the cross-sectional survey. The economic analysis will

control for key covariates that are associated with either costs or QALYs. Bootstrap techniques will be used to estimate the cost-effectiveness acceptability curve, likelihood that the intervention could be cost-effective and net benefit statistic.

The objective of the economic study will be to assess the relative cost-effectiveness of the training intervention to improve patient outcomes and/or reduce the costs of health and social care.

Process evaluation

A process evaluation of the training programme delivered as part of the trial is deemed appropriate because successful implementation of the user/carer-led care planning training implicates a range of factors including the integrity of the intervention and the acceptability of the intervention to both clinicians and service users [17].

The process evaluation is designed to explore by how far the user/carer-led care planning has been taken up and implemented in the daily work of the health professionals who attended the training and what the consequences of this uptake have been. It will complement and supplement the evidence provided by the main randomised trial, as recommended by the Medical Research Council (MRC) framework for evaluation of complex intervention [32]. The design and execution of the process evaluation will draw on Normalisation Process Theory (NPT) [33] from the outset as well as other theories of implementation science. NPT comprises four components: coherence (sense making work), cognitive participation (relational work), collective action (operational work), reflexive monitoring (appraisal work). NPT has been developed from empirical studies of the implementation of complex interventions in health care contexts and in relation to mental health contexts in particular. We will focus on: (a) implementation of user/carer-involved care planning – the way this is developed and translated into practices (of mental health professionals, users, carers and others); (b) embedding – the manner in which care planning becomes, (or does not become), routinely incorporated in everyday work of service users and professionals; (c) integration – how care planning is sustained as part of the everyday lives of individuals at work and at home; and d) networking – how it generates access to new networks and resources.

Informed consent

The key ethical concerns for the programme include confidentiality, participant anonymity and informed consent to participate. The study includes both mental health service users and carers as participants and, as such, there are specific ethical issues to be considered. Research governance principles and ethical committee approvals bind all applicants and institutions. We will ensure we adopt the highest standards of research conduct including involvement of service user representation in the management and delivery of the research.

The study will be conducted in compliance with the study protocol, Good Clinical Practice (GCP) and both University and NHS regulatory and monitoring requirements. The Work stream teams will meet every 3 months and the chief investigator (CI) will be responsible for the overall leadership, management and outputs of the programme. The principal investigator (PI) from each site will maintain a log of the key milestones to be achieved against the timetable. The programme managers and Work stream leads will be responsible for the day to day running and co-ordination of the studies and will be accountable to the PI. All research associates will be supervised by the Work stream leads.

All potential participants will be provided with an information sheet written to current NRES guidelines and favourably reviewed by the relevant ethics committee, prior to the study commencing. Service users and carers have been involved in developing the participant information sheets to ensure they are accessible. The information sheet will be provided to potential participants at the point of them expressing an interest in participating. It will provide potential participants with information about the study, including the potential benefits and risks of taking part, confidentiality and the right to withdraw. Researcher contact details will be provided so participants can contact them with any queries prior to the participant deciding whether or not to take part. Researchers will further discuss risks and benefits immediately prior to the data collection taking place.

Participants in randomised trials usually provide written informed consent for a range of research procedures, including participation in the trial, randomisation and data collection. However, conventional informed consent procedures are not always appropriate in the context of a cluster, randomised trial [34]. In the EQUIP trial, community mental health services are making the decision to take part in the EQUIP trial and agree to randomised allocation. This is described as a 'cluster cluster' design, and is distinguished from an 'individual cluster' design [35]. In the latter, randomisation is at the level of the cluster, but specific services are delivered to individuals, and service users can consent to receive or not receive that intervention. The recent CADET trial was an example of an individual cluster design [36]. In 'cluster cluster' designs such as the EQUIP trial, service users cannot opt out of a cluster in the same way, as the community teams will have been trained in the new methods. The recent WISE study was an example of this design [37].

In the EQUIP trial, seeking formal consent for participation and randomisation may, therefore, be inappropriate,

as these processes are not under control of the service users. The following consent procedures will be adopted.

If the service, and the CMHTs consent to take part in the trial, then individual service users will not be asked for specific consent to be randomised as part of the EQUIP trial. Service users cannot, therefore, 'opt out' of their cluster allocation. The Clinical Research Network CSOs (or Trust staff) will be responsible for accessing patient details and determining who is eligible to take part in the study and be contacted. They will be responsible for sending out information about the study to the identified service users, along with an invitation to participate. The research team will not have access to service user details until they have returned the consent to contact form.

Service users in the 'cluster cohort' will undertake a detailed face-to-face assessment at 2 points in time (baseline and 6 months). For these, a formal written consent procedure will be adopted. It will be explained to service users that their CMHT is involved in a study to test the effectiveness of a new training package on service user/carer involvement in care planning compared to the usual care planning experience. Participants will be told that we will do this by delivering the training to some mental health teams and not to other teams to see if receiving the training has an impact on the extent of service user/carer involvement in care planning.

Carers in the 'cluster cohort' will undertake a postal survey at baseline and at 6 months. Carers will be asked to participate via nomination by a service user. Response to the questionnaires will be treated as consent. Carers will also be asked to complete a consent to contact form to allow invitation to complete the follow-up questionnaire at 6 months, which is returnable to the research team in the prepaid envelope provided.

Service users in the 'cross-sectional sample' will undertake a short postal survey at 6 months only. For these, we will treat this part of the study as a survey. Service users will receive a postal invitation to the survey, seeking their views on the quality of the care planning process that they have received. Response to the survey will be treated as consent, as is usual in survey work. Participants may change their mind and withdraw from the study at any point and this will not affect the care they receive.

Ethics

The study received a favourable ethical opinion from the National Research Ethics Service (NRES) on 8 August 2014 (NRES Committee North West Lancaster, REC Reference 14/NW/0297; IRAS Project ID 125899). It will be conducted in accordance with the UK Department of Health Research Governance Framework in Health and Social Care (2005) and adhere to the ethical principles of the Helsinki Declaration (2013). All research staff involved in the conduct of the trial will meet the standards laid out in the ICH Tripartite Guideline for Good Clinical Practice (1996).

All data will be anonymised and secured off site following data input for a period of at least 5 years in accordance with the Data Protection Act (1995).

The design of this trial will not disadvantage service users in either the care planning training groups or control groups. No treatment is being withheld from service users in the control group.

Ancillary studies

Data from an embedded sub-study will contribute to a programme of research (www.population-health.manchester.ac.uk/mrcstart/) funded by the MRC to expand the relatively small evidence base on an important issue concerning the recruitment of participants to trials [38].

It is recognised that patient and public involvement (PPI) is an important part of designing studies that are sensitive to the needs and preferences of patients, and the EQUIP study has invested significant time and resources into ensuring that PPI on the project receives sufficient focus. The embedded sub-study ('START in EQUIP', funded through an NIHR Doctoral Research Fellowship – award number DRF-2012-05-128) aims to evaluate whether telling eligible patients directly about the PPI in EQUIP increases patient recruitment into the study.

Service users will be randomised to receive a flyer detailing the extensive PPI in EQUIP alongside the standard participant information sheet, or the standard participant information sheet alone. This will be implemented by using cluster randomisation, in a crossed-factorial design with the main EQUIP allocation. We will test whether receiving the flyer is associated with higher levels of recruitment into EQUIP.

Discussion

Involving service users in planning their care is at the centre of policy initiatives in the United Kingdom to improve quality of mental health care. Users value care planning and involvement in care, but achieving this in practice is far from straightforward.

Our aim is to evaluate the effectiveness and cost-effectiveness of training for mental health professionals in improving user involvement with the care planning processes. We have designed and delivered a pragmatic trial which will test this training and its impact in the context of routine care delivery. We have adopted very simple inclusion criteria to include as broad a range of patients as possible and enhance the pragmatic nature of the trial.

Conducting trials such as this in the context of routine care raises significant logistical challenges. Recruiting adequate numbers of patients in the limited recruitment window prior to training is proving challenging, and busy services can struggle to provide time for staff to attend the relevant training sessions when faced with significant clinical pressures.

The planned process evaluation will allow us to explore the impact of these pressures on delivery and implementation of the training. If successful in improving outcomes, we will be active in disseminating and implementing the results of the trial.

Trial status

CMHTs in the first 18 clusters have been randomised and training of staff in the experimental arm of these clusters completed. Randomisation and training of the remaining clusters will continue until December 2015. Recruitment and data collection of primary and secondary outcomes with service users and carers began in July 2014 and will continue until June 2016.

Abbreviations

CALPAS: California Psychotherapy Alliance Scales; CI: chief investigator; CMHT: Community Mental Health Team; CONSORT: Consolidated Standards of Reporting Trials; CPA: Care Programme Approach; CSO: Clinical Studies Officers; CUES-C: Carers' and Users' Expectations of Services - carer version; DREEM: Developing Recovery Enhancing Environments Measure; EQUIP: Enhancing the Quality of User Involved Care Planning in Mental Health Services; GASS: Glasgow Antipsychotic Side-effect Scale; GCP: Good Clinical Practice; HADS: Hospital Anxiety and Depression Scale; HCCQ-10: Health Care Climate Questionnaire; ICH: International Conference on Harmonisation; ISRCTN: International Standard Randomised Controlled Trial Number; MMHSCT: Manchester Mental Health and Social Care Trust; MRC: Medical Research Council; NHS: National Health Service; NIHR: National Institute for Health Research; NPT: Normalisation Process Theory; NRES: National Research Ethics Service; PI: principal investigator; PMG: Programme Management Group; PPI: Patient and Public Involvement; PROM: Patient Reported Outcome Measure; PSG: Programme Steering Group; QALYs: Quality-Adjusted Life Years; RCT: randomised controlled trial; REC: Research Ethics Committee; TIDieR: Template for Intervention Description and Replication; VAS: visual analogue scale; UK: United Kingdom; VSSS: Verona Service Satisfaction Scale; WEMWBS: Warwick-Edinburgh Mental Well-being Scale; WHOQOL: World Health Organisation Quality of Life.

Competing interests

The authors declare that they have no competing interests.

Authors' contributions

PB is co-applicant and co-lead of Work stream 2, designed the study and co-wrote the first draft of the protocol, and revised and edited subsequent versions for publication. CR is trial statistician and co-applicant, contributed to the study design, performed the sample size calculation and will oversee analyses. NOL contributed to the study design and statistical analysis plan for the study. PCal is PI (University of Nottingham) and co-applicant and contributed to the study design. PBe is Work stream 1 lead and co-applicant and contributed to the study design. CF is programme manager and revised and edited the manuscript. CG is research fellow, assisted with trial design, selection of outcome measures and co-wrote the first draft of the protocol. NO is research associate and assisted with the first draft of the protocol. AR is Work stream 3 lead and co-applicant and contributed to study design. LD is co-applicant and designed the health economic evaluation. RD is co-applicant, psychiatrist and Trust champion. CS is co-applicant and contributed to the study design. OM is research fellow and Nottingham recruitment lead. AG is co-applicant and service user researcher. LW is co-applicant and service user researcher. LC is co-applicant and carer researcher. KB is programme manager and governance lead and contributed to study planning. HB is study research associate. SB is study research associate. PC is co-applicant, co-lead for patient and public involvement and Trust champion. AR is Executive Nurse at MMHSCT and Trust champion for the study. KL is CI of the Programme Grant and co-lead of Work stream 2, co-designed the study and designed the training programme. All authors read and approved the final manuscript.

Acknowledgments

The EQUIP project is funded by the National Institute for Health Research's Programme Grants for Applied Research (RP-PG-1210-12007). The views expressed are those of the authors and not necessarily those of the NHS, the NIHR or the Department of Health.

The trial team wishes to acknowledge the support of the NIHR Clinical Research Networks.

Roles and responsibilities

Trial sponsor

Manchester Mental Health and Social Care Trust (contact: Dr Andy Mee)

Programme Management Group (PMG)

Karina Lovell, CI
Patrick Callaghan, PI
Kathryn Berzins, Programme Manager
Claire Fraser, Programme Manager
Work stream Leads: Penny Bee, Peter Bower, Anne Rogers
Study co-applicants: Patrick Cahoon, Lindsey Cree, Linda Davies, Richard Drake, Andrew Grundy, Chris Roberts, Caroline Sanders, Lauren Walker
Researchers: Susan Beatty, Helen Brooks, Chris Gibbons, Matthew Hamilton, Oonagh Meade, Nicola Olleveant, Neil O'Leary

The PMG will meet every 3 months to review the progress of a study and recommend any changes necessary to the protocol to facilitate smooth running of the study.

Programme Steering Group (PSG)

Independent chair:
Alan Simpson, City University London
Independent members:
Michael Coffey, University of Swansea
Dean McMillan, University of York
Bethan Edwards, Service user representative
Trial team:
Karina Lovell, Chief Investigator
Kathryn Berzins, Programme Manager
Claire Fraser, Programme Manager

The PSG will meet every 6 months and undertake other business via Email. The PSG will have responsibility for monitoring progress of the trial, adherence to the protocol, patient safety and adverse events, and consideration of new information. Membership of the PSG will include the CI along with an independent chair, and at least two other independent members. The trial manager and the trial statistician will attend when appropriate. The nature of the trial is such that a data monitoring committee is not required. The PSG will oversee the quality of the data being collected from the study, assisted by the trial manager and statistician.

Author details

[1]NIHR School for Primary Care Research, Manchester Academic Health Science Centre, University of Manchester, Williamson Building, Oxford Road, Manchester M13 9PL, England. [2]School of Nursing, Midwifery and Social Work, University of Manchester, Jean McFarlane Building, Oxford Road, Manchester M13 9PL, England. [3]Centre for Biostatistics, Institute of Population Health, University of Manchester, Jean McFarlane Building, Oxford Road, Manchester M13 9PL, England. [4]School of Health Sciences, University of Nottingham, Queen's Medical Centre, Nottingham NG7 2HA, England. [5]Faculty of Health Sciences, University of Southampton, Highfield, Southampton SO17 1BJ, England. [6]Centre for Health Economics, Institute of Population Health, University of Manchester, Jean McFarlane Building, Oxford Road, Manchester M13 9PL, England. [7]Manchester Mental Health and Social Care Trust, Chorlton House, 70 Manchester Road, Chorlton-cum-Hardy, Manchester M21 9UN, England.

Received: 1 May 2015 Accepted: 3 August 2015
Published online: 13 August 2015

References

1. Department of Health. The NHS Plan: a plan for investment, a plan for reform. London: 2000.
2. Royal College of Physicians. Advance care planning (National Guidelines, Number 12): Concise guidance to good practice. London: 2009.
3. Care Services Improvement Partnership and National Institute for Mental Health in England. Our choices in mental health: a framework for improving choice for people who use mental health services and their carers. London: 2005.
4. Healthcare Commission. The pathway to recovery: a review of NHS acute inpatient mental health services. London: 2008
5. Department of Health. Effective care co-ordination in mental health services: modernising the care programme approach - A policy booklet. London: 1999.
6. Department of Health. Refocusing the care programme approach. Policy and Positive Practice Guidance. London: 2008.
7. Care Quality Commission. Survey of mental health inpatient services. London: 2009.
8. Healthcare Commission. Community mental health service users' survey. London: 2008
9. Crawford MJ, Rutter D, Manley C, Weaver T, Bhui K, Fulop N. Systematic review of involving patients in the planning and development of health care. Br Med J. 2002;325:1263–7.
10. Wilkinson C, McAndrew S. 'I'm not an outsider, I'm his mother!' A phenomenological enquiry into carer experiences of exclusion from acute psychiatric settings. Int J Ment Health Nurs. 2008;17(6):392–401.
11. Rethink. Involving carers in out of area treatments: a good practice guide. London: 2007.
12. Rethink. You care, you count: a carer's guide to getting support. London: 2009.
13. Rethink. Caring and coping: the guide for people caring for someone with mental illness. London: 2009.
14. Bee PE, Playle JF, Lovell K, Barnes P, Gray R, Keeley P. Service user views and expectations of UK-registered mental health nurses: a systematic review of empirical research. Int J Nurs Stud. 2008;45:442–57.
15. Murray DM. Design and analysis of group-randomized trials. Oxford: Oxford University Press; 1998.
16. Torgerson DJ. Contamination in trials: is cluster randomisation the answer? Br Med J. 2001;322(7282):355.
17. Greenhalgh T, Robert G, Bate P, MacFarlane F, Kyriakidou O. Diffusion of innovations in health service organisations. Oxford: Blackwell; 2005.
18. Hoffmann TC, Glasziou PP, Boutron I, Milne R, Perera R, Moher D, et al. Better reporting of interventions: template for intervention description and replication (TIDieR) checklist and guide. Br Med J. 2014;348:g1687.
19. Ludman EJ, Simon GE, Rutter CM, Bauer MS, Unutzer J. A measure for assessing patient perception of provider support for self-management of bipolar disorder. Bipolar Disord. 2002;4:249–53.
20. Rasch G. Probabilistic models for some intelligence and attainment tests. Chicago: University of Chicago Press; 1980.
21. Ruggeri M, Lasalvia A, Dall'agnola R, van Wijngaarden B, Knudsen HC, Leese M, et al. Development, internal consistency and reliability of the Verona Service Satisfaction Scale – European Version – EPSILON Study 7. Br J Psychiatry Supp. 2000;177:S41–8.
22. Ruggeri M, Dall'agnola R. The development and use of the Verona expectations for care scale (VECS) and the Verona service satisfaction scale (VSSS) for measuring expectations and satisfaction with community-based psychiatric-services in service users, relatives and professionals. Psychol Med. 1993;23(2):511–23.
23. Lelliott P, Beevor A, Hogman G, Hyslop J, Lathlean J, Ward M. Carers' and users' expectations of services – carer version (CUES-C): a new instrument to support the assessment of carers of people with a severe mental illness. J Ment Health. 2003;12(2):143–52.
24. Waddell L, Taylor M. A new self-rating scale for detecting atypical or second-generation antipsychotic side effects. J Psychopharmacol. 2008;22(3):238–43.
25. Tennant R, Hiller L, Fishwick R, Platt S, Joseph S, Weich S, et al. The Warwick-Edinburgh Mental Well-being Scale (WEMWBS): development and UK validation. Health Qual Life Outcomes. 2007;5:63.
26. Ridgway PA, Press A. Assessing the recovery commitment of your mental health services: a user's guide for the Developing Recovery Enhancing Environments Measure (DREEM). Recovery Devon; 2004
27. Zigmond AS, Snaith RP. The Hospital Anxiety and Depression Scale. Acta Psychiatrica Scandinavica. 1983;67(6):361–70.
28. Gaston L. Reliability and criterion-related validity of the California Psychotherapy Alliance Scales – patient version. Psychological Assess. 1991;3(1):68.
29. Skevington SM, Lotfy M, O'Connell KA. The World Health Organization's WHOQOL-BREF quality of life assessment: psychometric properties and results of the international field trial - A report from the WHOQOL group. Qual Life Res. 2004;13(2):299–310.
30. Janssen MF, Pickard AS, Golicki D, Gudex C, Niewada M, Scalone L, et al. Measurement properties of the EQ-5D-5L compared to the EQ-5D-3L across eight patient groups: a multi-country study. Qual Life Res. 2013;22(7):1717–27.
31. Sterne JAC, White IR, Carlin JB, Spratt M, Royston P, Kenward MG, et al. Multiple imputation for missing data in epidemiological and clinical research: potential and pitfalls. Br Med J. 2009;338:b2393.
32. Campbell M, Fitzpatrick R, Haines A, Kinmonth AL, Sandercock P, Spiegelhalter D, et al. Framework for design and evaluation of complex interventions to improve health. Br Med J. 2000;321(7262):694–6.
33. May C, Finch T, Mair FS, Ballini L, Dowrick C, Eccles E, et al. Understanding the implementation of complex interventions in health care: the Normalization Process Model. BMC Health Serv Res. 2007;7:148.
34. Hutton JL. Are distinctive ethical principles required for cluster randomized controlled trials? Stat Med. 2001;20(3):473–88.
35. Edwards SJL, Braunholtz DA, Lilford RJ, Stevens AJ. Ethical issues in the design and conduct of cluster randomised controlled trials. Br Med J. 1999;318(7195):1407–9.
36. Richards DA, Hill JJ, Gask L, Lovell K, Chew-Graham C, Bower P, et al. Clinical effectiveness of collaborative care for depression in UK primary care (CADET): cluster randomised controlled trial. Br Med J. 2013;347:4913.
37. Kennedy A, Bower P, Reeves D, Blakeman T, Bowen R, Chew-Graham C, et al. Implementation of self-management support for long term conditions in routine primary care settings: cluster randomised controlled trial. Br Med J. 2013;346:f2882.
38. Rick J, Graffy J, Knapp P, Small N, Collier DJ, Eldridge S, et al. Systematic techniques for assisting recruitment to trials (START): study protocol for embedded, randomized controlled trials. Trials. 2014;15:407.

Submit your next manuscript to BioMed Central and take full advantage of:

- **Convenient online submission**
- **Thorough peer review**
- **No space constraints or color figure charges**
- **Immediate publication on acceptance**
- **Inclusion in PubMed, CAS, Scopus and Google Scholar**
- **Research which is freely available for redistribution**

Submit your manuscript at www.biomedcentral.com/submit

BioMed Central
