# Supplementary material for: Embedding shared decision-making in the care of patients with severe and enduring mental health problems: The EQUIP pragmatic cluster randomised trial
Source: PLoS One. 2018 Aug 22;13(8):e0201533. doi: 10.1371/journal.pone.0201533 (PMC6104914; doi:10.1371/journal.pone.0201533)
Supplement: S2 Protocol — (PDF) [file pone.0201533.s004.pdf]

TITLE OF TRIAL:

ENHANCING THE QUALITY OF USER INVOLVED CARE PLANNING IN MENTAL HEALTH SERVICES (EQUIP): CLINICAL CLUSTER RANDOMISED CONTROLLED TRIAL AND PROCESS EVALUATION

Trial Identifier:

ISRCTN16488358

Date of ISRCTN assignation:

14/05/2014

Link to record in ISRCTN Register:

http://www.controlled-trials.com/ISRCTN16488358/

PROTOCOL: ENHANCING THE QUALITY OF USER INVOLVED CARE PLANNING IN MENTAL HEALTH SERVICES (EQUIP)

Research programme aims and objectives

Aim

The overall aim of the programme grant is to improve user and carer involvement in care planning in mental health services.

Major objectives

- To develop, evaluate, implement and disseminate a user/carer-led training package for mental health professionals to improve the extent users and carers are involved in care planning.
- To develop and validate a patient reported outcome measure (PROM) to assess improvements in user/carer involvement
- To assess the clinical and cost effectiveness, feasibility and acceptability of this training package using a cluster randomised clinical control trial design.
- To identify the individual and organisational barriers and facilitators to implementing effective user and carer involvement in care planning.
- To use a multifaceted approach to comprehensively disseminate our findings.

These objectives are covered by four work streams incorporating seven studies.

This protocol is for work stream 2, study 5 which is the clinical randomised control trial (CRCT) and work stream 3, study 7 which is the process evaluation of the trial.

Work stream 1 (Studies 1-4)

Develop a user/carer led training programme for mental health professionals and a measure of user/carer involvement in care planning.

Work stream 2 (Study 5)

Evaluate the efficacy and cost effectiveness of user/carer involved care planning

Work stream 3 (Studies 6 -7)

Implement user/carer involved care planning by understanding the individual and organisational barriers and facilitators and examine the processes involved in the development and use of user/carer involved care planning.

Work stream 4

Disseminate user / carer involved training materials and resources for health professionals and users and carers of mental health services.

Background

Involving service users in their care and providing choice is at the centre of policy initiatives aimed at improving quality of care. This principle is enshrined and prioritised in healthcare particularly mental health care policy and guidelines. There is a wealth of evidence that users and carers want significantly more involvement in the care planning process. However there is substantial empirical evidence that the majority of users and carers are marginalised in the care planning process.

Importance of proposed research

This research is important because it provides an opportunity to make a quality improvement in mental health services which involves users and carers as co-producers of health care. Of importance is that such a quality improvement has the potential to be translated over both mental health and physical care settings and hence benefit many thousands of service users

Research team

Our team is multidisciplinary with significant content, methodological, clinical, academic, lived experience and educational expertise.

Research environment

The research will be undertaken in NHS settings (Manchester Mental Health and Social Care Trust and Nottinghamshire Healthcare NHS Trust, South West Yorkshire Partnership NHS Foundation Trust , Leicestershire Partnership NHT Trust) in collaboration with the Universities of Manchester and Nottingham.

Outputs and impact

The outputs and impact of this programme grant have the potential to be substantial. We will have developed and delivered in partnership with users and carers a training programme to improve user and carer involvement in care planning. A user/carer patient reported outcome measure (PROM) will be developed and validated. We will know the effectiveness and cost effectiveness of user and carer involved care planning. We will have developed implementation guidelines based on our findings of the organisational and individual facilitators and barriers to user and carer involved care planning. We will develop tools to disseminate findings from the projects, including a care planning audit tool, user and carer materials for users and carers to empower them to facilitate change, and training materials which can be used across mental health services. We will offer these materials to up to ten NHS mental health trusts and advise on improving user and carer involved care planning.

Research governance

The programme management group will meet every three months to discuss the progression and day to day management issues of the programme grant and will include the chief investigator (CI), all other investigators, and programme managers (PM). The CI will be responsible for the overall leadership, management and outputs of the programme. Each work stream will be led by a named work stream lead. The CI will maintain a log of the key milestones to be achieved against the timetable. Progress of these milestones and corresponding timetable will be reported at management meetings to ensure progression of the programme and to agree corrective action if necessary. The PMs will be responsible for the day to day running

and coordination of the programme and will be accountable to the CI. All research associates will be supervised by stream leads and overseen by the CI and site PIs.

Programme Steering Committee

A programme steering committee (PSC) has been established and comprises an independent chair who has expertise in programme grants and care planning and three other independent members including a user representative who has had lived experience, a carer and a clinician who has expertise and experience of working in community teams. The composition of the PSC was agreed by the National Institute for Health Research (NIHR).

A full risk assessment of the programme has been conducted by the CI and PM. A risk register has been developed and potential risks of the study identified to enable any necessary mitigating actions to take place. A RAG rated system (Red, Amber, Green) is in place (in addition to a matrix measuring each risk on likelihood of risk occurring (low/medium/high) and impact if risk did occur. The risk register is managed and monitored by the PM and CI and is a standing agenda item at each programme management meeting.

Sponsorship

Manchester Mental Health and Social Care Trust is the sponsor.

Service user and carer involvement

We have extensive user and carer involvement. Within our NIHR funded programme development grant we argued that it was difficult for both users and carers and academics to work meaningfully together as users and carers were unfamiliar with research methods and concepts. To overcome this we ran a successful six day interactive research methods course which resulted in positive feedback from users and carers. Many of the applicants were responsible for teaching their areas of expertise (e.g. Professor Anne Rogers – qualitative research; Professor Linda Davies – health economics; Professor Peter Bower - literature searching and trial design). We taught the course at a similar level to our MRes but worked in small groups with up to three facilitators. The course was devised and led by Dr Baker (Co-Investigator)

and Professor Lovell (CI). The course has been cited by the Mental Health Research Network (MHRN) as an exemplar of good practice. In addition we ran a half day workshop explaining the nature of programme grants and shared our thoughts on the proposed grant and obtained helpful feedback. A full day meeting of users and carers and the trial team was conducted to determine the measures for the evaluation phase.

We have identified a range of roles for the service users and carers who have participated in the programme development grant. We have a large advisory group (n=16) of users and carers, and two service users and one carer are formal co-applicants and have worked with the research team to co-facilitate focus groups and interviews during EQUIP studies 1 and 2. One further service user is also a member of the programme steering committee. Users and carers will be participating in the overall management of the research, in developing participant information resources, undertaking and analysing the research, contributing to the reporting of the study report and in the dissemination of research findings.

Personnel

The team is multidisciplinary with appropriate clinical, educational, methodological and service delivery expertise, supported by those with lived experience of mental illness and its management.

Chief Investigator:	Professor Karina Lovell
Work stream 2 Lead:	Professor Pete Bower
Work stream 3 Lead:	Professor Anne Rogers
Site leads:	Professor Karina Lovell (University of Manchester)<br>Professor Patrick Callaghan (University of Nottingham)
Co-applicants:	John Baker, Penny Bee, Patrick Cahoon, Lindsey Cree, Linda Davies, Richard Drake, Andrew Grundy, Chris Roberts, Anne Rogers, Anita Rolfe, Caroline Sanders, Lauren Walker
Programme managers:	Kathryn Berzins and Claire Fraser
Research team:	Susan Beatty, Helen Brooks, Chris Gibbons, Matthew Hamilton, Oonagh Meade, Neil O'Leary, Nicola Olleveant, Rebecca Pedley
Trainers:	Deborah Bhatti, Debbie Butler, Donna More

This protocol is a combined document for work stream 2, study 5 which is the clinical cluster randomised controlled trial and work stream 3, study 7 which is the process evaluation of the trial. The remaining protocol will discuss each study separately under each heading for clarity.

Aims

Work stream 2 (Months 18-48)

The aim of this work package is to evaluate the efficacy and cost effectiveness of a user/carer involved training package developed earlier in the programme grant.

This aim will be achieved through the undertaking of study 5.

Study 5

- To determine if a user/carer led training package is effective in increasing user/carer involvement in care planning and improving health outcomes for service users with severe mental illness under the care of community teams.
- To determine if a user/carer led training package is cost-effective in improving short term health outcomes for service users with severe mental illness under the care of community teams.

Work stream 3 (Months 0-48)

The aim of this work package is to understand professionals' and users/carer perspectives about the factors that inhibit or promote user involvement and the integration of care planning into clinical settings. Furthermore, the package will investigate the impact of the training package to enhance user involvement in care planning.

The aim will be achieved through the undertaking of two studies (study 6 and 7):

Study 6

Conduct a mapping exercise in organisational structures and policies related to care planning which is reviewed and updated over the course of the project (approved by Manchester University Research Ethics Committee ref: 13304).

Study 7:

The aims of this study are to examine: (i) how user/carer involved care planning training and its principles impacts on and is incorporated into existing routine clinical practices; (ii) how care planning affects the way in which professionals relate to, communicate with and negotiate therapeutic options with users; (iii) how the new care planning training and arrangements impact on existing methods of coping, self-care and the development of service user expertise and how it shapes and transforms relationships between service users and professionals; (iv) the impact on networks, a sense of control, security and identity compared to previous care planning practices; (v) service users' perceptions of their preparation and support in relation to engaging with the form and content of the new system of care planning and its benefits and use .

The objectives are to examine:

- How training for user involved care planning and its principles impacts on and is incorporated into existing routine clinical practices;
- How care planning affects the way in which professionals relate to, communicate with and negotiate therapeutic options with users;
- How the new care planning training and arrangements impact on existing methods of coping, self-care and the development of service user expertise and how it shapes and transforms relationships between service users and professionals;
- The impact of training on service users' perceptions of networks, a sense of control, security and identity compared to previous care planning practices;
- Service users' perceptions of their preparation and support in relation to engaging with the form and content of the new system of care planning.

Background

Study 5

The full programme grant application describes the background to the need for the programme as emanating from the observation of the increased importance being attributed to involving service users in their care, whilst at the same time the majority of service users and carers still feeling marginalised in the care planning process. There is evidence that service users and carers want significantly more involvement in the care planning process, but this is not always achieved (DoH 2000, Royal College of Physicians 2009). There are inconsistencies in practice, and embedded within this

there is poor communication (Healthcare commission 2008). Compounding these problems, there are also problems with the quality of the relationships with and between professionals at all levels (Blenkiron et al 2003, Bramesfeld et al 2007, Mind 2010).

This research is important because it seeks to develop a standardised training package to achieve better care planning, and to test the efficacy and cost effectiveness of this package. It will provide an opportunity to improve the quality of mental health care across community and rehabilitation inpatient mental health services.

Study 7

A process evaluation of the training programme delivered as part of the trial is deemed appropriate because successful implementation of the user/carer led care planning implicates a range of factors including the integrity of the intervention and the acceptability of the intervention to both clinicians and service users (Greenhalgh et al, 2005). This current study is designed to explore how far the user/carer led care planning has been taken up by and implemented in the daily work of the health professionals who attended the training and what the consequences of this uptake has been. It will complement and supplement the evidence provided by the main randomised trial (as recommended by the MRC framework for evaluation of complex intervention, Campbell et al, 2000).

Where results from the trial are positive, the process evaluation will consider the conditions, mechanisms and processes that gave rise to this success to help translation into other areas. Conversely, if findings are negative or inconclusive, the process evaluation can examine the sources or barriers to implementation and consider why these negative results are observed.

Method

Study 5

Study Design

The study will adopt a cluster randomised trial design. The training package will be delivered to clinical staff working in community teams. A cluster design is required to avoid contamination.

We will adopt a mixed design, including both a 'cluster cohort' design, and a 'cluster cross sectional' design (see Figure 1 for an outline of the design, and Figure 2 for the CONSORT flow diagram).

In the 'cluster cohort' design, we will recruit service users cared for by each community team and conduct a detailed face-to-face assessment at baseline. Each community team will then be randomised to either intervention (training in care planning) or control (usual care planning). We will then train the intervention community teams in care planning, and conduct another detailed face to face assessment with the same service users 6 months after the baseline assessment.

The design will also include a 'cluster cross sectional' element. Six months after randomisation, we will distribute a questionnaire to all service users who are not part of the 'cluster cohort' but who are under the care of all community teams using a simple postal questionnaire.

The advantages of the design are outlined in Box 1.

Box 1 Advantages of the mixed design

The 'cluster cohort' design allows more accurate adjustment for baseline characteristics at an individual level, giving increased statistical power.

However, for the cluster cohort, service users need to participate in two relatively long face to face assessments, which may be burdensome to service users or difficult to organise if people move often. This means the cluster cohort may be vulnerable to recruitment issues (where only a small number of eligible service users take part) and attrition (i.e. where service users do not attend for follow-up), both reducing external validity.

The 'cluster cross-sectional' design can help ameliorate these problems. Service users only have to agree to assessment once, and the assessment is designed to be less burdensome as it includes fewer measures. This means that a higher proportion of patients may agree to take part and be retained in the study, potentially increasing the sample size and the external validity of the results. However, it will tend to have less power due to the reduced ability for baseline adjustment. We will sample a proportion of eligible service users for the 'cluster cross-sectional' design to reduce cost and administrative burden.

Adoption of the combined design provides protection against problems in either of the individual approaches.

A potential threat to the validity of a cluster randomised trial is recruitment bias, where professionals allocated to different trial arms recruit differently depending on their allocation, leading to selection bias and baseline incomparability (Torgerson 2001). Whilst it is preferable to recruit patients prior to allocation, the logistics of the trial means that clusters will need advance notice of their training date, which will require us to inform them of their allocation. However, initial patient selection in the EQUIP trial is not by professional referral, but will use existing registers of patients. This will be undertaken by the Mental Health Research Network (MHRN), Clinical Studies Officer (CSO). This will limit the ability of professionals to influence recruitment, as their only impact will be to exclude patients. We will stress the importance of including all eligible patients, provide guidance on exclusion criteria and report details of all exclusions by trial arm in the study report.

Once service users have been recruited, the clusters will be allocated randomly to either intervention or control. To reduce selection bias, allocation will be determined through an external telephone randomisation service at the Clinical Trials Unit of the Manchester Academic Health Science Centre. Clusters will be submitted to the randomisation service in pairs. Each pair will be from the same site (Manchester/Nottingham/SW Yorkshire/Leicestershire) and similarly matched in other characteristics where possible. One member of the pair will be allocated to intervention by random selection, the other allocated to control. To reduce detection bias, we will seek to blind researchers undertaking assessments of the quality of care planning to the group to which clusters have been allocated. We will report the success or otherwise of our attempts at blinding.

Service user/carer consent

Participants in randomised trials usually provide written informed consent for a range of research procedures, including participation in the trial, randomisation and data collection.

However, conventional informed consent procedures are not always appropriate in the context of a cluster, randomised trial (Hutton, 2001). In the EQUIP trial, community mental health services and their constituent community teams are making the decision to take part in the EQUIP trial and agree to randomised allocation. This is described as a 'cluster cluster' design, and is distinguished from an 'individual cluster' design (Edwards, 1999). In the latter, randomisation is at the level of the cluster, but specific services are delivered to individuals, and service users can

consent to receive or not receive that intervention. Our recent CADET trial was an example of an individual cluster design (Richards et al 2013) In 'cluster cluster' designs such as EQUIP, service users cannot opt out of a cluster in the same way, as the community teams will have been trained in the new methods. Our recent WISE study was an example of this design (Kennedy et al 2013).

Not all cluster randomised trials seek individual patient consent (Eldridge, 2005). In the EQUIP trial, seeking formal consent for participation and randomisation may be inappropriate, as these processes are not under control of the service users. Therefore, we seek to adopt the following consent procedures.

Service managers and staff will act as 'ethical guardians' for their service users. If the service, and the community teams consent to take part in the trial, then individual service users will not be asked for specific consent to be randomised as part of the EQUIP trial. Service users cannot therefore 'opt out' of their cluster allocation. The Mental Health Research Network (MHRN), Clinical Studies Officers (CSOs) will be responsible for accessing patient details and determining who is eligible to take part in the study and be contacted. They will be responsible for sending out information about the study to the identified service users, along with an invitation to participate. The research team will not have access to service user details until they have returned the consent to contact form.

Service users in the 'cluster cohort' will undertake a detailed face to face assessment at two points in time (baseline and 6 months). For these, we will adopt a formal written consent procedure. We will explain to service users that their community teams are involved in a study to test the effectiveness of a new training package on service user/carer involvement in care planning compared to the usual care planning experience. Participants will be told that we will do this by delivering the training to some mental health teams and not to other teams to see if receiving the training has an impact on the extent of service user/carer involvement in care planning.

Carers in the 'cluster cohort' will undertake a postal survey at baseline and at six months. Carers will be asked to participate via nomination by a service user. Response to the questionnaires will be treated as consent. Carers will also be asked to complete a baseline questionnaire and a consent to contact form in six months to allow the follow up questionnaire to be completed which is returnable to the research team in the prepaid envelope provided.

Service users in the ‘cross sectional sample’ will undertake a short postal survey at six months only. For these, we will treat this part of the study as a survey. Service users will receive a postal invitation to the survey, seeking their views on the quality of the care planning process that they have received. Response to the survey will be treated as consent, as is usual in survey work. Respondents will receive a £5 voucher for completing the survey. A follow up letter may be sent to encourage responses.

We have utilised these procedures in a previous, similar study conducted as part of another NIHR programme grant (RP-PG-0407-10136, ethical approval Salford and Trafford, 09/H1004/6 Amendment 3).

The study will be registered on a public database prior to recruitment of patients and will receive an appropriate ISRCTN.

Figure 1 EQUIP cluster randomised trial design

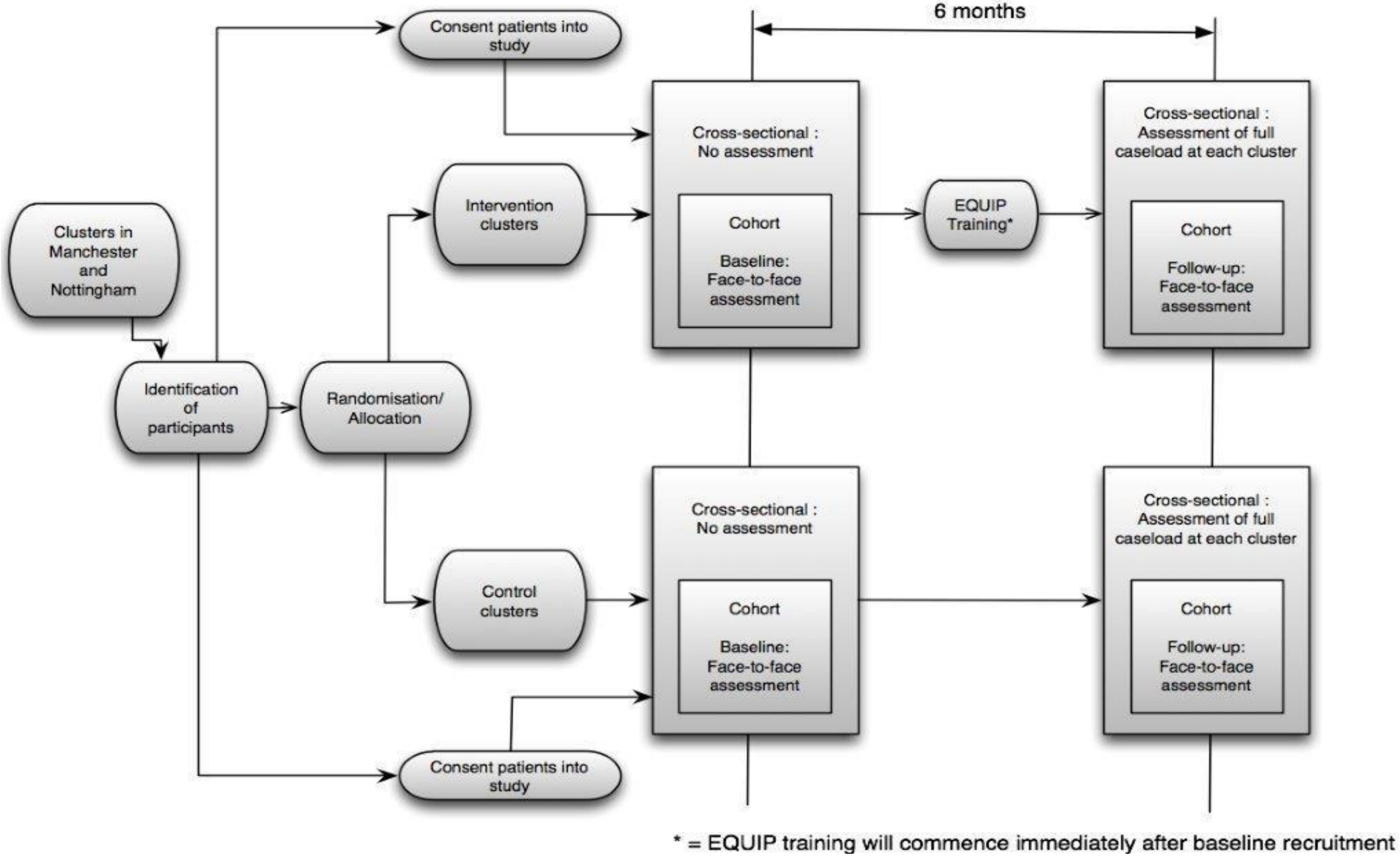

Figure 2 EQUIP CONSORT Flow Diagram

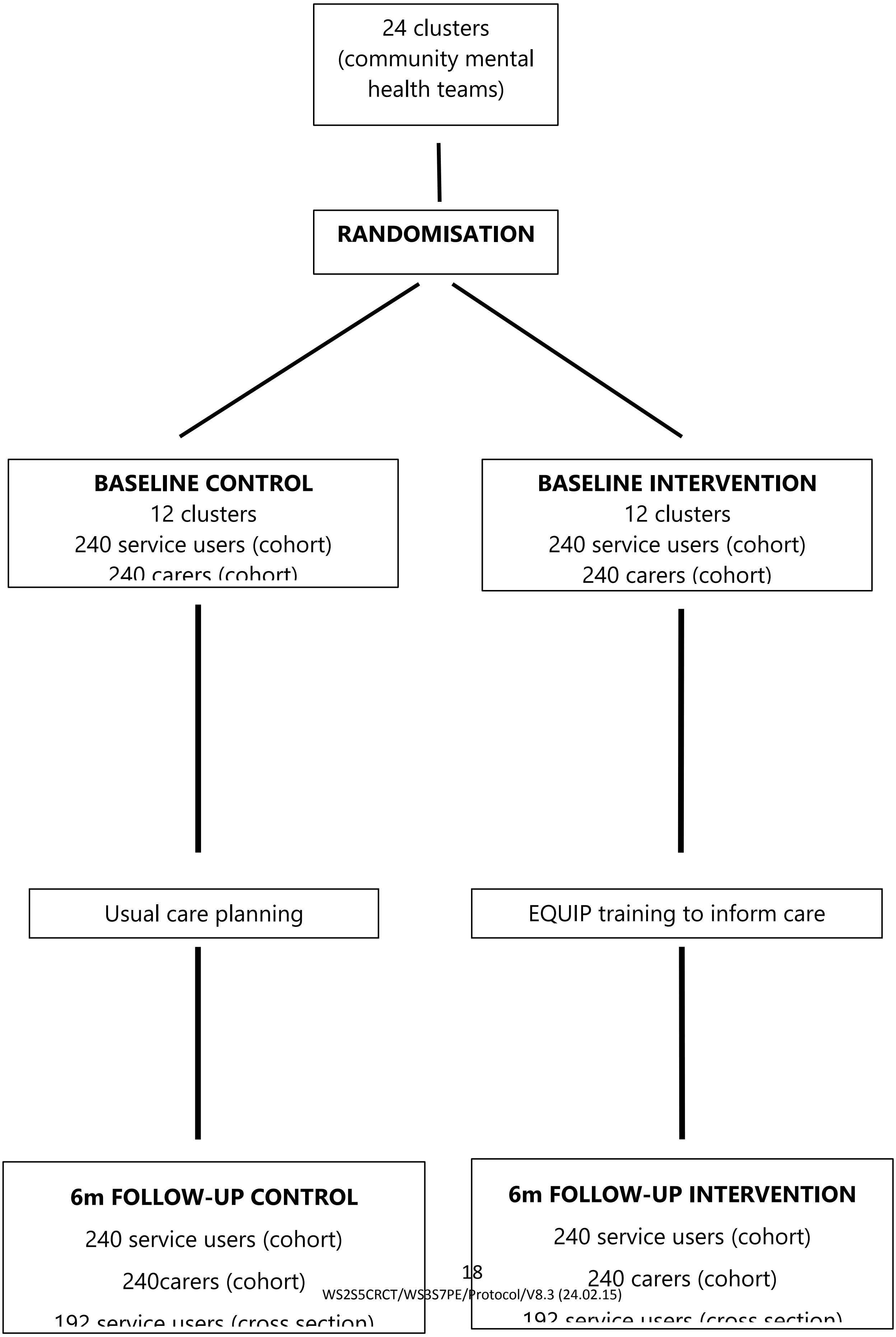

Study 7:

Study Design

The study will involve a qualitative process evaluation exploring embedding and implementation nested within the trial (work stream 2, study 5) using multiple qualitative methods comprising:

- Semi-structured in-depth interviews with professionals, service users, carers at multiple time-points;
- Observation of how service users and staff approach, adopt and use the new user/carer involved care planning;
- Diary records of user and carer experiences, practices and uses of care planning;
- Social network approach to explore the role of social networks in care planning and the impact of user/carer involved care planning on these networks and network dynamics and access to new resources.

In-depth and network interviews

Service users: Service users (15-20) (and where possible carers 5-10) in the intervention cluster will be interviewed prior to the introduction of the care planning training and at two subsequent time-points in order to capture processes and change (6 and 12 months) (Conrad, 1990). This is important because our previous work indicates that the introduction and incorporation of new practices can be initially disruptive followed by a period of re-adjustment (Gately et al. 2008; Rogers et al. 2011). The interviews will explore the experience of care planning and everyday management of mental health problems for service users, the nature of interaction with staff and the degree to which care planning is viewed/ experienced as empowering or constraining in relation to previous systems of care planning. We will also interview up to 10 service users in the control arm of the study at the time points detailed above to compare the findings with the intervention arm.

Service users will also be asked to complete a social network diagram relating to the members who are perceived to input into care planning and this will be redone at subsequent follow up interviews to investigate changes in networks. The network

approach to be undertaken will follow personal network 'concentric circles' method (Pahl and Spencer 2004). Participants will be presented with a diagram containing three concentric circles. They will be asked to place those people or places they consider most important in relation to care planning in the central circle, those considered important but not as important as those in the central circle in the middle circle and those who they consider important but not as important as those in the central two circles in the outer circle. Network members can include any sources of support including family, friends, health professionals, pets, community groups, internet support groups etc. We will draw on a method we have used previously whereby after individuals plot their networks, questions will be asked about the role of these individual network members in relation to care planning. The broad focus will explore which relationships are most important to care planning, the relationships that develop as part of care planning involvement over time, resource access and the wider role of social networks and relationships in relation to care planning.

Service users will be told that the interviewer does not know which arms of the study their care team was in and not to mention individual practitioner or team names when completing the network diagram to reduce the likelihood of unblinding the researcher as to group allocation.

Professionals: Staff in the intervention cluster (15-20) will be interviewed prior to the introduction of the care planning training and at two subsequent time-points in order to capture processes and change. Interviews with professionals will focus on their views, expectations and experiences of care planning training within the broader contextual issues regarding the organisation and delivery of care for people with severe mental illness. They will be asked to reflect on professional experience of barriers and enablers for high quality care. They will be prompted to draw on examples from clinical practice in order to illustrate views and experiences. Similar work has been undertaken in other illness areas (Mair et al. 2008). We will also interview up to 10 staff members in the control arm of the study at the time points detailed above.

Observation

The inclusion of an ethnographic approach is relevant for studying complex care trajectories and for key consultations (Allen et al. 2004). Structured observational methods will be used to focus on professional and service user/carer interactions

with professionals in care planning meetings. Observation will include attention to how the system fits into the everyday routines of management and care practices for service users and professionals. We will also spend at least one day shadowing each of the community teams or inpatient facilities in the study in order to observe how information is introduced interpreted and responded to, and to observe the impact of the training on clinical encounters.

Diaries

This method is designed to capture salient moments in the use of new practices (e.g. difficulties or when aspects of the care plan were particularly useful). A number of studies have highlighted the utility of diaries for recording change in experience and management of long term conditions including mental health and voice diaries have been successfully used. Service user participants will be offered a choice of format (written or audio), and the frequency of diary entries will be flexible to ensure people are not over-burdened.

Study setting and sample size

Study 5

Recruitment and randomisation of community teams and rehabilitation inpatient facilities

Twenty four community teams will be recruited, from several different geographical areas (Manchester, Nottingham, South West Yorkshire and Leicestershire). All community teams in each geographical area will be eligible for inclusion. Community teams will be randomly allocated to receive the user/carer-led training package in care planning or to continue with usual practice.

Recruitment and training will be undertaken in sequence, to maximise efficiency in delivery of training to community teams, but also to ensure there is sufficient time to permit the relevant baseline assessments to be undertaken with service users, cared for by each community team.

We will obtain lists of service users from community teams and then the teams (clusters) will be randomised. Around the same time as randomisation (but before

the training intervention has occurred), the service users will be contacted by postal invite with one follow up phone call (using the lists provided), consent obtained and baseline assessments undertaken. This is to ensure that there is a maximum period of time between the baseline assessment and the six month follow up assessment. The baseline assessment will therefore be scheduled to be undertaken as close to the training (intervention) as possible and will occur in a maximum six week period prior to the community teams being trained. Service users will be followed up six months from the baseline assessment point, aiming for follow-up assessments within two weeks of the six month deadline.

Recruitment of professionals in community teams

Trust managers have agreed that we can recruit all community teams. To recruit professionals we will use our applicants to champion the study (Drake, Rolfe, Lovell and Callaghan). Teams will be introduced to the trial via a letter of support from the Chief Executive, and/or via meetings with senior managers attended by the CI and PM. Meetings will also be held with area team managers (and if requested, staff) across both sites to facilitate engagement with and understanding of the trial.

Recruitment of service users/carers

To recruit service users in the 'cluster cohort', the direct care team within the community team (cluster) will produce a list of all patients who meet eligibility to participate in the trial, including any reasons for exclusions. This will be referred to as a list of their 'caseload'. These patient lists will be used by the MHRN CSO (or admin support within the Trust) to send out an introductory letter, participant information sheet and consent to contact form to each patient (appendix 6), inviting them to take part in the study. Patients will be required to 'opt in' by returning the consent to contact form in a pre-paid envelope to the research team. CSOs will contact non responders by telephone on one occasion to allow patients to opt in over the telephone. Some Trusts prefer that the initial distribution of introductory letter and participant information sheet and consent to contact form be carried out by a clinician during a routine meeting with the service user. Where this is the preferred approach, the clinician will introduce the study to the service user and give them the information pack. The clinician will discuss participation with the service user and if the service users wishes to be contacted by a university researcher to receive more information and discuss participation in the trial, the clinician will complete the

consent to contact form with the service user and pass it to the research team. This will be done by secure fax or over the telephone.

The research team will then follow up the consent to contact forms, to answer any further questions, and when a participant is recruited, the researcher will continue with a face to face informed consent process. Following signed consent, all baseline measures will be completed (table 1). In some sites CSO may be available to support with data collection. Where this is the case they will be trained by the research team with regard to collecting data and follow their own NHS Trust Lone Worker procedures.

Service users will be asked at the informed consent meeting to nominate a carer to be included in the study and if they choose to do so, will be provided with a questionnaire pack (including introductory letter, information sheet, questionnaire, pre-paid envelope and consent to contact at follow up form).

To recruit service users in the 'cluster cross sectional' study, we will conduct a postal survey of all service users under the care of each community teams six months after randomisation, excluding those already recruited to the 'cluster cohort'.

Sample size

The primary outcome is the HCCQ-10 (Health Care Climate Questionnaire) identified by our user consultation group as their preferred outcome measure. However, data on the use of this scale in service users with severe mental illness is limited, and so we have used a standardised effect to consider sample size and power. A trial with 12 clusters per arm and a mean of 20 service users per cluster is feasible within four sites (Manchester, Nottingham, South West Yorkshire and Leicestershire) combined for the cluster cohort component. This will result in a sample size of 480. A trial of this size will have power greater than 80% to detect a standardised effect size of 0.4 assuming an ICC of 0.05 and an 80% follow-up rate. Power will be increased by inclusion of baseline covariates. Additional data gathered in the cluster cross-sectional component should increase power for the corresponding analysis. We will aim to recruit the same sample size for the cross sectional survey with the same number of clusters and mean number of service users per cluster. We will assume a

loss to follow up rate of 20% for the cohort study so the sample size for the cross sectional study will be n=384 in order to be comparable to the cohort sample.

Mental Health professionals:

All mental health professionals (nurses, doctors, social care workers) and allied health professionals working in the identified community teams will be asked to participate. Maximising participation is important to ensure that all service users under the care of the community teams have the potential to benefit from the proposed training intervention. All consenting professionals allocated to the intervention group will receive user/carer-led training in care planning.

Service Users and Carers - cluster cohort study

All service users cared for by the participating community teams and meeting study inclusion criteria will be asked to participate in the 'cluster cohort' study. We aim to recruit 20 service users per cluster, with a minimum of 10 and maximum of 30.

All service users consenting to the study will be asked if there is a family member, friend or carer involved in their care. Identified carers of each service user will also be asked to participate. It is not clear how many service users in the study will have identified carers, but we will try to recruit all eligible carers (maximum of two carers per service user). The analysis of the trial is primarily focussed on the service users, and the carer data will be analysed separately.

Service Users - cluster cross-sectional study

All service users cared for by the participating community teams and meeting study inclusion criteria, who did not consent to the 'cluster cohort' study will receive a postal invitation to the survey. Three hundred and eighty four completed responses are required to give the same followed-up sample as the cluster cohort study (assuming 20% loss to follow-up rate). We will not include carers in the cluster cross sectional study.

Study 7

Semi-Structured In-Depth Interviews

Service users: A purposeful maximum variation strategy (Patton, 2001) will be deployed in order to select users from the caseload of staff teams to ensure a mix which accord to socio-demographic variables including age, socioeconomic status, diagnosis and gender. We will adopt a case study approach to follow approximately 15-20 service users and 5-10 carers over time. In addition, we will interview a number of service users (up to 10) who are not exposed to staff training in the control cluster in order to compare those in the experimental cluster.

Service users who consent to take part in the randomised control trial will be provided with an invitation letter, information sheet and consent to contact form relating to the process evaluation. If service users wish to take part they will complete the consent to contact form and return to the research team who will answer any questions and organise a time and date to take informed consent and undertake the baseline interviews.

Professionals: We will sample between 15-20 members of staff from community teams and in the intervention arm of the study involved in care planning for interview. We will distribute invitation letters, information sheets and consent to contact forms to all staff members within relevant community teams with support from the MHRN CSOs. If participants wish to take part they will complete and return the consent to contact form in the pre-paid envelope provided to the research team who will then telephone the participant to organise a time and date to take informed consent and undertake the baseline interviews. We will also interview 10 staff members from the control arm of the study.

Observation

Service users and professionals: Service users who consent to the semi-structured interviews within the process evaluation will be informed about the observation sessions and invited to participate. If they agree to participant, we will approach their care team to obtain consent from the other participants. It is envisaged that 10 observation sessions of care planning meetings will be undertaken. All relevant parties (service users and professionals) will need to consent in order for an observation session to be carried out. In addition, researchers will spend a day shadowing each of the community teams recruited into the study if they consent.

Diaries

Service users: A purposive sample of service users who consent to take part in the semi-structured interviews within the process evaluation will be invited to complete a diary. Participants will be offered both a written and audio version of the diary and the timing of diary recordings will be flexible to ensure participants do not become overburdened.

Inclusion criteria

Study 5

All community teams within Manchester and Nottingham, South West Yorkshire and Leicestershire will be eligible for inclusion. Service users aged 18 and over with a severe mental illness (e.g. psychosis, manic depressive illness) under the care of participating community teams will be eligible for inclusion. We will seek consent from service users to access health records to collect data on diagnosis, service use and treatment history.

Service users will be excluded if their participation is judged as inappropriate by the community teams, for example, if a patient is not deemed to have capacity to provide fully informed consent. We will seek to document all exclusions and report them as part of the trial CONSORT diagram.

Any carer of the service user will be eligible for inclusion in the 'cluster cohort' study if they agree to take part. Consent will be implied by response via the return of the baseline questionnaires.

Study 7

All participants consented to the cluster cohort sample of the randomised control trial will be eligible to take part in the process evaluation.

Intervention Design

Study 5

Intervention – User/carer-led Training package to inform care planning

All consenting mental health professionals (nurses, doctors and allied health professionals), will receive the training intervention developed through work stream one designed to improve user involvement in care planning. The training intervention consists of two days training, e learning package and follow-up supervision. The development and content of the training intervention is detailed in a separate protocol (Appendix 1: Training Protocol). We will document attendance at training by all professionals.

Training fidelity will be assessed by taking audio-recordings of training sessions if all participants consent. We will record as many sessions as teams consent to (from March 2015 onwards) and then, depending on the final number, sample them for analysis. We will develop a coding frame to measure adherence and competence. Adherence will be measured using our training manual and competence using other validated measures. Two independent coders to code these recordings and allow us to analyse the findings.

Comparator - Usual care

This will consist of ‘usual practice’ in care planning, without access to the specialist training described above. We will have considerable detail about what ‘usual practice’ consists of and how it varies from unit to unit from work stream three.

Study 7

N/A

Outcome Assessment

Study 5

Primary outcomes

The Health Care Climate Questionnaire (HCCQ-10, Ludman et al 2002) is the primary outcome measure for the service users in the trial.

The HCCQ-10 was developed to assess patient experience of health care and the degree to which their care offers autonomous support. The scale has 10 items, which are scored on a 7-point scale ranging from ‘strongly disagree’ to ‘strongly agree’. An overall score is calculated as the mean of the items (expressed out of 100), where a higher score indicates greater support for autonomy.

A new measure of user involvement in care planning (PROM) will be used as the primary outcome for the carers in the study (discussed in more detail in the next section).

Secondary outcomes

A new measure of user involvement in care planning (PROM) was developed in consultation with our user and carer advisory group during work stream one. The need for this measure was determined during the programme development grant as existing measures of user involvement were not deemed adequate by the advisory group. The newly developed PROM will be included as a secondary outcome to measure user and carer involvement in care planning. The new measure has excellent psychometric and scaling properties, by application to the Rasch model (Rasch 1960/1980). The scale is suitable for both service users and carers. Items are scored on a 5-point Likert scale from 'Completely disagree' to 'Completely agree'. Higher scores will reflect greater service user and carer involvement with care planning. Data from this study will provide further evidence of the acceptability, validity and sensitivity to change of this measure for this population.

Secondary outcome measures were determined using experts and a consensus discussion exercise with the user/carer advisory group. Key domains to measure were recommended by the advisory group based on proposals from the NIHR Mental Health Research Network (2010). The seven domains identified were quality of life; alliance/engagement; satisfaction; wellbeing; mental health symptoms; recovery and hope; and medication side effects. Six of these domains have one questionnaire selected for completion, whilst the domain 'satisfaction' has separate questionnaires for both service users and carers.

Satisfaction (service users)

Verona service satisfaction scale (VSSS – EU-54, Ruggeri and Dall'Agnola (1993), is a specific setting, validated, multi-dimensional, self-administered scale for measuring patients' satisfaction with mental health services. There are seven dimensions; overall satisfaction, professional skill and behaviour, access, efficacy, types of intervention and relatives involvement. Subjects are asked to express their overall feeling about their experience of the mental health service they have been attending in the last year. Satisfaction ratings are on a 5 point Likert scale, with higher scores representing greater satisfaction. Global and subscale scores can be obtained. Reliability testing has shown that the VASS-EU has good internal consistency and stability (Ruggeri et al 2000).

Satisfaction (carers)

Carers and Users' Expectations of Services – carer version (CUES-C, Lelliott et al 1999) will be used to measure carers' views of services. This is a self-rating scale consisting of 13 items each with two parts (A and B), totalling 26 questions. All questions are answered using a three point scale. There are three parts to the questionnaire; part A measures the impact of caring, part B measures the quality of support provided by carers and part C is a free text response for advice and help. Scores for each part range from 0 to 26, with higher scores representing more dissatisfaction and the need for more support. The scale has been found to be suitable to use to assess carers experiences (Lelliott et al 2003)

Medication side effects

Glasgow Antipsychotic Side-effect Scale (GASS - Waddell and Taylor 2008) is a self-rating scale to detect the side effects of antipsychotic medication. The scale consists of 22 questions and scores range from 0 to 66. Higher scores reflect more frequent experience of side effects, with total scores providing three categories of severity (absent/mild side effects, moderate side effects and severe side effects).

Well-being

Warwick Edinburgh Mental Wellbeing Scale (WEMWBS -Tennant et al 2007) is a short, psychometrically robust scale, which is easy to complete. It has 14 items scored on a 5-point Likert scale ranging from 'none of the time' to 'all of the time' based on experience over the past two weeks. Scores range from 14-70 and a higher score indicates a higher level of mental wellbeing.

Recovery and hope

Developing Recovery Enhancing Environments Measure (DREEM -Ridway and Press 2004) is a self-report measure used to assess mental health recovery of people who receive mental health services. It is a166-item questionnaire which is organised into 24 subscales (such as 'stage of recovery' and 'elements of recovery'), including a final section consisting of open ended questions. The scale is scored on a five-point Likert scale ranging from 'strongly agree' to 'strongly disagree', with low scores representing more positive experience. Dinniss et al (2007) found that DREEM was an effective and useful device for listening to the user voice.

Mental health symptoms

Hospital Anxiety and Depression Scale (HADS, Zigmond and Snaith1983) is a 14 item scale using a four-point Likert scale. Items are added to give two scores, one for anxiety and one for depression, with higher scores representing more severe symptoms. Scores range from 0 to 21 for both anxiety and depression. This is a well-used and validated measure (Barczak et al 1988, Bjelland et al 2002).

Alliance/engagement

California Psychotherapy Alliance Scale (CALPAS - Gaston and Marmar 1994) is a 12-item, self-report questionnaire which provides a total score. It has four subscales: 'the patients capacity to work purposefully in therapy', 'the affective bond with the therapist', 'therapist's empathic understanding' and 'involvement and the agreement between the patient and therapist on the goals and tasks of treatment'. Each item is rated on a six-point Likert scale, with scores ranging from 12 to 84, with higher scores representing better alliance. It has good reliability and validity (Gaston 1991).

Quality of life

World Health Organisation Quality of Life (WHOQOL-BREF - WHO 2004) is a 26-item questionnaire consisting of four domains (physical, psychological, social relationships and environment). Each question uses a five point Likert scale, ranging from a score of one to five, with higher scores representing more positive ratings. Total scores are commuted within each domain. It has been shown to demonstrate good reliability and validity (WHO 1997, The WHOQOL Group 1998)

Economic outcomes

Health Status

The EQ-5D-5L (Janssen et al 2012) measure will be used to asses health related quality of life for the economic analysis. The EQ-5D-5L, has two parts; part one, a five item questionnaire consisting of five dimensions (mobility, self-care, usual activities, pain/discomfort and anxiety/depression). Each dimension has three levels, ranging from no problems to severe problems. The five dimensions can be combined to describe the respondents' health state. Part two is a VAS which records the respondents self-rated health on a vertical VAS scale, where the end points are

labelled, ‘best imaginable health state’ and worst imaginable health state’. This information is used as a quantitative measure of health outcome as judged by the individual respondents.

Service Use Questionnaire

A measure of health service contacts (service use questionnaire) is required to allow identification of service users in the intervention arm who have had contact with a trained worker. Although this will be used to assess receipt of the intervention and the causal pathway rather than cost per se, the measure will be derived from the economic analyses.

Allocation of outcome measures

Cohort Sample

Service Users:

- Demographic data will be collected at baseline.
- Primary Outcome – The HCCQ will be administered to the cluster cohort sample at baseline and at the six month follow-up.
- Secondary Outcome – the following seven measures will be administered at baseline and six months: VSSS-54, GASS, WEMWBS, DREEM, HADS, CALPAS-12, WHOQOL-BREF, along with the PROM.
- Economic Outcome – the EQ-5D-5L and the Service Use questionnaire will be administered at baseline and at the six month follow-up.

This information will be collected via a face to face method. As the ‘cluster cohort’ assessment is more burdensome, service users will receive a £10 voucher for their time after completion of the interview at six months.

Carers:

- Demographic data will be collected at baseline.
- Secondary Outcome – The PROM will be administered to the cluster cohort sample at baseline and at the six month follow-up. The CUES-C and the WHOQOL-BREF will also be administered at baseline and at the six month follow up
- Economic Outcome – The EQ-5D-5L will be administered at baseline and at the six month follow up.

This information will be collected via a postal method. Carers will receive a £5 voucher following receipt of the questionnaires at the six month time point.

Cross-section Sample

Service Users:

- Demographic data will be collected at the six month time point.
- Primary Outcome – The HCCQ will be administered at the six month time point only.
- Secondary Outcome – PROM
- Economic Outcome - The EQ-5D-5L and the service use questionnaire will be administered at the six month time point only.

This information will be collected via a postal method. All respondents will receive a £5 high street voucher if they provide their name and postal address. Names and addresses will be requested on a separate from the questionnaire.

There will be no collection of carer information in the cross-sectional part of the study.

Table 1 shows the list of measures to be used, with which respondents, and at what time points.

Table 1 Summary of Outcome Measures

Outcome measures		BASELINE		6 MONTH FOLLOW UP		
		**Service Users (cohort)**	**Carers (cohort)**	**Service Users (cohort)**	**Carers (cohort)**	**Service Users (cross-section)**
**Primary Outcome**	Autonomy support	HCCQ-10		HCCQ-10		HCCQ-10
	User and carer involvement		EQUIP PROM		EQUIP PROM	
**Secondary Outcome**	User and carer involvement	EQUIP PROM		EQUIP PROM		
	Satisfaction	VSSS-54	CUES-C	VSSS-54	CUES-C	
	Medication side effects	GASS		GASS		
	Well-being	WEMWBS		WEMWBS		
	Recovery and hope	DREEM		DREEM		
	Mental health symptoms	HADS		HADS		
	Alliance/engagement	CALPAS-12		CALPAS-12		
	Quality of life	WHOQOL-BREF	WHOQOL-BREF	WHOQOL-BREF	WHOQOL-BREF	
**Economic Outcome**	Health status	EQ-5D-5L	EQ-5D-5L	EQ-5D-5L	EQ-5D-5L	EQ-5D-5L
	Service use	Service use		Service use		Service use

		questionnaire		questionnaire		questionnaire

Study 7

N/A

Analysis

Study 5

Statistical analysis

A draft statistical analysis plan for primary and secondary outcomes, including sub-group analyses will be presented to the Programme Steering Committee prior to the commencement of the data analysis. Analysis of outcomes will follow intention-to-treat principles: outcome data will be sought and included in the analysis for all service users irrespective of receipt of the intervention or completion of care planning during the time scale of the EQUIP trial.

Standard data checking procedures will be used as part of the data cleaning procedure prior to locking the database and linkage to group allocation. We will then model the pattern of missing data in terms of baseline characteristics of service users and treatment allocation to check for differential non-response. Depending on the patterns of missing data we may at this point choose to use multiple-imputation with deletion. This may also be used to inform possible sensitivity analyses for missing data assumptions.

For the cluster cohort study the intervention effects for the primary outcome (HCCQ) and secondary outcome measures will be estimated using a linear mixed model with a random intercept for community teams. The baseline value of the outcome will be used as a covariate together with other covariates pre-specified in the statistical analysis plan. The same statistical modelling procedure will be used for estimation of the intervention effect in the cluster cross-sectional study using a restricted set of covariates. Full detail of covariates for each model will be confirmed in the statistical analysis plan. For the primary outcome we will estimate and present the treatment effect for the cohort and cross-sectional designs separately. We will then test for heterogeneity of the treatment effect and present this pooled estimate as a secondary outcome.

Economic analysis

A cost effectiveness acceptability analysis will be conducted, from the perspectives of health and social care providers and service users, the key stakeholders in treatment decisions. The time horizon for the primary economic analysis will be at scheduled follow up (six months). Data on service use and health status (EQ-5D-5L) for the economic analysis will be collected for all participants at baseline and follow-up.

Data about the use of primary and community care based services will be collected by questionnaire completed by interview with the service users at the baseline and six month follow up assessments (cohort group). This is to ensure completeness of data collection and help to ensure that any questions or uncertainties about what should be reported can be addressed by the researcher completing the assessment. Data will also be collected from the cross-sectional group at the six month time point via the postal survey.

The service use questionnaire will ask for information about whether hospital inpatient and outpatients services have been used and if so, the name of the hospital, and this will be checked via records held by the case manager electronic record systems (with patient consent).

An economic patient questionnaire (service use questionnaire) to collect the service use information from service users will be adapted from those used in previous mental health evaluations. Service users will be asked for information about the number of care planning meetings they have attended. Data will also be collected from health records (with consent) about the resources (staff and facilities) used in the care planning process. The data from the service use questionnaire will be combined with the data in the health records to allow a detailed description of the service use and costs associated with care planning.

The time and expenses of service users, carers and staff involved in providing and receiving the training intervention will be documented along with details of facilities used. These data will be used to estimate the total cost of the training package. For the primary analysis, the cost of the training package will be allocated to trial participants by dividing the total cost by the number of participants randomised to the intervention group. This assumes that the investment in the training intervention lasts for six months (and has no impact after that time) and will only benefit the staff

trained and the participants in the trial. This may over-estimate the costs of the training package if the training has a longer effect on the staff trained and/or the staff trained apply their training to more participants than they see in the trial. The training may also have a wider effect than just on the staff trained and change care planning/service provision for wider group of staff. The effect (on the cost effectiveness of the intervention) of changing assumptions about the duration of effect and number of service users/carers affected will be explored in sensitivity analyses.

The main measure of health benefit will be the quality adjusted life year (QALY), in line with the perspective adopted and NICE guidelines (NICE 2013). QALYs will be estimated from survival and health status measured by the EQ-5D-5L (Brooks 1996, Herdman et al, 2011, Janssen et al 2012). The EQ-5D-5L is a validated generic health status measure, used in national health surveys in the United Kingdom and in clinical trials in mental health, covering five domains (mobility, self-care, usual activity, pain/distress, and anxiety/depression). The EQ-5D-5L has been used extensively in mental health evaluations and demonstrated to identify small but consistent differences between groups. It correlates well with clinical outcome measures and has the potential to capture the impact of an intervention on physical as well as mental health (Davies et al 2007; Davies et al 2008; Ntais et al 2013). The EQ-5D-5L is designed as a self-report measure and will be completed by trial participants at baseline and follow up.

The five level version will be used (no problems, slight problems, some problems, severe problems or unable to do activity). Utility values, that reflect preferences for different health states, will be derived from the published utility tariffs developed for the 5 level instrument. QALYs will be estimated as:

$$\text{QALY} = \Sigma[(U_i + U_{i+1}) /2] \times (t_{i+1} - t_i)$$

where U = utility value and t = number of days between assessments.

Within trial primary analysis

The primary measure for the economic analysis will be the incremental cost effectiveness ratio (ICER). Accordingly, no statistical tests of differences in mean costs or outcomes will be conducted. The ICER will be estimated as the:

$$\frac{\text{Cost}_{\text{intervention}} - \text{Cost}_{\text{usual care}}}{\text{Utility}_{\text{intervention}} - \text{Utility}_{\text{usual care}}}$$

The estimates of incremental costs and outcomes from the regression will be bootstrapped to simulate 10,000 pairs of net cost and net outcomes of the intervention group for a cost effectiveness acceptability analysis, as recommended by NICE for health technology appraisals (National Institute for Clinical Excellence, 2013). These simulated data will be used to estimate the probability that service user/carer led training and care planning is cost effective compared to usual care.

This approach re-values effects or benefits in monetary terms. However, in the UK there is no universally agreed monetary value for the types of benefit measures used in cost effectiveness analyses. An approach used in health care is to ask the question: what is the maximum amount decision makers are willing to pay to gain one unit of benefit? The simulated net utility values were revalued using a range of maximum willingness to pay values from £1 to £30,000 to gain one unit of outcome, based on the range of willingness to pay values implied by NICE decisions (Rawlins et al, 2004).

The data for the cost effectiveness acceptability curve will be derived by first revaluing each of the 10,000 net outcome scores from the bootstrap simulation by a single WTPT. This is repeated for each WTPT. A net benefit statistic (NB) for each pair of simulated net costs and net outcomes for each WTPT can then be calculated as

*NB = (O * WTPT) – C, where O = net outcome score and C = net cost.*

This calculation is repeated for each WTPT. Cost-effectiveness acceptability curves plot the proportion of bootstrapped simulations where the net benefit of an intervention is greater than zero for each WTPT (Fenwick et al, 2001; Sendi et al, 2001; Hoch et al, 2002; Briggs et al, 2001).

Assuming a 'cluster cohort' design is used factors known to influence costs and QALYs (e.g. ethnicity, socio economic status, previous service use) will be collected at baseline to statistically control for their impact (Davies 2007; 2008). A linear mixed model with a random effect for cluster will be used in all trial based analyses to control for these.
Descriptive analysis and data manipulation will be conducted using SPSS, and the main statistical analyses and estimation of net benefit statistics and cost-effectiveness acceptability analysis will be conducted using STATA.

Within trial sensitivity analyses

Sensitivity analyses will explore whether the conclusions of the primary analysis will change in the following cases

Alternative assumptions about the duration and breadth of the effect of training are used to estimate the cost per participant of the intervention

The primary measures of outcome for the clinical evaluation are used as the measure of health benefit to estimate the ICER

The costs and QALYs are extrapolated to 12 months.

Case 1 will assume that the cost per day of health and social care estimated for the 3 month follow up is constant over the following 9 months and that the health status and utility value estimated at the 3 month follow up is constant over the following 9 months

Case 2 will assume that the cost per day of health and social care estimated for the 3 month follow up declines over the following 9 months and that the health status and utility value estimated at the 3 month follow up is constant over the following 9 months

Case 3 will assume that the cost per day of health and social care estimated for the 3 month follow up declines over the following 9 months and that the health status and utility value estimated at the 3 month follow up also declines over the following 9 months

Case 4 will assume that the cost per day of health and social care estimated for the 3 month follow up increases over the following 9 months and that the health status and utility value estimated at the 3 month follow up is constant over the following 9 months

Case 5 will assume that the cost per day of health and social care estimated for the 3 month follow up increases over the following 9 months and that the health status and utility value estimated at the 3 month follow up also increases over the following 9 months

Case 6 will assume that the cost per day of health and social care estimated for the 3 month follow up increases over the following 9 months and that the health status and utility value estimated at the 3 month follow up declines over the following 9 months

Economic model

An economic model will be developed to explore the impact of the intervention over alternative time periods, in different settings and populations. The model structure will be developed from a focussed review of the economics literature about care planning and training and refined/validated by discussion with EQUIP research team. Data to populate the model will be derived from the focussed review of the economics literature, review of national databases and datasets (e.g. Hospital Episode Statistics) and the trial. The primary and sensitivity will use incremental cost effectiveness and cost effectiveness acceptability approach outlined for the within trial evaluation. Probabilistic sensitivity analysis will be used to assess the level of uncertainty due to the data. Deterministic sensitivity analysis will be used to explore the impact of structural uncertainty.

Study 7

We will examine the processes involved in the development and adoption of user/carer involved care planning drawing on Normalisation Process Theory (NPT, May, 2007). NPT (which comprises of four components: coherence (sense making work), cognitive participation (relational work) , collective action (operational work), reflexive monitoring (appraisal work) has been developed from empirical studies of the implementation of complex interventions in health care contexts and in relation to mental health contexts in particular. We will focus on: (a) implementation of user/carer involved care planning - the way this is developed and translated into practices (of mental health professionals, users, carers and others); (b) embedding - the manner in which care planning becomes, (or does not become), routinely incorporated in everyday work of service users and professionals;(c) integration - how care planning is sustained as part of the everyday lives of individuals at work and at home d) networking - how it generates access to new networks and resources.

Analysis of interview and observational data will be conducted with reference to principles of the constant comparative method (Charmaz, 1995) whereby analysis will

be carried out concurrently with data collection so that emerging issues can be explored iteratively. Anonymised verbatim transcripts of audio recordings will be imported into the software package Atlas.ti for data management. Analysis will draw upon the techniques of grounded theory approaches (Glaser and Strauss, 1967) including initial coding of text segments, followed by re-coding and memo writing to generate conceptual themes driven by the Normalisation Process Theory (NPT). The transcripts will be read by at least two researchers. Themes (based on the four constructs of NPT) will be compared within and across cases, paying particular attention to negative cases and possible reasons for differences. In addition to the thematic analysis, an exploration of narratives will be valuable for the longitudinal study of the impact of care planning on individual cases. Both thematic and narrative approaches have been prominent in previous qualitative studies focused on the day-to-day living with mental health conditions, and will serve as complementary analytic techniques (Coffey and Atkinson, 1996). Analysis of diary records and observational data will be complementary to the above and where appropriate will be used to illustrate relevant issues emerging from the interviews or observation records and may be used to help elicit interview data.

User engagement

Study 5 and 7

Service users and carers have been involved with key aspects of the trial and process evaluation development, including helping to develop and deliver the user/carer led training in care planning, deciding on the secondary outcome measures to be used and development of the PROM.

Governance and Ethical issues

The key ethical concerns for the programme include confidentiality, participant anonymity and informed consent to participate in research. The study includes both mental health service users and carers as participants and as such there are specific ethical issues to be considered. Research governance principles and ethical committee approvals bind all applicants and their institutions. We will ensure we adopt the highest standards of research conduct including involvement of service user representation in both the management and delivery of the research.

This studies will be conducted in compliance with the study protocol, GCP and both University and NHS regulatory and monitoring requirements. The work stream teams will meet every three months and the CI will be responsible for the overall leadership, management and outputs of the programme. The PI from each site will maintain a log of the key milestones to be achieved against the timetable. The work stream leads will be responsible for the day to day running and co-ordination of the studies and will be accountable to the PI. All research associates will be supervised by the work stream leads.

Possible risks and anticipated benefits for research participants and society

The overall aim of the programme grant is to improve user and carer involvement in care planning in mental health services. Despite the fact that the majority of mental health policy documents, literature on best practice and literature produced by user and carer groups advocate that involving users and carers in care planning is fundamental to improving the quality of care and promoting recovery, there is substantial evidence that this does not always occur. This research is important because it provides an opportunity to make a quality improvement across community mental health services, such a quality improvement has the potential to be translated over both mental health and physical care settings and hence benefit many thousands of service users.

A full risk assessment of the EQUIP study will be undertaken prior to its commencement (Appendix 2).

Study 5

Completing the measures is not perceived as being high risk but there is always a risk that service users/carers may become distressed when thinking about difficult personal experiences. This risk has been assessed in the overall risk assessment for the work stream and as a result sources of further support will be included at the end of the questionnaires to ensure that participants have access to a source of support should they require it. This information will also be provided in participant information sheets

Data collection may require the researcher to visit participants at their work places or their homes. The School of Nursing, Midwifery and Social Work's Lone Worker Policy (Appendix 3) will be employed, as well as project specific risk assessment (Appendix

2). These involve research staff leaving details of their visits with a supervisor who they contact before and after the visit and using 'PeopleSafe' technology. When NHS CSOs carry out data collection they will follow their NHS Trust lone worker policies.

There is a risk to researchers that they might become distressed when collecting sensitive data within questionnaires. This has been assessed as a low risk but interviewers will have access to their supervisors for support on a regular basis and as required. NHS CSOs will have access to their supervisors within the NHS for support as well as regular contact with the EQUIP Programme Managers and Clinical Leads.

Informing potential participants of possible risks and anticipated benefits

All potential participants will be provided with an information sheet written to current NRES guidelines and favourably reviewed by the relevant ethics committee, prior to the study commencing. Service users and carers have been involved in developing the participant information sheets to ensure they are accessible. The information sheet will be provided to potential participants at the point of them expressing an interest in participating. It will provide potential participants with information about the study, including the potential benefits and risks of taking part, confidentiality and the right to withdraw as described above. Researcher contact details will be provided so participants can contact them with any queries prior to the participant deciding to take part. Researchers will further discuss risks and benefits immediately prior to the data collection taking place.

Obtaining informed consent

The exact consent methods to be used in the trial have been discussed in detail in the methods section. Prior to commencement of the study, the purpose and process of the study will be explained to the service users (cohort) and any questions raised will be addressed, before they sign the consent form. Service users (cross-section) and carers (cohort) will have the opportunity to have the purpose and process of the study explained to them and any questions addressed. Consent will be implied by return of the completed questionnaire and the consent to contact at follow up form (for carers).

Participants may change their mind and withdraw from the study at any point and this will not affect the care they receive.

Documentation and data management

All data will be stored securely in line with local data management arrangements. All questionnaires and other paper records will be stored in secure storage facilities at the University of Manchester and the University of Nottingham. Personal identifiable paper records will be stored separate from anonymised paper records. All electronic records will be pseudo-anonymised using a reference number for each participant and stored on a password protected server at the University of Manchester. Consent forms and other paper records will be stored as essential documents in a locked cabinet on University premises until the end of the project, at which point they will be archived until five years after the last publication arising from the study, or ten years after the programme grant completion, whichever is the greatest. All participant contact information will be destroyed securely and immediately at the end of the trial.

Study 7

Semi-Structured In-Depth Interviews

Service users and professionals

Many people enjoy being interviewed although there is also always a risk that people may become distressed when describing difficult personal experiences which may be the case during the interviews with service user and carers / family member participants. This risk has been assessed in the overall risk assessment for the work stream and as a result the research has an interview distress policy and debriefing sheet (appendix 4 and 5) to ensure that participants are supported both during and after group participation, if this should become necessary. The interviewers and their supervisors are sensitive to these issues and are experienced at supporting people experiencing distress.

Visiting people at home carries an additional risk and the School of Nursing, Midwifery and Social Work's Lone Worker Policy will be employed, as well as project specific risk assessments. These involve interviewers leaving details of their interview with a supervisor who they contact before and after the interview and using the 'PeopleSafe' system (http://peoplesafe.co.uk/). In the event of NHS CSOs carrying out data collection they will follow their individual NHS Trust Lone Worker policies.

There is a risk to interviewers that they might become distressed by listening to interviewee experiences. This has been assessed as a low risk but interviewers will have access to their supervisors for support on a regular basis and as required.

There is a potential risk that health professionals or service users may disclose examples of bad practice or risk of harm. We will follow ethical and legal practice and all information provided by participants will be handled in confidence. However, any suggestions of serious harm to self or others that is disclosed during the interviews cannot be treated as confidential. Where information given in a research context suggests that there is a threat of serious harm to the participant or others, researchers will disclose this to the relevant authorities, but also inform the participants and their guardians/responsible others of their intentions and reasons for doing so. Contemporaneous notes will be kept in case a complaint arises. Professor Karina Lovell has been nominated as the first point of call for researchers working on the project who will advise researchers as to the relevant authorities that need to be contacted and support the researcher and participant as necessary. We have identified two clinical leads in both study sites who will further facilitate this process should this become necessary

Observation

Service users and professionals

It is not anticipated that that there are risks associated with the observation of care planning meetings as these meetings would have occurred anyway without observation. However, if participants do become upset or uncomfortable during the observation the distress protocol will be followed. Furthermore and as above there may be disclosure of bad practice or risk of harm which would be addressed in the same way as above.

There is also a risk that one party will not agree to being observed. In order for the observation to take place, consent must be obtained for all the people taking part in the care planning meetings. If one party does not consent, participants will be offered the opportunity to take part in an interview after a care planning meeting to share their views.

Diaries

Service users

There is a risk that diaries will not be completed adequately or at all. In order to combat this researchers recruiting participants to the study will explain the value of completing the diary sheets to the study and participants will be offered the choice of either a written or audio version of the diary. In addition, a flexible approach to diary completion will be undertaken and participants will be asked to complete diaries at their discretion and will not be subject to a strict structure to encourage completion. In addition, we have other methods to capture this data if it is not recorded in the diary (e.g. social network methods and semi-structured interviews).

Informing potential participants of possible risks and anticipated benefits

All potential participants will be provided with an information sheet written to current NRES guidelines and favourably reviewed by the relevant ethics committee, prior to the study commencing. Service users and carers have been involved in developing the information sheet to ensure it is accessible. The information sheet will be provided to potential participants at the point of them expressing an interest in participating. It will provide potential participants with information about the study, including the potential benefits and risks of taking part as described above. Researchers contact details will be provided so participants can contact them with any queries prior to the participant deciding to take part. Interviewers will further discuss risks and benefits immediately prior to the interview taking place.

Obtaining informed consent

Semi-Structured in-depth interviews/observations/diaries: There will be several days between the potential interviewee receiving the information about the study (via invitation letter distributed at point of consent for trial or sent by the Trust with MHRN support) and the interview/observation/diary completing taking place. At the beginning of the visit the purpose and process of the study will be explained again, before potential participants are asked to sign a consent form. Participants may change their mind and withdraw from the interview at any point and they will be informed of this. Consent forms will be stored as essential documents in a locked cabinet on University premises until the end of the project, at which point they will be archived until five years after the last publication arising from the study, or ten years after the project's completion, whichever is the greatest.

Documentation and data management

All data will be stored securely in line with local data management arrangements. All interviews will be digitally recorded using encrypted digital recorders, with the interviewee's consent, and transcribed verbatim by a transcription company who have a confidentiality agreement with the University (appendix 7). If participants prefer for the interview to not be digitally recorded then the researcher undertaking the interview will take detailed notes. The audio files will be uploaded onto a University password protected server and then deleted from the digital recorder. All paper records will be stored in secure storage facilities at the University of Manchester. Personal identifiable paper records will be stored separate from anonymised paper records. All electronic records will be pseudo-anonymised using a reference number for each participant and stored on a password protected server at the University of Manchester. Interview transcripts will be pseudo-anonymised and stored on a University password protected server. At the end of the study transcripts will be archived until five years after the last publication arising from the study, or ten years after the project's completion, whichever is the greatest. All participant contact information will be destroyed securely and immediately at the end of the trial.

Timetable

From	To	Task
Apr-14	Apr-14	Ethics and R&D submission
May-14	May-14	Ethics and R&D approval
Jun-14<br><br><br><br><br><br><br>Jun-14	May-15<br><br><br><br><br><br><br><br>March-16	Study 5<br>Sequential cluster recruitment (2 cluster per month, 24 total)<br>Identification of eligible service users<br>Stratified randomisation by site<br>Service user consent to contact<br><br>Study 7<br>Recruitment/interviewing/observation/analysis
Jun -14	May -15	Study 5<br>Cohort baseline assessment<br><br>Study 7<br>Recruitment/interviewing/observation/analysis
Jul-14	June -15	Study 5<br>EQUIP training<br><br>Study 7<br>Recruitment/interviewing/observation/analysis

Dec-14	Nov-15	Study 5<br>Cohort 6 month follow-up assessment<br><br>Study 7<br>Recruitment/interviewing/observation/analysis
Feb -15	Dec -15	Study 5<br>Cross-sectional survey<br><br>Study 7<br>Recruitment/interviewing/observation/analysis
Aug -16	Dec -16	Final analysis and dissemination of results

References

Allen D, Griffiths L, Lyne P. Understanding complex trajectories in health and social care provision. Sociology of health & illness. 2004;26(7):1008–30.

Barczak P, Kane N, Andrews S, Congdon AM, Clay JC and Betts T (1988) Patterns of psychiatric morbidity in a genito-urinary clinic. A validation of the Hospital Anxiety Depression Scale. British Journal of Psychiatry 152: 698-700.

Bjelland I, Dahl AA, Tangen Haug T and Neckelmann (2002) The validity of the Hospital Anxiety and Depression Scale. An updated literature review. Journal of Psychosomatic Research 52, 69-77.

Blenkiron P, Mo KH, Cuzen J and Hammill AC (2003) Involving service users in their mental health care: the CUES project. Psychiatric Bulletin 27(9) 334-338

Bramesfeld A, Wedegartner F, Elgeti H and Bisson S (2007) How does mental health care perform in respect to service users expectations? Evaluating inpatient and outpatient care in Germany with the WHO responsiveness concept. BMC Health Services Research 7, 99

Briggs, A.H. &O'Brien,B. J. (2001) The death of cost minimisation analysis? Health Economics, 10,179-184.

Brooks R. EuroQoL: the current state of play. Health Policy. 1996;37:53-72.

Campbell, M., Fitzpatrick, R., Haines, A., Kinmonth, A. L., Sandercock, P., Spiegalhalter, D. et al. Framework for design and evaluation of complex interventions to improve health. British Medical Journal. 2000;*321:* 694-696.

Charmaz K. Grounded theory. In Smith J, Harre R and Van Lagenhave L (eds) Rethinking Methods in Psychology. London: Sage, 1995: 27-49.

Coffey AJ, Atkinson PA. Making sense of qualitative data: Complementary research strategies [Internet]. Sage Publications, Incorporated; 1996 [cited 2012 Oct 25]. Available from: http://books.google.co.uk/books?hl=en&lr=&id=lstE3peAdPMC&oi=fnd&pg=PR9&dq=coffey+atkinson+making+sense+of+qualitative+data&ots=posIkzzzvA&sig=sBSAMpa38vsCTCcIjMsG3R9r3Wc

Conrad P. Qualitative research on chronic illness: a commentary on method and conceptual development. Social Science & Medicine. 1990;30(11):1257–63.

Davies LM, Lewis S, Jones PB, Barnes TR, Gaughran F, Hayhurst K, Markwick A, and Lloyd H (2007). Cost effectiveness of first generation antipsychotics to treat psychosis: results from a randomised controlled trial. *British Journal of Psychiatry* 2007; 191: 14-22. doi: 10.1192/bjp.bp.106.028654

Davies LM, Barnes TRE, Jones PB, Lewis S, Gaughran F, Hayhurst K, Marwick A and Lloyd H. (2008) A randomised controlled trial of the cost utility of second generation antipsychotics in people with psychosis and eligible for clozapine. *Value in Health* 2008; doi: 10.1111/j.1524-4733.2007.00280.x

Department of Health, The NHS Plan: A plan for investment, a plan for reform. 2000, London: DH.

Dinniss, S., Roberts, G., Hubbard, C., Hounsell, J. and Webb, R. (2007) User–led assessment of a recovery service using DREEM. *Psychiatric Bulletin,* 31, 124–127.

Edwards S, Braunholtz D, Lilford R, Stevens A. Ethical issues in the design and conduct of cluster randomised controlled trials. *BMJ* 1999;**318**:1407-9.

Eldridge S, Ashby D, Feder G. Informed patient consent to participation in cluster randomized controlled trials: an empirical exploration of trials in primary care. *Clinical Trials* 2005;**2**:91-8.

EuroQol group. EuroQol—a new facility for the measurement of health related quality of life. Health Policy 1990;16:199–208.

Fenwick E, Claxton K, Sculpher M. Representing uncertainty: The role of cost-effectiveness acceptability curves. Health Economics. 2001;10(8):779-87.

Furber C (2010) Framework analysis: method for analysing qualitative data. African Journal of Midwifery and Women's Health, Vol. 4, Iss. 2, 16 Apr 2010, pp 97 - 100

Gaston L (1991) Reliability and criterion-related validity of the California Psychotherapy Alliance Scales – patient version. Psychological Assessment: A journal of Consulting and Clinical Psychology 3 (1) 68-74.

Gaston L and Marmar CR (1994) The California Psychotherapy Alliance Scales. The Working Alliance, Theory, Research and Practice. Eds Horvath AO and green berg LS. Wiley and Sons Inc. Pgs 85-109.

Gately C, Rogers A, Kirk S, McNally R. Integration of devices into long-term condition management: a synthesis of qualitative studies. Chronic illness. 2008;4(2):135–48.

Glaser BG, Strauss AL. The discovery of grounded theory: Strategies for qualitative research [Internet]. Aldine de Gruyter; 1967 [cited 2012 Oct 25]. Available from: http://books.google.co.uk/books?hl=en&lr=&id=rtiNK68Xt08C&oi=fnd&pg=PA1&dq=glaser+strauss+grounded+theory&ots=UUySTmXJ_I&sig=fV5VL9Wmf6AZCvjjfjsx1cg6_Ss

Greenhalgh T, Robert G, Bate P, MacFarlane F, Kyriakidou O (2005) Diffusion of innovations in health service organisations. Oxford: Blackwell

Gremigni P, Sommaruga M and Peltenberg M (2008). Validation of the Health Care Communication Questionnaire (HCCQ) to measure outpatients' experience of communication with hospital staff. Patient Education and Counselling 71: 57-64.

Healthcare Commission, The pathway to recover: a review of NHS acute inpatient mental health services. 2008, London: Healthcare commission.

Herdman M, Gudex C, Lloyd A, Janssen M.F, Kind P, Parkin D, Bonsel G, Badia X (2011). Development and preliminary testing of the new five-level version of EQ-5D (EQ-5D-5L). *Quality of Life Research*. December, Volume 20, Issue 10, pp 1727-1736. DOI 10.1007/s11136-011-99031

Hoch JS, Briggs AH, Willan AR. Something old, something new, something borrowed, something blue: a framework for the marriage of health econometrics and cost-effectiveness analysis. Health Economics. 2002;11(5):415-30.

Hutton J. Are distinctive ethical principles required for cluster randomized controlled trials? Stat Med 2001;**20**:573-488.

Janssen,M.F., Pickard,A.S., Golicki,D., Gudex,C., Niewada,M., Scalone,L., Swinburn,P., Busschbach,J. Measurement properties of the EQ-5D-5L compared to the EQ-5D-3L across eight patient groups: a multi-country study. Qual Life Res. 2013 Sep;22(7):1717-27. doi: 10.1007/s11136-012-0322-4. Epub 2012 Nov 25

Kennedy A, Bower P, Reeves D, Blakeman T, Bowen R, Chew-Graham C, Eden M, Fullwood C, Gaffney H, Gardner C, Lee V, Morris R, Protheroe J, Richardson G, Sanders C, Swallow A, Thompson D and Rogers A. Implementation of self management support for long term conditions in routine primary care settings: cluster randomised controlled trial. *BMJ* 2013;**346**:f2882.

Lelliott, P., Beevor, A., Hogman, G., Hyslop, J., Lathlean, J. and Ward, M. (1999). *The CUES Project: Carers' and Users' Expectations of Services. Final Report to the Department of Health.* London: Royal College of Psychiatrists' Research Unit.

Lelliott P, Beevor, A., Hogman, G., Hyslop, J., Lathlean J., and Ward M (2003) Carers' and users' expectations of services – carer version (CUES-C): A new instrument to support the assessment of carers of people with a severe mental illness. Journal of Mental Health 12, 2, 143-152

Lathlean, J. and Ward, M. (1999). *The CUES Project: Carers' and Users' Expectations of Services. Final Report to the Department of Health.* London: Royal College of Psychiatrists' Research Unit.

Ludman EJ, Simon GE, Rutter CM, Bauer MS and Unutzer J (2002) A measure for assessing patient perception of provider support for self-management of bipolar disorder. Bipolar disorders 4: 249-253

MacFarlane A, O'Donnell C, Mair F, de Brun M, de Brun T, Spiegel W, van den Muijesenbergh M, van Weel-Baumgarten E, Lionis C, Burns N, Gravenhorst K, Princz C, Teunissen E, van den Driessen Mareeuw F, Saridaki A, Papadakaki M, Vlahadi M and Dowrick C. (2012) Research into implementation Strategies to support patients of different Origins and language background in a variety of European primary care settings (RESTORE): study protocol. Implementation Science, 7: 12.

Mair FS, Hiscock J, Beaton SC. Understanding factors that inhibit or promote the utilization of telecare in chronic lung disease. Chronic Illness. 2008;4(2):110–7.

May C, Finch T, Mair FS, Ballini L, Dowrick C, Eccles E, Gask L, MacFarlane A, Murray E, Rapley T, Rogers A, Treweek S, Wallace P, Anderson G, Burns J and Heaven B. (2007). Understanding the implementation of complex interventions in health care: the Normalization Process Model. BMC Health Services Research; 7:148.

Mind (2010) Hear I am: Mental Health Service Users, Ward Life and Relationships. Social Action for Health: London

National Institute for Clinical Excellence. Guide to the Methods of Technology Appraisal: NICE; 2013.

NIHR Mental Health Research Network (2010) Who decides the definition of a 'good outcome'. MHRN: London.

Ntais D, Camacho E, Davies LM et al. Economic evaluation of collaborative care for people with depression and CHD or diabetes. To be submitted to BJP 2013

Pahl R and Spencer L. Capturing personal communities. In: Phillipson C, Allan G, Morgan D (eds) Social networks and social exclusion: sociological and policy perspectives. Aldershot: Ashgate, 2004, pp.72–96.

Patton MQ. Qualitative research & evaluation methods [Internet]. Sage Publications, Incorporated; 2001 [cited 2012 Oct 25]. Available from:

http://books.google.co.uk/books?hl=en&lr=&id=FjBw2oi8El4C&oi=fnd&pg=PP23&dq=patton+qualitative+evaluation+and+research+methods+sage+1990&ots=buq_cFHGrJ&sig=C1qyctTvc1SF7vtjV9cM3DOffBg

Rasch, G. (1960/1980). *Probabilistic models for some intelligence and attainment tests*. (Copenhagen, Danish Institute for Educational Research), expanded edition (1980) with foreword and afterword by B.D. Wright. Chicago: The University of Chicago Press.

Rawlins MD, Culyer AJ. National Institute for Clinical Excellence and its value judgments. British Medical Journal. 2004;329(7459):224-7

Richards DA, Hill JJ, Gask L, Lovell K, Chew-Graham C, Bower P, Cape J, Piling S, Araya R, Kessler, Bland M, Green, Gilbody S, Lewis G, Manning C, Hughes-Morley and Barkham M (2013). Clinical effectiveness of collaborative care (CADET): cluster randomised controlled trial. BMJ 2013;347:f4913.

Ridway P and Press A (2004) A user's guide for the developing recovery enhancing environments measure (DREEM). UK version 1. Eds Alott P and Higginson P.

Ritchie, J., L. Spencer, and W. O'Connor, Carrying out qualitative analysis, in Qualitative research practice. A guide for social science students and researchers, J. Ritchie and J. Lewis,
Editors. 2003, Sage: London.

Rogers A, Kirk S, Gately C, May CR, Finch T. Established users and the making of telecare work in long term condition management: Implications for health policy. Social Science & Medicine. 2011;72(7):1077–84.

Royal College of Physicians, Advance care planning. Concise guidance to good practice, ed. L.Turner-Stokes and B. Higgins. 2009, London: Royal College of Physicians.

Ruggeri M and Dall'Agnola R (1993) The development and use of the Verona Expectations for Care Scale (VECS) and the Verona Service Satisfaction Scale (VSSS) for measuring expectations and satisfaction with community-based psychiatric services in patients, relatives and professionals. Psychological Medicine, 23, 511-523

Ruggeri M., Lasalvia A., Dall'Agnola R., Tansella M., Van Wijngaarden B., Knudsen HC., Leese M. and Gaite L (2000) Development, internal consistency and reliability of the Verona Service Satisfaction Scale – European version: EPSILON Study 7. *British Journal of Psychiatry 2000, 177:s41-s48*.

Sendi PP, Briggs AH. Affordability and cost-effectiveness: Decision-making on the cost-effectiveness plane. Health Economics. 2001;10(7):675-80.

Tennant R, Hiller L, Fishwick R, Platt S, Joseph S, Weich S, Parkinson J, Secker J and Stewart-Brown (2007). The Warwick-Edinburgh Mental Well-being Scale (WEMWBS): development and UK validation. Health and Quality of Life Outcomes 5: 63.

The WHOQOL group (1998) The World Health Organisation quality of life assessment (WHOQOL): Development and general psychometric properties. Social Science and Medicine 46, 12, 1569-1585

Torgerson D. Contamination in trials: is cluster randomisation the answer? *BMJ* 2001;**322**:355-7.

Vassilev I, Rogers A, Blickem C, Brooks H, Kapadia D, Kennedy A, Sanders C, Kirk S, Reeves D. (2013). Social networks, the 'work' and work force of chronic illness self-management: a survey analysis of personal communities. PLoS One ;8(4):e59723.

Waddell L and Taylor M (2008) A new self rating scale for detecting atypical or second-generation antipsychotic side effects. Journal of Psychopharmacology 22 (3) 238-243.

World Health Organisation (2004) Quality of Life (WHOQOL) – BREF

World Health organisation (1997) WHOQOL Measuring Quality of Life. Programme on Mental Health WHO/MSA/MNH/PSF/97.4

Zigmond AS and Snaith RP (1983) The Hospital Anxiety and Depression Scale. Acta Psychiatrica Scandinavica 67 (6) 361-370.

Appendices

Appendix 1 – Training Protocol

Appendix 2 - Risk Assessment

Appendix 3 – Lone Worker Policy

Appendix 4 – Distress policy

Appendix 5 – Interview debriefing sheet

Appendix 6 – Consent to contact form

Appendix 7 – Transcription confidentiality agreement

Appendix 1 – Training Protocol

WS2 Training
Protocol(8.4.14).pdf

Appendix 2

Risk Assessment – Work stream 2, Study 5/Work stream 3, Study 7

Date: (1)	Assessed by: (2)	Checked/Validated by: (3)	Location: (4)	Assessment ref no (5)	Review date: (6)
2nd April 2014	Prof Pete Bower<br>Professor Anne Rogers	Karina Lovell (02.04.2014)	Face-to-face interviews/assessment/observations in NHS settings and participant homes and telephone interviews		

Task / premises: (7)
Researchers carrying out interviews/assessments/observations with service users and carers at the University and in NHS and community settings.

Activity (8)	Hazard (9)	Who might be harmed and how (10)	Existing measures to control risk (11)	Risk rating (12)	Result (13)
Business Travel	Journeys in Car	Research Staff<br>Journey travel at peak time risk of high volume of traffic on the road<br>Journey travel out of normal working hours<br>Risk of breakdown/injury/ accident due to adverse weather/other traffic/low light conditions	Researchers' handbook covers insurance information<br>Start and finish time, destination and route logged in shared calendar or similar.<br>PI/appointed member of staff is made aware of arrangements.<br>**Ensure business use is covered if using personal transport while lone working**	Low	A

Date: (1)	Assessed by: (2)	Checked/Validated by: (3)	Location: (4)	Assessment ref no (5)	Review date: (6)
2nd April 2014	Prof Pete Bower<br>Professor Anne Rogers	Karina Lovell (02.04.2014)	Face-to-face interviews/assessment/observations in NHS settings and participant homes and telephone interviews		

Task / premises: (7)
Researchers carrying out interviews/assessments/observations with service users and carers at the University and in NHS and community settings.

Activity (8)	Hazard (9)	Who might be harmed and how (10)	Existing measures to control risk (11)	Risk rating (12)	Result (13)
Travel to interview	Risk from neighbourhood	Researcher: from theft or assault	Researchers will familiarise themselves with address and route. Researchers will not use routes that are poorly lit or carry valuables. They will carry 'PeopleSafe' unit and mobile phone during all visits.	Low	A

Date: (1)<br>2nd April 2014	Assessed by: (2)<br>Prof Pete Bower<br>Professor Anne Rogers	Checked/Validated by: (3)<br>Karina Lovell (02.04.2014)	Location: (4)<br>Face-to-face interviews/assessment/observations in NHS settings and participant homes and telephone interviews	Assessment ref no (5)	Review date: (6)
Task / premises: (7)<br>Researchers carrying out interviews/assessments/observations with service users and carers at the University and in NHS and community settings.					

Activity (8)	Hazard (9)	Who might be harmed and how (10)	Existing measures to control risk (11)	Risk rating (12)	Result (13)
Visit to residential property	Personal Safety	Research staff<br><br>Physical/verbal abuse from patient/relative/<br>other	Detailed information on lone working on SNMSW Research Intranet.<br><br>**PeopleSafe unit used at all times.**<br>Research Staff will call appointment staff member/PI at time of entry to property, giving details of approximate time of length of interview and will call again upon leaving the property.<br><br>Neighbourhood being visits has been assessed to determine the likelihood of personal attack.<br><br>A list of high risk areas is held for locations where lone working visits will not be made in the hours of darkness.	Med	A

Date: (1)<br>2nd April 2014	Assessed by: (2)<br>Prof Pete Bower<br>Professor Anne Rogers	Checked/Validated by: (3)<br>Karina Lovell (02.04.2014)	Location: (4)<br>Face-to-face interviews/assessment/observations in NHS settings and participant homes and telephone interviews	Assessment ref no (5)	Review date: (6)

Task / premises: (7)
Researchers carrying out interviews/assessments/observations with service users and carers at the University and in NHS and community settings.

Activity (8)	Hazard (9)	Who might be harmed and how (10)	Existing measures to control risk (11)	Risk rating (12)	Result (13)
Recruitment/Carrying out interview	Risk of fire in location	Researchers and interviewees	Researchers will familiarise themselves with location regulations regarding fire and other emergencies. These will be communicated to the interviewee at the beginning of each interview if not own home.	Low	A

Date: (1)<br>2nd April 2014	Assessed by: (2)<br>Prof Pete Bower<br>Professor Anne Rogers	Checked/Validated by: (3)<br>Karina Lovell (02.04.2014)	Location: (4)<br>Face-to-face interviews/assessment/observations in NHS settings and participant homes and telephone interviews	Assessment ref no (5)	Review date: (6)
Task / premises: (7)<br>Researchers carrying out interviews/assessments/observations with service users and carers at the University and in NHS and community settings.					

Activity (8)	Hazard (9)	Who might be harmed and how (10)	Existing measures to control risk (11)	Risk rating (12)	Result (13)
Recruitment/Carrying out interview	Risk from interviewee/relative	Researcher: from verbal abuse, theft or assault	Researchers will be aware of signs of threat increasing aggression and take steps to diffuse situations or end the interview. Interviewers not to carry valuables. Personal information is not given out and the Patient Information Sheet directs Participants/Relatives to the PI and gives only the School Number. Appropriate measures are put in place to ensure any issues arising are dealt with via the appropriate channels.	Low	A

Date: (1)<br>2nd April 2014	Assessed by: (2)<br>Prof Pete Bower<br>Professor Anne Rogers	Checked/Validated by: (3)<br>Karina Lovell (02.04.2014)	Location: (4)<br>Face-to-face interviews/assessment/observations in NHS settings and participant homes and telephone interviews	Assessment ref no (5)	Review date: (6)
Task / premises: (7)<br>Researchers carrying out interviews/assessments/observations with service users and carers at the University and in NHS and community settings.					
**Activity (8)**	**Hazard (9)**	**Who might be harmed and how (10)**	**Existing measures to control risk (11)**	**Risk rating (12)**	**Result (13)**
Recruitment/Carrying out interview	Psychological Distress/Risk from disclosure	Researcher: stress arising from listening to distressing disclosures	Researchers are experienced in working with people with mental health problems. Interviewers are encouraged to discuss any issues with their line manager. All researchers are appropriately qualified with adequate experience. Adequate training e.g. Good Clinical Practice. Regular debriefs with PI. Counselling offered as needed	Low	A
Duration of interview and afterwards	Risk from distress from disclosure	Interviewee: risk of distress	Researchers are experienced in working with people with mental health problems and will have information regarding support they can pass on to interviewees. Protocols are in place for contacting clinical lead for study (Professor KL) who will liaise with support services.	Low	A

Date: (1)<br>2nd April 2014	Assessed by: (2)<br>Prof Pete Bower<br>Professor Anne Rogers	Checked/Validated by: (3)<br>Karina Lovell (02.04.2014)	Location: (4)<br>Face-to-face interviews/assessment/observations in NHS settings and participant homes and telephone interviews	Assessment ref no (5)	Review date: (6)

Task / premises: (7)
Researchers carrying out interviews/assessments/observations with service users and carers at the University and in NHS and community settings.

Activity (8)	Hazard (9)	Who might be harmed and how (10)	Existing measures to control risk (11)	Risk rating (12)	Result (13)
Working with NHS Staff/Patients	Identifying bad practice	Researcher<br><br>Impact on project/results/data collection/damage to reputation of staff/project/School/University/ NHS Unit	Action to be taken in this event covered by study protocol – Regular meetings with PI	Low	A

Date: (1)<br>2nd April 2014	Assessed by: (2)<br>Prof Pete Bower<br>Professor Anne Rogers	Checked/Validated by: (3)<br>Karina Lovell (02.04.2014)	Location: (4)<br>Face-to-face interviews/assessment/observations in NHS settings and participant homes and telephone interviews	Assessment ref no (5)	Review date: (6)
Task / premises: (7)<br>Researchers carrying out interviews/assessments/observations with service users and carers at the University and in NHS and community settings.					

Activity (8)	Hazard (9)	Who might be harmed and how (10)	Existing measures to control risk (11)	Risk rating (12)	Result (13)
Interviewing/data Collection	Personal Safety	Research Staff (as per Lone Working Policy)<br>Physical/verbal abuse from patient/relative/<br>other	Lone Working Policy – SNMSW.<br>Project specific Lone Worker Protocol has been written and full risk assessment undertaken by Programme Manager (CF) and PI (KL).<br>Researchers are experienced in working with people with mental health problems and will be informed of any issues which may arise prior to visiting. Protocols are in place for contacting clinical lead for study (Professor KL) who will liaise with support services.<br>Detailed information on lone working on SNMSW Research Intranet.<br>**Mandatory use of Rocksure Peoplesafe Lone working Unit**	Med	A

Date: (1)<br>2nd April 2014	Assessed by: (2)<br>Prof Pete Bower<br>Professor Anne Rogers	Checked/Validated by: (3)<br>Karina Lovell (02.04.2014)	Location: (4)<br>Face-to-face interviews/assessment/observations in NHS settings and participant homes and telephone interviews	Assessment ref no (5)	Review date: (6)
Task / premises: (7)<br>Researchers carrying out interviews/assessments/observations with service users and carers at the University and in NHS and community settings.					

Activity (8)	Hazard (9)	Who might be harmed and how (10)	Existing measures to control risk (11)	Risk rating (12)	Result (13)
Clerical/Computer Work	DSE related	Research Staff<br><br>RSI/musculoskeletal pain injuries.	Covered by Health & Safety Induction and Policy Documents<br><br>www.nursing.manchester.ac.uk/intranet/about/hs/	Low	A

Date: (1)<br>2nd April 2014	Assessed by: (2)<br>Prof Pete Bower<br>Professor Anne Rogers	Checked/Validated by: (3)<br>Karina Lovell (02.04.2014)	Location: (4)<br>Face-to-face interviews/assessment/observations in NHS settings and participant homes and telephone interviews	Assessment ref no (5)	Review date: (6)

Task / premises: (7)
Researchers carrying out interviews/assessments/observations with service users and carers at the University and in NHS and community settings.

Activity (8)	Hazard (9)	Who might be harmed and how (10)	Existing measures to control risk (11)	Risk rating (12)	Result (13)
Research Governance	Non-Compliance	Research Staff<br><br>Damage to reputation of staff/project/School/University.<br><br>Potential disciplinary action.	Research Study Monitoring Self-Assessment Form. Research Governance Policy and Procedure.	Med	A

**Action plan** (14)				
**Ref No**	**Further action required**	**Action by whom**	**Action by when**	**Done**
1	Lone Worker risk assessment to be reviewed and modified as project develops in line with any changes	Karina Lovell	Sept 2014	
2	Lone worker to be given training/training information for use of Lone Worker Device (contact Stacey.body@manchester.ac.uk)	Julie O'Rourke/ Programme Manager	June 2014	
3	Shared calendar or similar set up and filled in regularly informing PI/appointed research staff member of any visits	Julie O'Rourke/ Programme Manager	June 2014, then as needed	
4	Assessment of residential areas ensuring high risk areas are identified	Karina Lovell/ Programme Manager	As needed	
5	Escalation procedure devised with PI	Karina Lovell/ Programme Manager	June 2014	

Appendix 3: Lone worker policy

School of Nursing, Midwifery & Social Work
Safety Guidance for Research Fieldwork in the UK

Summary of Responsibilities of PI who have research staff doing fieldwork

- Ensure the '**SNMSW Safety Guidance for Research Fieldwork'** is provided to research staff and this is read and understood by the researcher;
- Complete the **SNMSW Lone Worker Risk Assessment Form** (provided in Appendix 1 of the Safety Guidance and also available on School's Staff Intranet (http://www.nursing.manchester.ac.uk/intranet/researchadministration/);
- Ensure that immediate access to 'Contact details' including details about the person to contact in case of emergency and agreed fieldwork contact plan is available out of hours;
- Ensure the researcher is clear on who to provide details of fieldwork visits to (this may involve having access to a shared/password protected calendar);
- Ensure cover arrangements are provided by the PI when on holiday or away from the University;
- If study involves fieldwork with individuals who pose a 'higher risk', please complete the Risk Assessment Questionnaire for high risk studies and modify original Lone Worker Risk Assessment provided in Appendix 2 of the Safety Guidance; also available on School's Intranet (http://www.nursing.manchester.ac.uk/intranet/researchadministration/)
- Mandatory use of Rocksure Peoplesafe Lone Worker Unit. (http://www.nursing.manchester.ac.uk/intranet/researchadministration/researchersafety)

Please ensure that PIs and researchers adhere to the 'Flowchart of process for monitoring lone working (Research Fieldwork)'

Appendix 4: Distress policy

EQUIP Distress Protocol

If a participant appears to become distressed, the researcher will:

- Encourage the participant to take a break from answering questions.
- Acknowledge that talking about/answering questions about problems can be distressing.
- Offer support by reassuring the service user that they do not need to answer a question(s) if they do not wish to.
- Ask if they would like to continue with the interview/completing questionnaires or prefer to stop.

If they prefer to stop then:

- Finish the interview/questionnaire completion and offer to return at another time.
- If the service user withdraws their consent to participate in the study then this must be communicated to the work stream lead or Programme Manager.

If the interview continues:

- Take time at the end of the meeting to talk informally, and encourage service users to access further support dependant on their level of distress. This may be to visit their GP or mental health service provider or refer service users to list of support agencies on the information sheet.
- If the service user has any questions or requires reassurance about the research they should be encouraged to contact the work stream lead or Programme Manager (details on Debriefing Sheet and Participant Information Sheet).
- If the participant still appears to be distressed when the interview is over then the researcher will offer to phone back in a couple of days to ensure the distress has not escalated and to reiterate the sources of support. If this is the case or there is a perceived risk of harm to self or others the site Clinical Lead will also be informed and a decision made on contacting the relevant authorities in line with the EQUIP risk protocol.

Appendix 5: Interview Debriefing Sheet

Enhancing the quality of user involved care planning in Mental Health Services (EQUIP)

The purpose of this interview was to explore your experiences of care planning and examine the potential facilitators and barriers to the implementation of user/carer-led planning within health services. The views and ideas that you have provided us with are extremely useful. We will use this information to write a report to help improve user/carer involvement in care planning across mental health services.

We hope that taking part in the interview has been interesting; although we also appreciate that talking about personal experiences can be difficult.

If you would like to contact us at a later date about any queries that you may have about your taking part please do not hesitate to contact us:

Lead researcher WS3
Professor Anne Rogers
Faculty of Health Sciences
University of Southampton
Highfield
Southampton
SO17 1BJ
Room Number: 67/E4009
Telephone: (023) 8059 6830
Email: A.E.Rogers@soton.ac.uk

EQUIP Programme Managers
Kathryn Berzins/Claire Fraser
School of Nursing, Midwifery & Social Work
University of Manchester, Jean McFarlane Building, Oxford Road, Manchester, M13 9PL
Tel: 0161 306 7893/0161 306 7882
Email: kathryn.berzins@manchester.ac.uk /Claire.fraser@manchester.ac.uk

We would like to take this opportunity to thank you for taking part in this interview. The information you have given us is very valuable and we appreciate your time.

Appendix 6: Consent to contact form

Enhancing the quality of user involved care planning in Mental Health Services (EQUIP)

If you think you would like to take part in the EQUIP study: <u>Evaluation of the efficacy and cost effectiveness of user/carer involved care planning</u>, please use this form to let us know. Please send it back to the research team using the pre-paid envelope provided.

A member of the research team will then contact you to discuss the study and if you wish to participate, will arrange an appointment to see you. There is no obligation to take part in this study by completing this form; you are simply providing your consent to be contacted.
We will not pass your details to anyone outside of the EQUIP team.

If you would prefer to talk to the research team before completing this form, please contact:
Kathyrn Berzins/Claire Fraser, EQUIP Programme Managers, School of Nursing, Midwifery & Social Work, University of Manchester, Jean McFarlane Building, Oxford Road, Manchester, M13 9PL
Tel: 0161 306 7893 / 0161 306 7882
Email: kathryn.berzins@manchester.ac.uk or claire.fraser@manchester.ac.uk

**I would like to be contacted about taking part in the EQUIP study: <u>Evaluation of the efficacy and cost effectiveness of user/carer involved care planning</u>**		
Name:		
Postal address:		
Post Code:		
Telephone number:		
Email:		
What is it best time to contact you?<br>(please indicate preference)	Morning/Afternoon/ Evening/ Don't Mind	
I would like to receive a copy of the results of this study	Yes please	No thank you

Appendix 7: Transcription confidentiality agreement

Extract from 1st Class Secretarial Client Charter

4. Client Confidentiality

1st Class handle all client data in the strictest confidence.

All files are managed via our secure client website.

The online service offered by 1st Class Secretarial Services is one which is offered across the entire client base and has a tiered series of security levels and access, which offer data protection and security on a number of levels.

1. Physical Location – the server which hosts the online service is located in a non-public accessible data bank and is not physically accessible by any staff other than those on the system administration level who have access to the hardware. We have written statements from the ISP (Internet Service Provider) which categorically confirms this.

2. Server Infrastructure - the website which serves as the interface for the transfer of data onto and from the server is located on a dedicated 64-bit Windows 2003 Web Server on a Tier One 1Gb network . All transfers of data are encrypted using a 128-bit Secure Socket Layer on an annually renewed certificate which is presented to all pages within the online service to offer encryption of data transfer for all clients. There are no other websites running from this physical server, it is a single-use dedicated server.

3. Permission Level Access – each client account is accessible only via an extranet system which is password protected so that a personalised interface is delivered to the client browser post-login and no details, files or account information are accessible between accounts.

4. Password Renewal - as an additional security measure, we encrypt client passwords using a 128 bit security algorithm. To enhance password security, clients can request to have their password changed on a 30 or 60 day cycle.

Our team of transcriptionists is issued with 2Gb FIPS encrypted flash drives. They sign an undertaking that they will:

- Download and save any sound files and associated Word documents allocated by 1st Class to the flash drive and not to their PC or laptop hard drive.
- Not save any work other than work allocated from 1st Class to this flash drive.
- Not change the allocated password.
- Not divulge the allocated password to anyone.
- Inform 1st Class immediately of any security compromise of the flash drive, including:
    - the password becoming known by another person;
    - the flash drive being lost or stolen;
    - any unauthorised access to the flash drive.
- Regularly delete sound and Word documents from the flash drive - on confirmation of receipt of the completed transcript by 1st Class.
- Remove the flash drive from the PC when not in use and to store it in a locked cupboard or safe.
- Not print completed transcripts wherever possible - or to adequately shred any printed transcripts as soon as the transcript has been returned and acknowledged by 1st Class.
- Return the flash drive by recorded delivery to 1st Class at the end of any working agreement with 1st Class.

All transcriptionists are required to sign the 1st Class confidentiality agreement and service level agreement prior to commencing work with 1st Class. They also provide photographic evidence of their identity, along with confirmation of their address via a utility bill or bank statement.

1st Class has designated signatories who have the authority to sign client confidentiality agreements on behalf of the company.

1st Class has voluntarily subscribed to the Data Protection Act, registered number Z2116676. We have also signed the Code of Practice on Data Handling. Sound files will be automatically deleted from the 1st Class webserver after four months. The sound file may be retained on the internal server for a longer period of time, normally no more than six months.

Transcripts will be automatically deleted from the 1st Class webserver and internal server after one year. It is the client's responsibility to ensure that they have a copy of

the original sound file and transcript. If the transcript is deleted from both the webserver and internal server and the client requires a copy, this will be transcribed again and the normal charges will apply.
